# Coding Gene Single Nucleotide Polymorphism Mapping and Quantitative Trait Loci Detection for Physiological Reproductive Traits in Brook Charr, *Salvelinus fontinalis*

**DOI:** 10.1534/g3.111.001867

**Published:** 2012-03-01

**Authors:** Christopher Sauvage, Marie Vagner, Nicolas Derôme, Céline Audet, Louis Bernatchez

**Affiliations:** * Institut de Biologie Intégrative et des Systèmes (IBIS), Département de Biologie, Université Laval, Québec (Québec) Canada, G1V 0A6; † INRA, UR1052, Unité de Génétique et d’Amélioration des Fruits et Légumes, 84143 Montfavet, France; ‡ Institut des sciences de la mer de Rimouski (ISMER), Université du Québec à Rimouski (UQAR), Rimouski (Québec) Canada, G5L 3A1; § Institut du Littoral et de l’Environnement, LIENSs UMR6250, 2 rue Olympe de Gouges, 17000 La Rochelle, France

**Keywords:** linkage mapping, QTL detection, single nucleotide polymorphisms (SNP), reproduction, *Salvelinus fontinalis*

## Abstract

A linkage map of 40 linkage groups (LGs) was developed for brook charr, *Salvelinus fontinalis*, using an F_2_ interstrain hybrid progeny (n = 171) and 256 coding gene SNP developed specifically for brook charr and validated from a large (>1000) subset of putative SNP, as well as 81 microsatellite markers. To identify quantitative trait loci (QTL) related to reproduction functions, these fish were also phenotyped at six physiological traits, including spermatozoid head diameter, sperm concentration, plasma testosterone, plasma 11-keto-testosterone, egg diameter, and plasma 17β-estradiol. Five significant QTL were detected over four LGs for egg diameter and plasma 17β-estradiol concentration in females, and sperm concentration as well as spermatozoid head diameter in males. In females, two different QTLs located on LG 11 and LG 34 were associated with the egg number, whereas one QTL was associated with plasma 17β-estradiol concentration (LG 8). Their total percent variance explained (PVE) was 26.7% and 27.6%, respectively. In males, two QTL were also detected for the sperm concentration, and their PVE were estimated at 18.58% and 14.95%, respectively. The low QTL number, associated with the high PVE, suggests that the variance in these reproductive physiological traits was either under the control of one major gene or a small number of genes. The QTL associated with sperm concentration, plasma 17β-estradiol, and egg diameter appeared to be under a dominance effect, whereas the two others were under a negative additive effect. These results show that genes underlying the phenotypic variance of these traits are under different modes of action (additive *vs.* dominance) and may be used to predict an increase or a decrease in their phenotypic values in subsequent generations of selective breeding. Moreover, this newly developed panel of mapped SNP located in coding gene regions will be useful for screening wild populations, especially in the context of investigating the genetic impact of massive stocking of domestic brook charr to support the angling industry throughout eastern North America.

Linkages maps have numerous applications in evolutionary and comparative genomics (Gharbi *et al.* 2006, Jaari *et al.* 2009). Namely, one of the main applications of linkage maps relies on the mapping of quantitative trait loci (QTL), which allows localizing genomic regions responsible for the variations in continuous phenotypic traits of interest. To this end, the number and the variety of molecular markers developed for linkage-mapping have been associated with technological advances, namely new marker development such as microsatellites and single nucleotide polymorphisms (SNPs). Also, compared with previous methods based on cloning and Sanger sequencing, next-generation sequencing technologies now allow the more rapid development of *in silico* SNP markers (Hudson 2008). Yet, SNP marker development and *in silico* validation remain particularly challenging in polyploid species as the result of recent whole-genome duplication events, such as in salmonids (Seeb *et al.* 2011).

Salmonids are of considerable economic, social, and conservation value (Davidson *et al.* 2010). The salmonidae family contains 11 genera and 68 species, including salmon, trout, charr, freshwater whitefishes, ciscos, and graylings (for review, see Davidson *et al.* 2010) and has experienced a whole-genome duplication approximately 25 to 100 million years ago (*e.g.*, Phillips and Rab 2001). More is known about the biology and genetics of salmonids than in any other groups of fish (Koop and Davidson 2008), which has led to the development of important genomic tools in recent years. For example, linkage maps have been successively developed in the Atlantic salmon *Salmo salar* to include up to 5650 markers (Danzmann *et al.* 2008; Moen *et al.* 2008; Lorenz *et al.* 2010, Lien *et al.* 2011). In rainbow trout (*Onchorynchus mykiss*), multiple generations of linkage maps have been developed using various types of molecular markers, including amplified fragment length polymorphisms (Young *et al.* 1998; Nichols *et al.* 2003), single-sequence repeats, or SSR (Rexroad *et al.* 2008), and SNP (Miller *et al.* 2011; Lien *et al.* 2011). Lower resolution linkage maps have also been developed in Arctic charr *Salvelinus alpinus* (Woram *et al.* 2004, Moghadam *et al.* 2007), coho salmon *Oncorhynchus kisutch* (McClelland and Naish 2008), brown trout *Salmo trutta* (Gharbi *et al.* 2006), lake whitefish *Coregonus clupeaformis* (Rogers *et al.* 2007), and recently, in brook charr, *Salvelinus fontinalis* (Timusk *et al.* 2011).

All these maps exhibit a large number of linkage groups, or LG (>28), because the diploid ancestor of salmonids possessed a karyotype with 48 acrocentric chromosomes (Phillips and Rab 2001) and a span of 390 to 5548 cM with a genome size estimated at 3.3 × 10^9^ bp (Davidson *et al.* 2010). The investigation of the evolution of the structural genome within the salmonidae family, highlighting chromosomal rearrangements notably between *Salvelinus* and *Onchorynchus* species (Timusk *et al.* 2011). From the available linkage maps, recent QTL investigations were performed for phenotypic traits of interest for production such as hatching time (Robison *et al.* 2001, Nichols *et al.* 2007, Miller *et al.* 2011, Easton *et al.* 2011), spawning date (O’Malley *et al.* 2003), length (McClelland and Naish 2010), growth (Nichols *et al.* 2008; McClelland and Naish 2010; O’Malley *et al.* 2010; Wringe *et al.* 2010), early male maturation (Fotherby *et al.* 2007; Haidle *et al.* 2008), resistance to pathogens (Moen *et al.* 2009; for review, see Palti 2009 and Nichols *et al.* 2010), thermal tolerance (Perry *et al.* 2001), and swimming behavior (Rogers and Bernatchez 2007).

Brook charr, *Salvelinus fontinalis*, is one of the most economically important species for freshwater aquaculture in Canada, where it is farmed both for food production and enhancing the sport fishery industry (Page and Burr 1991). In Québec (Canada), approximately 1200 tons are produced annually, which represents approximately 55% of the total aquaculture production. In this context, the aims of the present study were (1) to develop a set of validated SNP markers located in coding genes using RNA-seq, and thus provide the first SNP markers for the genus *Salvelinus*; (2) to build a linkage map for this species combining these newly developed SNPs and previously published microsatellites; and (3) to identify QTL detection for reproductive physiological traits of interest for aquaculture production. We also compare our genetic map with that recently published by Timusk *et al.* (2011).

## Material and Methods

### Biological material and fish crosses

The QTL population was created using two different populations of brook charr. The first one (D) is a domestic population used in aquaculture for more than 100 years in Québec (Canada). The other (L) is an anadromous population originating from the Laval River near Forestville (North of the St. Lawrence River, Québec, Canada). Breeders from the L population were kept in captivity for three generations at the Station aquicole de l’ISMER (Québec, Canada, 48°31′N, 68°28′W) whereas breeders from the D population were obtained from the Pisciculture de la Jacques Cartier (Cap-Santé, Québec, Canada). In 2005, 10 sires of each population (L and D; F_0_ generation) were crossed with 10 dams (L and D) to generate 10 full-sib outbreed hybrid families (LD) crosses (F_1_ generation). Then, three F_2_ families were obtained from the biparental cross of six F_1_ individuals. The selected F_2_ family used in the present study for both phenotyping and genotyping was chosen according to its lowest mortality rate (<2%) compared with the other ones and its greater number of individuals.

### Fish-rearing conditions

Fish from the F_2_ hybrid population hatched in January 2008 and were reared in indoor circular tanks in fresh water at the Station aquicole de l’ISMER (Québec, Canada, 48°31′N, 68°28′W), under natural photoperiod and temperature. The density was maintained < 35 kg of fish per m^3^. Fish were fed daily with commercial pellets with the percent weight per day adjusted according to fish age and water temperature (from 5.3% to 1.5% of body weight). They were individually identified with PIT-tags at the age of 5 months.

### DNA library preparation and cDNA sequencing for SNP development

In September 2008, liver tissue was collected from 22 fish, including eight individuals from the freshwater Rupert River population (Québec, 48°44′N, 68°05′W), eight individuals from the Laval River population (Québec, 51°05′N, 73°41′W) (Audet and Bernatchez 2004), and the six parents used to create the three F_2_ families. Tissue samples were kept at −80° at all times and total RNA was extracted using the Purelink Micro to Midi total RNA purification system (Invitrogen, Inc., ON, Canada). Two micrograms of mRNA were used to perform a full-length cDNA amplification and normalization (Evrogen, Moscow, Russia). The cDNA was synthesized and amplified following the SMART approach (Zhu *et al.* 2001) and normalized using the duplex-specific nuclease method (Zhulidov *et al.* 2004). A total of 5 µg of double-stranded cDNA was sequenced (half plate) on a GS-FLX Titanium sequencer at the Genome Québec Innovation Center (McGill University, Montréal, QC, Canada).

### Data assembly

Base calling was performed using PyroBayes software according to Quinlan *et al.* (2008). Primer tag sequences were removed, and sequence data were assembled into contiguous sequences using CLC Genomic Workbench v3.7 (CLC Bio, Denmark). Assembly parameters were set at 97% of similarity between two sequences showing an overlap of 0.6. Because of the duplicated genome of salmonids, such stringent assembly criteria were necessary to reduce probabilities of assembling paralogous genes (Renaut *et al.* 2010). Nonspecific reads (matching in different contigs) were discarded. Sequences that did not assemble into contigs were removed from further analyses to reduce the impact of sequencing errors. Raw pyroreads are publicly available under the accession number SRX037496 (http://www.ncbi.nlm.nih.gov/sra). Contig sequences, obtained from 454 data assembly, are provided in supporting information, Table S1.

### SNP detection, validation, genotyping, and annotation

Assembled contigs were screened for SNPs using CLC Genomic Workbench v3.7 with the following criteria: minimum SNP coverage at 10× and minor allele frequency at 20%; the remaining parameters were left as default. To validate the markers in the mapping population, a large subset of putative SNP (∼1000) was selected from the whole set of putative SNP detected in the contigs according to the following parameters: (1) polymorphic sites had to be surrounded by a fragment of 60 bp to allow the design of polymerase chain reaction (PCR) primers and the genotyping assay; (2) polymorphic sites were not linked to other polymorphic loci in the contig (may reveal the assembly of paralogous sequences).

We then applied four steps to validate a subset (>300) of these 1000 putative markers. (1) A pair of PCR primers surrounding each SNP of interest was designed using Primer 3 software (Rozen and Skaletsky 2000) to generate an amplicon of 250 to 400 bp. PCR amplicons were then ran and visualized on a 1.5% agarose gel. Primer pairs that showed an amplicon size greater than 400 bp were removed to avoid the amplification of intronic regions in subsequent genotyping steps. (2) Amplicons meeting step 1 requirements were sequenced on the six F_1_ mapping parents in forward and reverse sense using the Big Dye Terminator v3 chemistry (Applied Biosystems, Foster City, CA) to confirm every polymorphic site. Chromatograms were inspected by eye and every monomorphic site was removed from subsequent analysis. (3) iPlex Gold assays for the Sequenom MassARRAY (Sequenom, San Diego, CA) genotyping technology were designed. Sites that did not respect the Sequenom assay the following technical requirements were removed: flanking sequences of 50 bp around the site of interest, no other polymorphic site within these flanking sequences, and a GC content greater than 40% (See File S2).

For each validated SNP, we searched for functional annotation and the nature of the polymorphism (transition *vs.* transversion, synonymous *vs.* nonsynonymous mutation). BlastX similarity searches were performed with the NCBI protein database (nr) using the consensus sequences of each contig containing a validated SNP. Only *e*-values lower than 1 × 10^−40^ were considered significant. Then, open reading frame (ORF) predictor (Min *et al.* 2005) was used to predict the most probable ORF for contigs that exhibited a significant Blast hit. Finally, SNPs were characterized as synonymous or nonsynonymous from the deduced ORFs. (For details, see File S3.) (4) Only SNP that met all requirements were multiplexed for genotyping the F_1_ parents and their F_2_ progeny using the iPlex Gold assays on the MassARRAY platform (Sequenom, San Diego, CA) according to the manufacturer’s instructions at the Genome Québec Innovation Center (McGill University, Montréal, QC, Canada).

### DNA extraction and microsatellite genotyping in the F_2_ progeny

Genomic DNA was extracted from fin tissues (n = 191) of the selected F_2_ hybrid progeny using the QIAGEN DNeasy kit according to the manufacturer’s specifications (QIAGEN, Valencia, CA). DNA was quantified using the Quant-iT PicoGreen assay (Invitrogen, Inc., ON, Canada). For microsatellite genotyping, we first selected a set of 101 microsatellite markers that were available from the literature and previously used in the development of a linkage map in the brook charr (see Timusk *et al.* 2011). These markers cross-amplify in several salmonid species, including *Salmo salar*, *Onchorynchus mykiss*, *Salvelinus alpinus*, and *Salmo trutta* (Olsen *et al.* 1998; Sakamoto *et al.* 2000; Palti *et al.* 2002; Rexroad *et al.* 2003; McGowan *et al.* 2004; Rise *et al.* 2004; Coulibaly *et al.* 2005; Thorsen *et al.* 2005; Gharbi *et al.* 2006; Phillips *et al.* 2009). In addition, we included 25 additional microsatellites (Angers *et al.* 1995; O’Reilly *et al.* 1996; Scribner *et al.* 1996; Dehaan and Ardren 2005; Perry *et al.* 2005; see File S1 for details).

Only the informative markers were kept, that is, heterozygote for at least one of the two F_1_ parents. These were then multiplexed and amplified by PCR on a ABI 7900HT thermocycler (Applied Biosystems, Foster City, CA) according to the following cycling conditions: initial denaturation of 15 min at 95°, then 35 cycles of 30 sec of denaturation at 94°, 3 min of annealing at 60°, and 1 min of elongation at 72°, followed by a final extension step of 10 min at 72°. The reaction mixture was composed of 2 µL of gDNA (20−50 ng µL^−1^), 5 µL of Taq PCR Master Mix (QIAGEN, Inc., ON, Canada), and 0.2 µL of each primer (forward and reverse), for a final reaction volume of 8 µL. Forward PCR primers were FAM and HEX labeled with a fluorescent dye and multiplexed according to the size of the PCR products to avoid any overlap in the markers size ranges. PCR products from each sample were diluted 50-fold, and 1 µL of diluted PCR product was mixed with 0.25 µL of ROX GS500 size ladder (Applied Biosystems) and 10 µL of HiDi formamide. Samples were denatured at 95° for 3 min and kept on ice until loading on an ABI 3130 automatic sequencer (Applied Biosystems) according to the manufacturer’s recommendations. GeneMapper software V4.1 (Applied Biosystems) was used to analyze output data and collect microsatellite genotypes.

### Sampling and phenotyping

F_2_ progeny (1+ year-old fish) were first collected in August (n = 91; 223.9 ± 5.5 g) and then in November 2009 (n = 100; 276.7 ± 6.4 g) on 24-hour−fasted fish. After capture, fish were immediately placed in an anesthetic solution (3-aminobenzoic acid ethyl ester, 0.16 g L^-1^) with constant aeration. They were identified using a pit-tag reader, measured (± 0.1 cm), and weighed (± 0.1 g). In November 2009, blood was sampled for each fish (0.3 mL) by caudal puncture using heparinized syringes. Plasma was obtained by centrifugation (5 min, 5000 rpm, 8500g), and then stored at −80° until analysis. Sperm and eggs were collected from sexually mature animals by stripping. At both sampling periods, adipose fin was cut on all anesthetized fish for genotyping of markers. Fins were individually stored in ethanol (> 95%) until DNA extraction. Anesthetized fish were killed by decapitation according to regulations of Canadian Council of Animal Protection recommendations and protocols approved by the University Animal Care Committee.

Seven phenotypic traits were measured in November 2009 for males and females sampled that were successfully genotyped (n = 86; as discussed in the section *SNP detection*, *validation*, *genotyping*, *and functional characterization*). Plasma sexual steroids (testosterone in males and females; 17β-estradiol in females; 11-ketotestosterone in males) were measured using commercial kits validated in fish [Testosterone ^125^I RIA kit (De Montgolfier *et al.* 2009) and 17β-estradiol ^125^I RIA kit Ria Kit (Davis *et al.* 2009), Immuchem Inc, Biomedicals, OH; 11-keto testosterone EIA kit (de Montgolfier *et al.* 2009), Cayman Chemical Compagny, MI]. The diameter of 10 eggs per female (38 females mature on a total of 40%–95%) was measured using a caliper (± 0.1 mm) under binocular microscope. Sperm from sexually mature males (42 sexually mature males on a total of 46, *i.e.*, 91.3%) was diluted (1:2 × 10^5^) in NaCl 0.9%. Sperm counts and mean spermatozoid head diameter (µm) were measured in triplicates for each male using a Z Series Coulter Counter Cell and Particle Counter (Beckman Coulter Canada Inc., ON, Canada).

### Phenotype data analyses

Data normality was checked for each trait using a Shapiro-Wilk test. Phenotypic traits that were not normally distributed were Log_10_ transformed. The relationships among phenotypes were tested using a correlation matrix Spearman Rho test on both males and females data after removing of outliers (n = 6 and n = 4 for males and females, respectively) using the scatterplot box-plot function of the Statistica software (StatSoft Inc., Tulsa, OK). Statistical significance was considered when *P* < 0.05. All statistical tests were performed using [R] (v. 2.10.1; The R Foundation for Statistical Computing , 2009, 3-900051-07-0), unless specified otherwise.

### Genetic linkage map construction

Before performing linkage mapping, deviation from Hardy-Weinberg Equilibrium (HWE) was tested for every marker using a Pearson’s χ^2^ test implemented in the SNPassoc [R] package (v.1.6.0) (Gonzalez *et al.* 2007). Markers that deviated from HWE equilibrium (*P* < 0.05) were removed from subsequent linkage analyses. Remaining SNP and microsatellite genotype data were formatted in the Crimap input format using a homemade python script (available upon request), and linkage between markers was evaluated using the ‘two points’ option implemented in Crimap. A decreasing LOD (Log_10_ of the odd ratio) score approach (from 6 to 3, step = 1) was used to reveal the most significant linkages (LOD = 6) to the weakest linkages (LOD = 3) and was used to group linked markers in LGs. This allowed mapping markers showing pseudo-linkage to different LG without any misassociation to another LG. Finally, markers that failed to be assigned with confidence to only one LG were not considered in further analysis. Chromosomal distances were evaluated according to the Kosambi formula (in cM) and estimated using the “build” option. Most probable order of marker inside each LG was estimated using the “flipsn” option. Double recombination events and genotyping errors were revealed using the “chrompic” option. Three linkage maps were built: two sex-specific maps and one consensus map combining information from both sexes. LGs were visualized using Mapchart (http://www.joinmap.nl). The linkage map was compared with the brook charr map recently published by Timusk *et al.* (2011) that comprises 37 LGs built with 139 microsatellites genotyped in three F_2_ families. Shared markers between both studies were used to assess homology between linkage groups. Maps were also then compared in terms of length, intermarker distance, and recombination differences between sexes.

### QTL detection

QTL analysis was carried out using the [R] package R/qtl [v. 1.18-7, August 2010, http://www.rqtl.org/ (Broman *et al.* 2003)] on the sex-specific linkage maps. (1) A single QTL analysis was performed using the Haley-Knott regression method (10,000 permutations) to reveal which LGs were carrying QTL. The most probable position of the QTL was defined at the position giving the largest LOD score and this QTL was fixed. (2) A two QTL model based on the Haley-Knott regression was used to refine the QTL detection across the genome with a resolution of 5 cM and eventually to detect two QTL on a single LG linked to a particular trait. (3) The best model fitting our data was used to compute the percent variance explained (PVE expressed in %) by the QTL. The chromosome-wide and the genome-wide thresholds were calculated for each LG using 10,000 permutations. The 1.5 LOD confidence intervals were determined for all analyses following the Bayesian method implemented in the “bayesint” function in R/qtl. The bayesint function calculates an approximate interval (end points around the maximum LOD) for a given chromosome using the genome scan output. Allele effects were determined using the effect plot function in R/qtl with the QTL peak marker or marker nearest to the peak as the reference marker. Additive effects were estimated as half of the differences between two homozygous genotypic values whereas dominance effects were estimated as the deviation of the heterozygous from the average value between two homozygous genotypic values (Lynch and Walsh 1998).

## Results

### 454 data assembly

A total of 388,395 pyroreads with a median length of 237 bp, totaling ∼123 Mb, were obtained from sequencing the cDNA library. A total of 61.2% of these reads were assembled into 6317 contigs of minimal, mean, and maximal sizes of 200, 423, and 2250 bp respectively, with N50 contig size estimated at 673 bp.

### SNP detection, validation, genotyping, and functional characterization

A total of 4841 putative SNPs was detected in 1682 contigs (2.87 SNPs/contig) with an average, minimal, and maximal coverage of 18.2, 10, and 411×. Proportions of transitions were A/G – 27.9% and C/T – 28.2% and those of transversions were A/C – 12.9%, G/T –12.3%, A/T –, 12.1% and C/G – 6.6%. Among these 4841 putative SNPs, 1134 were selected with the objective of validating a subset of 300 markers. Amplicon of expected size was obtained for 46% (522/1134) of these, and Sanger sequencing revealed that 42.1% (220/522) of the amplified markers were false positive (no polymorphism). On the remaining 302 markers, 96.3% (291/302) met the technical requirements of the iPlex Gold assays, and 280 of these were successfully multiplexed in nine panels. Each panel contained 28 to 32 SNP markers (Table S2).

Genotype data were obtained for 171 (n = 85 fish sampled in August and n = 86 fish sampled in November) of the 191 F_2_ progenies (89.5%), and on 96.4% (270/280) of the markers as 10 of these failed to amplify or had >50% of missing data. The overall percentage of missing data for the SNP genotyping was 2.2%. Among the 270 validated SNPs, 45.5% (123/270) exhibited a significant blast hit (*e*-value < 1×10^−40^), among which, ORF predictor identified 118 ORFs (95.9%; 118/123). Within these 118 ORFs, 23 ORFs (19.5%; 23/118) showed a frame shift that was introduced during the assembly of raw data into contigs. These 23 ORFs were ignoredm and the SNP characterization was performed on the 95 remaining ORFs. Among these 95 ORFs, 92 SNPs (96.8%) were assigned to coding regions. Finally, 35 loci (38.0% - 35/92) were characterized as nonsynonymous mutations and 57 loci (61.9%; 57/92) as synonymous mutations (details in Table S3).

### Microsatellite genotyping

The preliminary genotyping tests performed on a subset of eight individuals (the two mapping F_1_ parents and six F_2_ progenies) revealed that 84 markers were polymorphic in at least one of the two F_1_ parents allowing their mapping. Markers that showed complex banding pattern or that failed to amplify in more than 80% of the progeny were removed. The missing percentage of data (missing genotypes) was estimated at 6.4%.

### Linkage map

Seventeen markers (three microsatellites and 14 SNP) were removed because they heavily deviated from the HWE. Thus, the dataset used to build the linkage maps included 81 microsatellites and 256 SNPs, for a total of 337 markers. Six markers (1.8%; 6/337) remained unlinked, and a total of 40 LGs was generated. The consensus (sex-averaged) map contained 266 markers (187 SNPs and 79 microsatellites) distributed among the 40 LGs (see Table 1 for details). The LG length ranged from 1.4 to 132 cM, for a total consensus map length of 2047.5 cM. The average marker spacing per LG ranged from 0.7 to 21.3 cM and was estimated at 8.3 cM over the whole genome. The size of each LG was correlated to the number of markers within each LG (R^2^ = 0.516, *P* < 0.001), as well as to the average spacing between two markers within the LG (R^2^ = 0.182, *P* < 0.01). The recombination rate was approximately 2.19-fold greater in females than in males. The exact position and order of the 266 markers among the 40 LGs are given in Table S5. The sex-specific linkage maps were very similar to the consensus linkage map and included almost the same number of markers (n = 263 in females and n = 261 in males). Markers were generally ordered similarly in the two maps except for eight LGs (LG 2, 6, 10, 14, 17, 24, 31, and 38) when compared to the consensus one. For each sex-specific map, a total of 40 LGs was also obtained.

**Table 1:** Description of the 40 LGs with the number and size of markers composing each LG (in cM) obtained for the consensus (sex-average) linkage map in brook charr

LG	Size of the LG, in cM	No. Markers in the LG	No. SNP	No. SSR	Average Spacing, in cM
LG1	132.2	16	11	5	8.26
LG2	130.2	16	11	5	8.14
LG3	95.7	15	10	5	6.38
LG4	73.2	8	6	2	9.15
LG5	95.2	11	6	5	8.65
LG6	77.5	11	9	2	7.04
LG7	46.6	9	6	3	5.17
LG8	81.2	11	8	3	7.38
LG9	82.3	10	9	1	8.23
LG10	63.6	9	8	1	7.06
LG11	70.1	8	4	4	8.76
LG12	78.4	6	4	2	13.06
LG13	74	7	5	2	10.57
LG14	68.5	6	4	2	11.41
LG15	52.6	6	5	1	8.76
LG16	42.8	4	1	3	10.70
LG17	30.3	6	4	2	5.05
LG18	24.9	5	2	3	4.98
LG19	61.1	4	3	1	15.27
LG20	67.2	5	3	2	13.44
LG21	60.7	6	5	1	10.11
LG22	45.8	5	2	3	9.16
LG23	29.2	4	3	1	7.30
LG24	49.3	5	4	1	9.86
LG25	29.8	4	4	0	7.45
LG26	11.5	3	2	1	3.83
LG27	27.8	3	2	1	9.26
LG28	3.9	4	4	0	0.97
LG29	42.3	2	1	1	21.15
LG30	63.9	3	2	1	21.30
LG31	72.3	17	13	4	4.25
LG32	59.1	3	2	1	19.70
LG33	1.4	2	2	0	0.70
LG34	41.8	3	3	0	13.93
LG35	2.4	3	3	0	0.80
LG36	29.1	3	2	1	9.70
LG37	6.5	3	3	0	2.16
LG38	12.9	11	5	6	1.17
LG39	4.5	5	5	0	0.90
LG40	5.6	4	4	0	1.40
Max	132.25	17	13	6	21.3
Min	1.40	2	1	0	0.70
Average	51.18	6.65	4.75	1.90	8.31
Total	2047.45	266.00	190.00	76.00	-

LG, linkage group; SNP, single nucleotide-polymorphism; SSR, simple sequence repeat.

### Phenotyping and QTL identification

The detailed description of the seven phenotypic traits measured is reported in Table 2. In brief, mean egg diameter (± D) was 3.96 ± 0.173 mm, mean spermatozoid head diameter was 2.86 ± 0.05 µm, and mean sperm concentration was 12.20 ± 3.20 10^9^ mL^-1^. Plasma testosterone was about 1.4-fold greater in males than in females at the time of sampling (20.23 ± 14.46 and 14.49 ± 13.69 ng mL^-1^ respectively). Mean plasma 11-ketotestosterone and 17β-estradiol were 76.85 ± 167.84 and 18.35 ng mL^-1^ respectively.

**Table 2:** Descriptive statistics of phenotypic traits measured in male (M) and female (F) brook charr from the QTL population (F_2_ progeny)

	Spermatozoid Head Diameter, µm	Sperm Concentration, ×10^9^ spermatozoids mL^-1^	Plasma Testosterone, ng mL^-1^	Plasma 11-Keto-testosterone, ng mL^-1^	Egg Diameter, mm	Plasma Testosterone, ng mL^-1^	Plasma 17β-Estradiol, ng mL^-1^
Sex	M	M	M	M	F	F	F
N	42	42	42	35	38	37	38
Min	2.57	4.52	0.56	0.39	3.35	0.85	0.15
Max	2.92	20.12	56.42	674.14	4.30	68.89	85.93
Mean ± SD.	2.86 ± 0.05	12.20 ± 3.20	20.23 ± 14.46	76.85 ± 167.84	3.96 ± 0.17	14.49 ± 13.69	6.91 ± 18.36

N, number of observations, Min, minimum mean value obtained; Max, maximal mean value obtained, mean ± SD, mean ± SD of the mean.

In the female progeny, three significant QTL were detected at the chromosome wide level: two were detected for the egg diameter in LG 11 and LG 34, and one QTL was detected for the plasma 17β-estradiol in LG 8 (Table 3). For each of these traits, total PVE of the QTL detected was estimated at 26.7% (10.6% and 16.1% for each QTL) and 27.6% for egg diameter and plasma 17β-estradiol, respectively. The most probable positions of these QTL, their respective 95% CI, the closest linked molecular markers (one per QTL) as well as additive and dominance effects are presented in Table 3.

**Table 3:** Descriptive statistics, including the LOD score, the position, 95% CI, PVE (%), the associated *P*-value, and the specific additive, and dominance effects of each QTL linked to every phenotype related to reproductive traits

Phenotype	LG	Position(cM)	95% CI (cM)	LOD	PVE, %	*F* Value	*P* Value (F)	Additive Effect	SE	Dominance Effect	SE	Nearest Marker
Egg diameter	11	0.0	0 - 11	5.193	10.62	4.432	0.044	−0.8930	0.0936	−0.8030	0.1345	BX-870052i
	34	0.0	0 - 34	3.824	16.16	6.179	0.043	0.3635	0.0491	0.4824	0.3713	BX319411i
17β-estradiol	8	0.0	0 - 7	3.152	27.62	5.412	0.049	0.0360	0.0056	0.9562	0.1254	sf000754_AC
Sperm concentration	6	21	2 - 48	3.831	18.58	5.543	0.052	0.0257	0.0061	0.3895	0.0327	sf000891_02AC
Spermatozoid head diameter	16	42.9	37 - 42.9	5.290	14.95	9.146	0.029	−1.2260	0.0333	−0.4490	0.0190	SFO-D91

Additive and dominance effect give insight in the mode of action of the genes underlying the variance of the traits. Moreover, direction (−/+) of the dominance or additive effects indicates which parent contributes to decrease the trait the phenotypic values in the studied trait. LOD, Log_10_ of the odd ratio; 95% CI, 95% confidence interval; PVE, percent variance explained; QTL, quantitative trait loci; SE, standard error.

**Figure 1:**
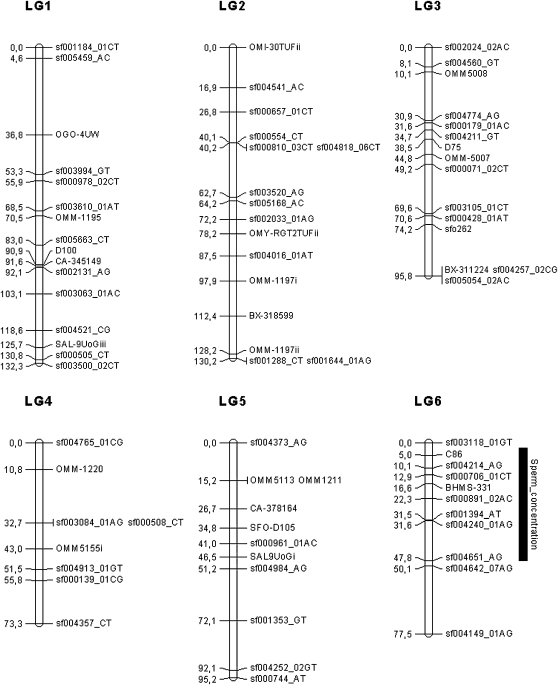

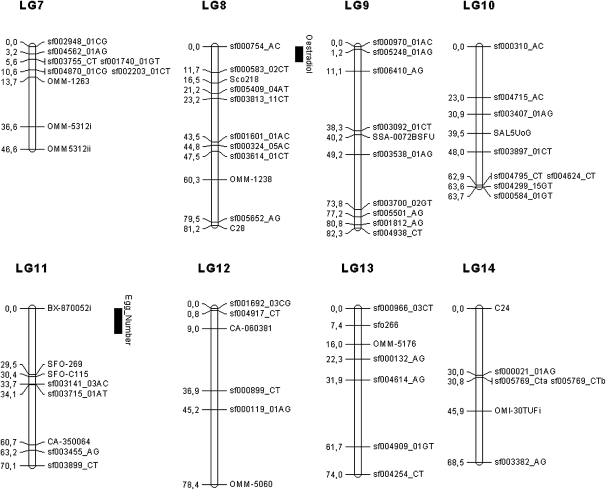

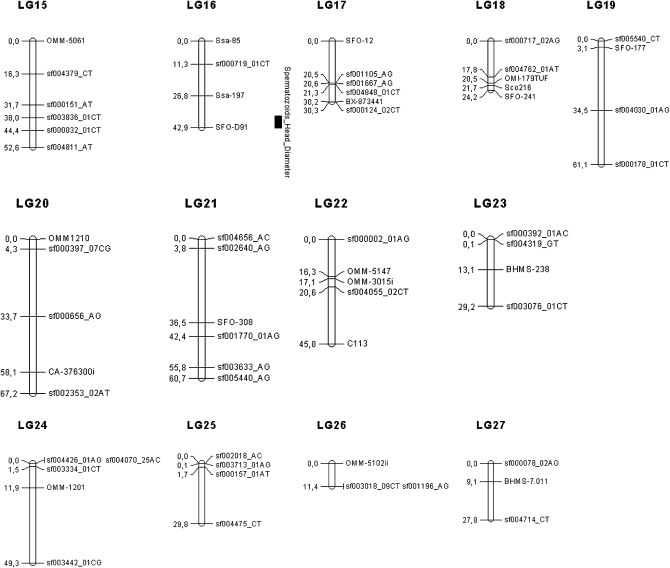

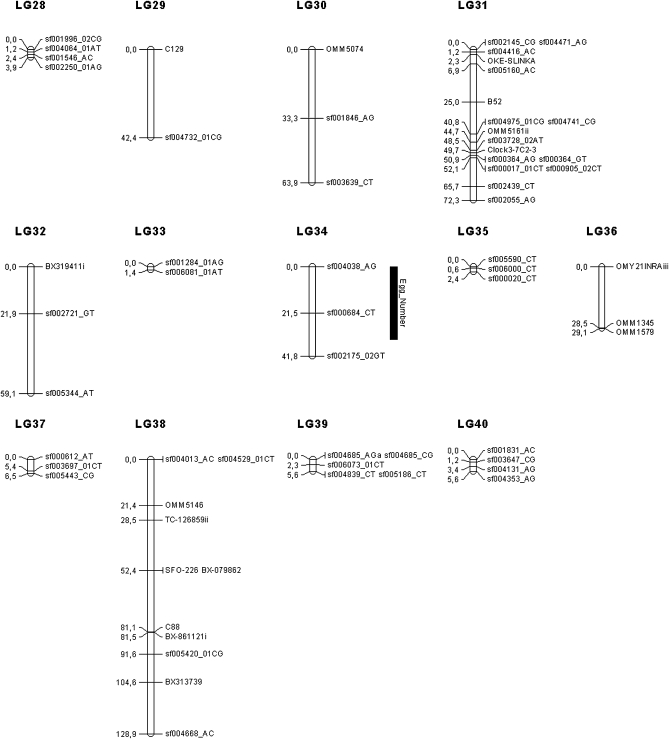
Linkage map in the brook charr: graphical representation of the consensus linkage map (sex-average) built using 266 markers (187 SNPs and 79 microsatellites), on the basis of 40 LGs, spanning 2047.5 cM. Significant QTL associated with reproduction traits are represented with their 95% confidence interval. LGs were named randomly. Genetic distances are indicated on the left of the LG, whereas marker names are reported on the right.

In the male progeny, two QTL were detected, at the chromosome wide level, one for the sperm concentration on LG 6 and one for the average spermatozoid head diameter on LG 16. The PVE associated with these two traits were estimated at 18.58% and 14.95%, respectively. The most probable positions (in cM) of the QTL, their respective 95% CI, and additive and dominance effects are presented in Table 4. All significant QTL regions are depicted in Figure 1. No significant QTL were detected for plasma testosterone neither in male nor in female progeny, neither for the 11-keto-testosterone traits in the male progeny.

**Table 4:** Comparison of putative LGs in *salvelinus* (in cM) between the present study and Timusk *et al.* (2011); homologies with the rainbow trout LGs also are given

LG from Timusk *et al.* (2011)	LG from Present Study	No. of Shared Markers	Markers	Putative Homology with Rainbow Trout LG*^a^*
BC-1	LG24	1	OMM1201	RT-2p; RT-12q; RT-16q; RT-21q; RT-29p; RT-29c
BC-5	LG18	1	OMI179TUF	RT-8p; RT-19c; RT-19q
BC-6	LG5	2	CA378164, OMM1211	RT-3c; RT-3q; RT-6p
BC-8	LG15	1	OMM-5061	RT-2p; RT-3q; RT-7q; RT-10q
BC-9	LG9	1	SSA0072BSFU	RT-14q; RT-19q
BC-10	LG14	1	OMI30TUF/i	RT-10q; RT-18
BC-11	LG29	1	BHMS7.011	RT-10p; RT-12c; RT-12q; RT-19c
BC-13a and BC-13b	LG7	2	OMM5312/i, OMM5312/ii	RT-8c; RT-14p; RT-14c; RT-20p; RT-20q; RT-21q; RT-22p; RT-24p; RT-24c
BC-15	LG2	4	OMI30TUF/ii, OMM1197/i, OMM1197/ii, OMYRGT2TUF/ii	RT-10p; RT-10c; RT-10q; RT-18
BC-19	LG11	1	CA350064	RT-13; RT-22c; RT-23q; RT-27q
BC-20 & BC-43	LG3	2	BX311224, OMM5007	RT-2q; RT-5q; RT-8c; RT-9p; RT-9q; RT-12p; RT-16p; RT-20p; RT-31c; RT-31q
BC-23	LG17	1	BX873441	RT-3q; RT-9p; RT-9q; RT-20p
BC-25	UNA	1	SalD39SFU	RT-6c; RT-6q; RT-11; RT-12p; RT-16c; RT-26
BC-30	LG22	2	OMM3015/i, OMM5147	RT-3p; RT-6p
BC-32	LG13	1	OMM5176	RT-13; RT-23p; RT-23q
BC-34	LG34	1	BX319411/i	RT-14p; RT-14c; RT-20q
BC-36	LG10	1	SAL5UoG	RT-5p; RT-5c

a Based on the Oxford grid of Timusk *et al.* (2011) presented in Table S9. LG, linkage group.

In both sexes, the 95% CI surrounding the most probable QTL positions were highly variable and ranged from 5.9 cM for the QTL linked to the spermatozoid head diameter (LG 16) to 46 cM for the QTL linked to the sperm concentration (LG 6). Two of the five QTL detected (on LG 8 and 6) were linked to the SNP sf000754_AC and sf000891_2AC. These markers were both transversions (A/C), but no significant annotation (or gene name) was found for them. The three remaining QTL (LG 11 and LG 34 for egg diameter and LG 16 for spermatozoid head diameter) were linked to SSR markers (BX870052i, BX319411i, and S*fo*D91).

The five QTL identified for the four phenotypic traits displayed different and significant additive and dominance effects. Additive effects ranged from −1.226 ± 0.033 (QTL on LG 16 associated with spermatozoid head diameter) to 0.363 ± 0.049 (QTL on LG 34 associated with egg diameter) and dominance effects ranged from −0.803 ± 0.134 (QTL on LG 11 associated with egg diameter) to 0.956 ± 0.125 (QTL on LG 8 associated with plasma 17β-estradiol concentration).

## Discussion

The main objective of this study was to identify QTL associated with reproductive physiological traits that have not or rarely been investigated in previous studies. To this end, we first developed new SNP markers specific to brook charr in coding gene regions and used them in combination with previously published microsatellite markers to build a linkage map. We also phenotyped seven physiological traits for which we searched for QTL association. Thus, for the first time in salmonids, five significant QTL for four reproductive physiological traits were detected, including for egg number, plasma 17β-estradiol in female and sperm concentration, and spermatozoid head diameter in males. Results suggest that these four traits may be under the control of one major gene or a small number of genes, explaining an important variance of these traits. Moreover, results also indicate that genes underlying the phenotypic variance of these traits are under different mode of action (additive *vs.* dominance) and may be used to predict an increase or a decrease in their phenotypic values in subsequent generations of selective breeding. Admittedly, as for any gene mapping study, increasing the average marker density to detect QTL with a greater accuracy and thus dissecting the genetic architecture of the traits of interest may improve the results of this first-generation linkage map. Finally, this newly developed panel of mapped SNP located in coding gene region will be useful for screening wild populations, especially in the context of investigating the genetic impact of massive stocking of domestic brook charr to support the angling industry throughout eastern North America.

### SNP development

Using RNA-seq, a total of 280 SNP markers located in coding regions and annotated for about half of them were identified for the first time in the genus *Salvelinus*. High-throughput sequencing technologies are increasingly applied for numerous purposes, including developing SNP markers for building linkage maps (*e.g.*, rainbow trout) (Sanchez *et al.* 2009). To this end, a major constraint when working on nonmodel species is that, although *de novo* assembly approaches are routinely used (Kumar and Blaxter 2010), the absence of a reference genome makes discerning paralogous genes very difficult. This is particularly challenging in species with complex duplicated genomes also comprising numerous repeated elements such as salmonids (Davidson *et al.* 2010). To minimize the orthologous/paralogous problem in this study, each putative SNP was resequenced using the Sanger method. This stringent approach (261 SNPs validated >1000 putative sites) was necessary to overcome uncertainties associated with contig assembly. The low validation rate is likely a consequence of the following factors: (1) the greater sequencing error rate of the 454 technology compared to Sanger sequencing; (2) the assembly of paralogous sequences (even if we used restrictive parameters in the assembly); (3) the design of primers over an exon/intron junction for the validation step leading to the amplification of intronic regions, and (4) the technical requirements for the Sequenom assay. Low SNP validation rate similar to what we obtained here has been previously reported in other salmonids, including chum salmon, *O. keta* (validation success rate of 13.3%; 37/202, Seeb *et al.* 2011), and rainbow trout (validation success rate of 28.9%; 139/480 for Abadia-Cardoso *et al.* 2011 and 48%; 183/384 of validation success for Sanchez *et al.* 2009). Clearly, such a low validation rate is a major constraint to the efficient development and application of SNP markers using the method we used here, and alternative approaches may be better suited in future steps toward densifying the brook charr genetic map.

Indeed, during the time course of this study, new methods allowing the genotyping of thousands of SNP directly from next-generation sequencing sequences have been developed and recently applied (Elshire *et al.* 2011, also reviewed in Davey *et al.* 2011). However, the application of such “genotype by sequencing” methods for genetic mapping has been limited to very few species thus far (*e.g.*, Baxter *et al.* 2011; Chutimanitsakun *et al.* 2011). As the first application to genetic mapping in fishes, the so-called “RAD-sequencing” approach has been used to build a linkage map comprising 8400 SNP markers in the longnose gar (*Lepisosteus osseus*) (Amores *et al.* 2011). Also, Miller *et al.* (2012) have recently produced the first genetic map for salmonids (rainbow trout) using this same method. One potential drawback with these new methods as currently applied is that, unlike the approach we used that specifically targeted coding regions, most detected SNPs are located in noncoding regions and thus remain anonymous, although new protocols are being adapted for cDNA RAD-sequencing (Davey *et al.* 2011). Also, the challenge posed by the recently duplicated genome of polyploidy salmonids remains the same, although the number of markers from which high-quality ones can be validated is substantially increased. Thus, there is little doubt that future development in brook charr genetic mapping and QTL detection will benefit from these new methods, as it will likely be the case for any other species.

### QTL detection for reproductive physiological traits

Up to now, QTL analyses linked to reproductive traits in salmonids mainly focused on age at sexual maturation (*e.g.* Moghadam *et al.* 2007). Here, five significant QTL were detected for other traits related to reproduction, including two QTL associated with egg number, as well as with plasma 17β-estradiol and spermatozoid head diameter. The 17β-estradiol is synthesized from the aromatization of testosterone in the ovarian granulosa cells (Kagawa *et al.* 1983). It is subsequently released into the bloodstream and is responsible for growth and maturation of oocytes (Peter and Yu 1997). In particular, it triggers hepatic synthesis of vitellogenin, the yolk precursor protein (Patino and Sullivan 2002). For each of the five QTL, the closet underlying molecular marker has been identified. Three of them are microsatellites (BX870052i, BX319411i, and S*fo*D91) and two are SNP markers (sf000754 and sf000891) associated with plasma 17β-estradiol and sperm concentration. Unfortunately, these two SNPs were not associated with any significant annotation. Clearly, the availability of a fully sequenced and annotated genome in other salmonids such as Atlantic salmon (Davidson *et al.* 2010) and rainbow trout (Palti *et al.* 2011) will be of paramount importance to refine the annotation of the SNP markers developed here and to investigate the potential functional role of SNP underlying QTL in salmonid species.

The PVE for identified QTL ranged from moderate to large effects (10.6%–27.6%). The low number of QTL identified per trait, combined to the high PVE may suggest that the expression of these traits is under the control of one major or a limited number of genes. However, this should be interpreted cautiously because the limited number of QTL may also reflect a lack of power because of the limited number of progeny and markers that could be analyzed here. Namely, the limited number of progeny may influence QTL detection experiments by underestimating the QTL number and overestimating their respective effects (Beavis 1998; Xu 2003). Also, most likely because of the limited of markers, the most probable location of the five QTL was identified with relatively large 95% confidence intervals.

### Comparison with other linkage maps

Our brook charr linkage map comprises 40 LGs, which is very close to the haploid number of chromosome in the species (2n = 84). With 171 genotyped individuals, and based on the linkage map previously published for this species (Timusk *et al.* 2011), we expected a maximal mapping resolution of 0.58 cM (100/171) and an average marker spacing ranging from 8 to 10 cM (total length of the map estimated from 2400 cM to 3000 cM /300 markers) This expectation was reached with a marker spacing average per LG that ranged from 0.7 to 21.3 cM and a marker spacing average estimated at 8.3 cM over the whole genome. The marker spacing also reached the minimal expectation estimated at a spacing average of 10 cM over the whole genome that is needed for an accurate QTL detection (Darvasi *et al.* 1993).

As mentioned previously, the present map is composed of 40 LGs, including a total of 266 markers (both SNP and SSR), whereas Timusk *et al.* (2011) reported a map composed of 37 evident LGs determined by the mapping of 139 SSR loci. In both studies also, significant differences in recombination rates between sexes were identified, resulting in a female/male ratio of 2.19:1 in this study *vs.* 3.47:1 in Timusk *et al.* (2011). Such strong differential recombination rates between sexes are typical of all salmonid species investigated thus far. Sakamoto *et al.* (2000) were the first to report this phenomenon in salmonids with a ratio of 3.25:1 ratio observed in rainbow trout. Subsequently, a 1.38:1 female/male ratio was reported in Atlantic salmon, (Lien *et al.* 2011), 6.4:1 in brown trout, (Gharbi *et al.* 2006), and 2.6:1: in Arctic charr (Woram *et al.* 2004).

Such large differences in female/male recombination rates could be explained by the differential sex-specific alignment of chromosomes during meiosis (Lee and Wright 1981). This could also result in segregation distortion (non-Mendelian segregation of markers), which was observed in this study (17 markers comprising 3 SSRs and 14 SNPs) as well as by Timusk *et al.* (2011) (33 loci in 9 LGs). Although the number of markers shared between this study and that of Timusk *et al.* (2011) was relatively modest, comparison between the two maps was possible. Thus, a total of 18 LGs sharing 24 SSR markers was apparently homologous between both studies (Table 4). Also, the comparison between brook charr, Arctic charr, and rainbow trout genetic maps done by Timusk *et al.* (2011) allowed us to assign homology within this triplet of species for the 18 common LGs. However, direct comparison of linkage maps has to be interpreted cautiously, given the low number of shared SSR markers between studies. Further comparison with other available maps that were developed with SNP markers (*e.g.*, Atlantic salmon and zebrafish *Danio rerio*) proved impossible given the very low number of shared markers (<5%) between brook charr and Atlantic salmon as well as the high level of divergence between zebrafish and brook charr. Clearly, further investigation of the synteny within the salmonid family using gene-based markers will be of prime interest while the complete genome sequence of the Atlantic salmon and rainbow trout genome soon become available (Davidson *et al.* 2010; Lien *et al.* 2011).

## Supplementary Material

Supporting Information
