# Supplementary material for: Coding Gene Single Nucleotide Polymorphism Mapping and Quantitative Trait Loci Detection for Physiological Reproductive Traits in Brook Charr, Salvelinus fontinalis
Source: G3 (Bethesda). 2012 Mar 1;2(3):379–92. doi: 10.1534/g3.111.001867 (PMC3291508; doi:10.1534/g3.111.001867)
Supplement: Supporting Information [file supp_2_3_379__index.html]

Supporting Information 

# Coding Gene Single Nucleotide Polymorphism Mapping and Quantitative Trait Loci Detection for Physiological Reproductive Traits in Brook Charr, *Salvelinus fontinalis*

## Supporting Information for Sauvage *et al*, 2012

**Files in this Data Supplement:**

- Supporting Information - Tables S1-S5 and Files S1-S3 (PDF, 375 KB)
- Table S1 - Description of the SSR markers used to build the linkage map in the Brook charr, *Salvelinus fontinalis* (PDF, 134 KB)
- Table S2 - Description of the Sequenom panel used to genotype the SNP markers in the F2 progeny (PDF, 185 KB)
- Table S3 - Description and annotation of the 281 SNP (single nucleotide-polymorphism) markers used to build the linkage map in the Brook charr, *Salvelinus fontinalis* (PDF, 151 KB)
- Table S5 - Description of the consenssus and sex-specific linkage maps build using Crimap in brook charr, *Salvelinus fontinalis* (PDF, 80 KB)
- Table S4 - Sequences of the contig build from the assembly of the 454 raw data (.xlsx, 113 KB)
- File S1 - R script containing all the commands necessary to run the analyses. Comments on commands are included in the file. (.zip, 5 KB)
- File S2 - Input file formatted for R/QTL package gathering all the genotypic and phenotypic information (.xlsx, 324 KB)
- File S3 - Raw genotyping data for SSR and SNP markers used in the present study to build the linkage map. (.txt, 218 KB)
