# Supplementary material for: Coding Gene Single Nucleotide Polymorphism Mapping and Quantitative Trait Loci Detection for Physiological Reproductive Traits in Brook Charr, Salvelinus fontinalis
Source: G3 (Bethesda). 2012 Mar 1;2(3):379–92. doi: 10.1534/g3.111.001867 (PMC3291508; doi:10.1534/g3.111.001867)
Supplement: Supporting Information [file supp_2.3.379_001867SI.pdf]

**Supporting Files**

Files S1-S3 are available for download at http://www.g3journal.org/lookup/suppl/doi:10.1534/g3.111.001867/-/DC1.

File S1: R script containing all the commands necessary to run the analyses. Comments on commands are included in the file.
File S2 : Input file formatted for R/QTL package gathering all the genotypic and phenotypic information.
File S3 : Raw genotyping data for SSR and SNP markers used in the present study to build the linkage map.

**Table S1 Description of the SSR markers used to build the linkage map in the Brook charr, *Salvelinus fontinalis***

| | Marker | Primer F et R | Annealing Tempearature | Accession Number | Associated publication |
|---|---|---|---|---|---|
| 1 | BHMS206 | CCAAATAACTGACAAGTGAG | 54 | AF256680 | Timusk et al. 2011 |
| | | CAGAGGTTGATAATGGGG | | | |
| 2 | BHMS238 | GATAATGCCTGGATGTGC | 54 | AF256682 | Timusk et al. 2011 |
| | | CTAGAGCCGACCCTTTAC | | | |
| 3 | BHMS272 | AGCTTGACAGCAGCTTGG | 54 | AF256690 | Timusk et al. 2011 |
| | | TGCAATGCAGACTGACTG | | | |
| 4 | BHMS331 | CAGCACCAGAACATAACC | 54 | AF256744 | Timusk et al. 2011 |
| | | AGCCATCAACACTCCCTG | | | |
| 5 | BHMS377 | TGGCTACAACAGGGATAC | 54 | AF256707 | Timusk et al. 2011 |
| | | AGTCTCTTACATGGAGGC | | | |
| 6 | BHMS417/i/ii/iii/ | ACATAGACCATGACGCTC | 54 | AF256752 | Timusk et al. 2011 |
| | | TGACACGCTCTCTGATCC | | | |
| 7 | BHMS429 | CCCCTGTCAAACGTCTTC | 54 | AF256719 | Thorsen et al., 2005 |
| | | AGCACACTGGATTCAAGG | | | |
| 8 | BHMS465/i/ii | ACTCATCAACTGAGCCCC | 54 | AF256857 | Timusk et al. 2011 |
| | | GTGATCTGTAGTTTTCCATC | | | |
| 9 | BHMS7.011 | GGGACAGCTAATGGATCG | 54 | AF256834 | Timusk et al. 2011 |
| | | GTTTTAGTAATCGGAGTGTG | | | |
| 10 | BX073647/i/ii | TATGGCTTTCCAAACCAAAG | 54 | BX073647 | Timusk et al. 2011 |
| | | ACAGGGGGTTAACAGTGACA | | | |
| 11 | BX073974 | TGTACGGAAAGATGGGCTCC | 54 | BX073974 | Timusk et al. 2011 |
| | | CTCGAAGTCACCCAAACAGC | | | |
| 12 | BX076085 | AAGACAGGAGATGAAGACACCG | 54 | BX076085 | Timusk et al. 2011 |
| | | ATATGTCGTGGGAAACATGTAGG | | | |
| 13 | BX079862 | TGTGAGAAGAACACGAGAGTTGG | 54 | BX079862 | Timusk et al. 2011 |

| | | | | | |
|---|---|---|---|---|---|
| | | GAATGAGGTGTTAGAACGACTGC | | | |
| 14 | BX087664/i/ii | ATAGCCAGAGGGAAGCCTGC | 54 | BX087664 | Timusk et al. 2011 |
| | | GCATCTCCAGCAGTCATTTGG | | | |
| 15 | BX299451 | CCTGGTCTCCTTCACTTTCA | 54 | BX299451 | |
| | | CTGTGCTTACTGGGCAACTT | | | |
| 16 | BX305863 | TACTGTACAGGATGGGTCTCTGC | 54 | BX305863 | Timusk et al. 2011 |
| | | GGAGTACTACGTGTGAGGATTGG | | | |
| 17 | BX311224 | CCGTGTGAAACCTGCATCC | 54 | BX311224 | Timusk et al. 2011 |
| | | CCTTGGAATGCTTACCCTACC | | | |
| 18 | BX311884/i/ii | TGGACAACTTCAGCAAGGACC | 54 | BX311884 | Timusk et al. 2011 |
| | | TGGTGACAGTTTCTGCTGAACC | | | |
| 19 | BX313739 | CATGGAGTGTAAGGCAGGGC | 54 | BX313739 | Timusk et al. 2011 |
| | | CGACAGATCAGCATCGCTGC | | | |
| 20 | BX318599 | GATTTTCTGACCAGCACCTCC | 54 | BX318599 | Timusk et al. 2011 |
| | | CCAACTGGACCCAGAACAGC | | | |
| 21 | BX319197 | CGTCATCCATGTATGTTTCATGC | 54 | BX319197 | Timusk et al. 2011 |
| | | GACAACTCACACATCCACATGG | | | |
| 22 | BX319411/i/ii | GCTGTGGCCCTCTGTCATACC | 54 | BX319411 | Timusk et al. 2011 |
| | | GACATCAACGTGACACCAGGC | | | |
| 23 | BX861121/i/ii | CCTTCATCAAGGATGCAGCC | 50 | BX861121 | Timusk et al. 2011 |
| | | TATGTTTCCCAGTACATACCGGG | | | |
| 24 | BX870052/i/ii | CAGATGAAATTGATCCAGATGACG | 54 | BX87005 | Timusk et al. 2011 |
| | | GGAAGGGGATGATGTGATTGG | | | |
| 25 | BX873441 | GAAGAGTTCCGGTCCATCGG | 54 | BX873441 | Timusk et al. 2011 |
| | | CGTGCATGTAATTCAGCCTGC | | | |
| 26 | BX881655 | AGAAAGACCTGGCAAGGACC | 54 | BX881655 | Timusk et al. 2011 |
| | | CCTCTGGCACAAACTCCAGC | | | |
| 27 | BX890355/i/ii | ACTGAGAACACTTCAGCCAAGG | 54 | BX890355 | Timusk et al. 2011 |

| | | | | | |
|---|---|---|---|---|---|
| | | ACCCACTAGCTGCTACATTATGG | | | |
| 28 | CA060381 | AGTGGTGAAGTGGGATGGGG | 54 | CA060381 | Rise et al., 2004 |
| | | CCCGATGCTTTCTTCATGG | | | |
| 29 | CA061336 | TGCCATGTTTATTGAAATGCC | 52 | CA061336 | Rise et al., 2004 |
| | | GGATCCAAAGGAGAGACTCCTG | | | |
| 30 | CA344270 | CACACTTCCTGGACCACTTCC | 54 | CA344270 | Rexroad et al., 2003 |
| | | TTCAAACTAGGGATTTCGTTGC | | | |
| 31 | CA345149 | ACCACCTCAGAGTGTCTTTCTCC | 54 | CA345149 | Rexroad et al., 2003 |
| | | GACTTGACATACAAACGACGTGG | | | |
| 32 | CA350064 | GACCTGGTTTCGGTGTCAGAGC | 54 | CA350064 | Rexroad et al., 2003 |
| | | CCACTGTGAAGTTAGGTGTTCCC | | | |
| 33 | CA368462/i/ii | CGACAGACTCAGACCACTGTCC | 56 | CA368462 | Rexroad et al., 2003 |
| | | CGACAGACTCAGACCACTGTCC | | | |
| 34 | CA376300/i/ii | TTAAGCACTAAGGGGAAGACGG | 54 | CA376300 | Rexroad et al., 2003 |
| | | TGGGGTGGAAGCAAAGAGC | | | |
| 35 | CA378164 | GAACGGGGTGTCGTAGATGG | 54 | CA378164 | Rexroad et al., 2003 |
| | | CCCTGACCTGTCCTCTTTGG | | | |
| 36 | CL4778 | GAGGATACTGCCATTCAACA | 54 | NA | |
| | | ACGGTCCCACCTTACCATAAA | | | |
| 37 | Clock3-7C2-3 | GAGTACTGCCCTGCAGGTTG | 54 | NA | Timusk et al. 2011 |
| | | TTGACCATGGCCCTCTTATG | | | |
| 38 | CR363293 | TCCGCAACAAGTACGCTGG | 54 | CR363293 | Timusk et al. 2011 |
| | | TTCTCTTCTGGCAACTTCAGACC | | | |
| 39 | Ogo4UW | GTCGTCACTGGCATCAGCTA | 54 | AF009796 | Olsen et al., 1998 |
| | | GAGTGGAGATGCAGCCAAAG | | | |
| 40 | Omi30TUF/i/ii | AGAAGACGAAGTGGATGCTG | 54 | NA | Timusk et al. 2011 |
| | | GTCACCGTTTCTTTACCTGC | | | |

| | | | | | |
|---|---|---|---|---|---|
| 41 | Omi126TUF | TTAAAGGAAACACACGCATACG | 54 | NA | Timusk et al. 2011 |
| | | TTCACACGACCGTTGGTG | | | |
| 42 | Omi179TUF | TTATCCTAGTGCCGGGTCTG | 54 | NA | Timusk et al. 2011 |
| | | ATGCAGCTTTTCAGTGGCTT | | | |
| 43 | OkeSLINRA | GAAAATAACTATAGACATTGCTGG | 54 | NA | Sakamoto et al., 2000 |
| | | CGTCCTTACACTCCAGAGGG | | | |
| 44 | OMM1195 | GCGAGGTTAGGATACACACAT | 54 | AF469980 | Timusk et al. 2011 |
| | | CTTCAGCCTGGAAACACAA | | | |
| 45 | OMM1197/i/ii | CTAGGAGAACAAGAAGACCATCGC | 54 | AF469982 | Timusk et al. 2011 |
| | | AGGACAGAAAGGAGGTAAAACGG | | | |
| 46 | OMM1201 | CCGGAAAAGCTAGGGAGAG | 54 | AF469986 | |
| | | CCCTTCTGTATCCATTCCGTT | | | |
| 47 | OMM1205 | AAACGGTGCCCTCCTTCCTCTATA | 54 | AF469990 | Timusk et al. 2011 |
| | | CCCAAGCCAATAAAGCCCTTACAT | | | |
| 48 | OMM1210 | CATCAGACAGCACAGAGCAG | 54 | AF469994 | Timusk et al. 2011 |
| | | GGAGGAGCAAGCCTTCTAAC | | | |
| 49 | OMM1211 | ACCCACTCTCCCACTCAGTATT | 54 | AF469995 | Timusk et al. 2011 |
| | | GAAGGAGGCTTGGAAGTGTATC | | | |
| 50 | OMM1220 | CTCTGGGACAGACTTATCAC | 54 | AF470002 | Timusk et al. 2011 |
| | | CTATTGGACGATGCACAC | | | |
| 51 | OMM1228 | CCCTTCCTGTGTGTCGTTGTT | 54 | AF470009 | Timusk et al. 2011 |
| | | CAGGAGTCACTTGGCAGTAGGAG | | | |
| 52 | OMM1237/i/ii | GTCAGAGTCGTGGGTATCAA | 54 | AF470017 | Timusk et al. 2011 |
| | | CAGAGTTTCCACGGTCACT | | | |
| 53 | OMM1238 | CGGAAATACGGAGGCTACTGTTG | 54 | AF470018 | Timusk et al. 2011 |
| | | CTTCTCCCTGGCATCTTTCATCAG | | | |
| 54 | OMM1263/i/ii | CTGCATTCCAATACTCCACAG | 54 | AF470029 | Timusk et al. 2011 |
| | | TGGACGAACACTGGATCAG | | | |

| | | | | | |
|---|---|---|---|---|---|
| 55 | OMM1290 | GCCTCAGCACTGTCTTAA | 54 | AF470050 | Timusk et al. 2011 |
| | | CGGAGGTCCCTAGAGA | | | |
| 56 | OMM1329 | GGGAAGTGTTCACCATTACACAAG | 54 | G73564 | Palti, Y et al., 2002 |
| | | CATCCAGGAACGCACCTTTA | | | |
| 57 | OMM1345 | CCCTGGATTCTCCTGTTAG | 54 | G73576 | Palti, Y et al., 2002 |
| | | ACATAGACACAGCACTCATGG | | | |
| 58 | OMM1372/i/ii | CACTTCATGATGCCGAAAGCAG | 54 | BV005159 | Palti, Y et al., 2002 |
| | | CCCCCATCATGACTCCTTCTAGTT | | | |
| 59 | OMM1445 | CTGCGTTATTGGTAGCTTGTG | 54 | BV079589 | Timusk et al. 2011 |
| | | CCCGGTAATGTAGTTCCTGTC | | | |
| 60 | OMM1459 | GCAGGTATTCAGGTAGGTCAG | 54 | BV079593 | Timusk et al. 2011 |
| | | AATGACCATGGAAAACAACAC | | | |
| 61 | OMM1512 | TTTCAAATCAGCCCAGGTTA | 54 | BV212048 | Coulibaly et al., 2005 |
| | | AGGAAAAGGCAGGATGGTAT | | | |
| 62 | OMM1579 | CTAGGCTCTGTGAATCTGA | 54 | BV212097 | Coulibaly et al., 2005 |
| | | GAAGGAATAAGACTGTCCG | | | |
| 63 | OMM3015/i/ii | ACTCTTGCGCTGGTTGTATG | 54 | BV718488 | Timusk et al. 2011 |
| | | GAAGAGTGTGAAAGTTTGGCTG | | | |
| 64 | OMM3075 | CATTTAATTGAGCTGGCCAC | 54 | BV676508 | Timusk et al. 2011 |
| | | CCAGACAGTTCTGAGCAACC | | | |
| 65 | OMM3095 | CTTCCATTCAGGGTAGAGCAC | 54 | BV676517 | Timusk et al. 2011 |
| | | CCAGGTGTGAAAGGGTTTG | | | |
| 66 | OMM5000/i/ii | AACAGAGCAGTGAGGGGACTGAGA | 54 | CO805106 | Timusk et al. 2011 |
| | | CAAGTGATGTTGGTGCGAGGG | | | |
| 67 | OMM5007 | AGATGCCTGTCGAGTGTTG | 54 | CO805113 | Timusk et al. 2011 |
| | | GAGGAGCATCATTTAGAGACTACA | | | |
| 68 | OMM5008 | CTGTTTCGTTGTCCTCATATCAACC | 54 | CO805114 | Timusk et al. 2011 |
| | | TCCATTATCCAATCAGGAGAGCTCTAT | | | |

| | | | | | |
|---|---|---|---|---|---|
| 69 | OMM5014/i/ii | GGGTCTGAAAGGAGCATGG<br>GGAACCTAACATGACGCAACA | 54 | CO805119 | Timusk et al. 2011 |
| 70 | OMM5018 | GAAGGAACGGAACAGAGTGGTAATCAC<br>TCGGACAGGTAACTGGAACGGAT | 54 | CO805123 | Timusk et al. 2011 |
| 71 | OMM5019 | CATGCTGCCTCTCACCGTTTA<br>AACACACCCAGCATCCAACC | 54 | CO805124 | Timusk et al. 2011 |
| 72 | OMM5056 | TCACCATCACCTTCATCGCCT<br>ACATGCTGCCCTTTGACGGAG | 54 | CA349207 | Rexroad et al., 2003 |
| 73 | OMM5060 | TCTCGGGCCAAACCTTCTTATTGC<br>AGCCACTACATCTCCACGCCCTT | 54 | CA348688 | Rexroad et al., 2003 |
| 74 | OMM5061 | GCGTTGGGAGAGAACAATACC<br>CCCATCACACCAGTTGCC | 54 | CA348688 | Rexroad et al., 2003 |
| 75 | OMM5074 | TCGCTTTGGGTAGAAGTTGCCTTTAAC<br>AACATTAAGAACGAGTGGAATCACGC | 54 | CA348721 | Rexroad et al., 2003 |
| 76 | OMM5091 | GCAGGAAAAACACCCAGATACAA<br>ACACTGGCTGGTGTCGTTACATTA | 54 | CA348850 | Rexroad et al., 2003 |
| 77 | OMM5102/i/ii | ATTCCAAATAACAGGTGCTACTGGTC<br>CTGGTTAACTAGGCAACTGATTGTGTC | 54 | CA348955 | Rexroad et al., 2003 |
| 78 | OMM5113 | TCGGTAACAAGTCCTCTAGACCACA<br>CAGAGACCTAGACTGAGTCATGTCCTG | 54 | CA349018 | Rexroad et al., 2003 |
| 79 | OMM5146 | GACAGATTCATGCAAGCCT<br>CCTCACTACTTGCCAATCA | 54 | BV211874 | Coulibaly et al., 2005 |
| 80 | OMM5147 | CACTGTATGTTCTTACCCTG<br>TATACTGGCTGAGTTCAACC | 54 | BV211875 | Coulibaly et al., 2005 |
| 81 | OMM5155i/ii | GGACAGAACTGCCACTAAGTGTG<br>GAGGAGACAGGGAAGAGCTATTG | 54 | BV211883 | Coulibaly et al., 2005 |
| 82 | OMM5161/i/ii | CAAGTGTCCTTTGAGCAC<br>AGCAACTGCTGACACTCC | 54 | BV211889 | Coulibaly et al., 2005 |

| | | | | | |
|---|---|---|---|---|---|
| 83 | OMM5176 | CCACTTGCTGCTTCTCTACATA | 54 | BV211902 | Coulibaly et al., 2005 |
| | | AAGAACACCTAGCCAATAACCC | | | |
| 84 | OMM5179 | CCCTGTCACATGGATGCT | 54 | BV211905 | Coulibaly et al., 2005 |
| | | GATTTGGCAACCGAACAC | | | |
| 85 | OMM5312/i/ii/iii | ACTGTCAGCAGCAATACACT | 54 | BV21202 | Coulibaly et al., 2005 |
| | | CCCATTTTCTCTTGTCACAC | | | |
| 86 | Omy6DIAS | CCACCAACTTCTTACATGAT | 54 | AF239042 | Timusk et al. 2011 |
| | | CTATGGGGACAGCCGAATAA | | | |
| 87 | Omy21INRA/i/ii/iii | GCATTGGCGTAATGAGAAGG | 54 | NA | Gharbi et al., 2006 |
| | | CTGACGGACATATCAGCCC | | | |
| 88 | OmyRGT2TUF | ATAATGTGTCCCCAGGCAAG | 54 | AB087587 | Sakamoto et al., 2000 |
| | | GAGGATGCGTCTTTGCATCT | | | |
| 89 | OmyRT16TUF | TGGCTGAGTTATACATGGAACG | 54 | NA | Timusk et al. 2011 |
| | | TCAATCAGGAGCAGTTAAAACA | | | |
| 90 | Otsclock1b_44_L_2 | TGCTACTGTGGCAACCTTTG | 54 | NA | |
| | | CCTGAGAGAGATGAGGGAGAGA | | | |
| 91 | Sal5UoG | TTTGCATTGAGCCTCTGTTG | 54 | NA | Timusk et al. 2011 |
| | | TGTTTCAGCTGCTATTAGGAAT | | | |
| 92 | Sal9UoG/i/ii/iii | TCACTGCTTCAAGGTATTTTTACTT | 54 | NA | Timusk et al. 2011 |
| | | AATTAGAGCTGCTAGGTCAGTGAG | | | |
| 93 | SalD25SFU | GATCTACACAGACCCCCACC | 54 | AF537305 | McGowan et al., 2004 |
| | | CCGTTCTTCCAATAACTGCTC | | | |
| 94 | SalD39SFU | GGGGAGTCTGTGTTAAGTTGG | 54 | AF537310 | McGowan et al., 2004 |
| | | TGAATGGACGTTCCTCTGAC | | | |
| 95 | SalE38SFU | CGCCTTGTCATACATTACACC | 54 | AF537309 | McGowan et al., 2004 |
| | | CCGTTCTTCCAATAACTGCTC | | | |
| 96 | SalF41SFU | ATCCGCTATGAACCACAGG | 54 | AF537306 | McGowan et al., 2004 |
| | | ACTGCTCCGGCAACTACAG | | | |

| | | | | | |
|---|---|---|---|---|---|
| 97 | Ssa0017BSFU | CGCAACAGGCTTAGTTAGAT<br>CAACATTCAGTTCCCTTCAT | 54 | AF019154 | Timusk et al. 2011 |
| 98 | Ssa0033BSFU | ATTCTTGATCGTGGTCTTTG<br>CCGTTCTTCCAATAACTGCTC | 54 | NA | Phillips et al., 2009 |
| 99 | Ssa0072BSFU | TCCGAAGATAGGGGAGGTT<br>ATAAACATCTGGGTGGCTGC | 54 | NA | Phillips et al., 2009 |
| 100 | Ssa0080BSFU/i/ii | CTACTGCACAGCACCTGGAA<br>CACAGAGCACCTCCTGAACA | 54 | NA | Phillips et al., 2009 |
| 101 | TC126859i/ii | TTTTCTCCCTTTGACGACAG<br>TGGTTGTGACTCGATGTCTG | 54 | NA | Phillips et al., 2009 |
| 102 | C113 | GGAGCCCAGACTATATTGACG<br>CCTTGAAGTCTTGCCAGAT | 64 | NA | King TL, unpublished |
| 103 | B52 | GCACACGAAACCAGTATATTTC<br>TTGTCTTGGTGATTTCAGAGC | 64 | NA | King TL, unpublished |
| 104 | C28 | CAGTTGAAGTGATTGGGTTAGC<br>TCATCCTTAAAGCAGAATACCAC | 64 | NA | King TL, unpublished |
| 105 | C129 | AGTGGGTACAACATACCTTTGG<br>AGGTATTCACACCTCAGATTGG | 64 | NA | King TL, unpublished |
| 106 | C88 | TAGTCTCTGGTGGGGAATAATG<br>ATATCAGCCATAAGAGCTGGAG | 60 | NA | King TL, unpublished |
| 107 | C24 | GCTACTGTTGGATTTCATCTCAG<br>ATCACAGAGATGGGGTGATG | 60 | NA | King TL, unpublished |
| 108 | D100 | ACCTTTGACCTGTACATCGTG<br>CAGACCTAGACTAAAGCATCCG | 60 | NA | King TL, unpublished |
| 109 | D75 | GTAGTGCCAAAACAGGTAGAGC<br>CATCCTTATTCCAACCTCAATC | 60 | NA | King TL, unpublished |
| 110 | C86 | ACCGATGGCCTTCAACAC<br>ATAGGCCCCTACCTCAAACC | 60 | NA | King TL, unpublished |

| | | | | | |
|---|---|---|---|---|---|
| 111 | Sfo266 | CTGGCAGCATTGTAAAGAAG<br>CTGGGTGATTTGACGACC | 64 | NA | Perry et al., 2005 |
| 112 | Sco216 | CCTTGTGAGAGCTAAGGTAGTG<br>GGAGGACATATTCCAACTTTG | 64 | NA | Dehaan and Ardren 2005 |
| 113 | Sfo262 | CCCATGTCAGTATTGGACTC<br>CTTCATGGGCAGAATGGAC | 64 | NA | Perry et al., 2005 |
| 114 | Sco218 | TTCTAACTGTTGGCACTCTG<br>GTGTGGTTGGGTGGTAAG | 60 | NA | Dehaan and Ardren 2005 |
| 115 | SFO241 | CTCCATTAGAAAGGGTTTG<br>CCAGTCTTTAGTCAACGC | 60 | NA | King TL, unpublished |
| 116 | SFO091 | AAATAACAACAATATGTGAGAAC<br>TATGCTGATATTGACTTTGG | 60 | NA | King TL, unpublished |
| 117 | SFO308 | CAGCAATGGGGCTGAAGTAG<br>GTCACTGTGTGAATCCTCC | 60 | NA | Perry et al., 2005 |
| 118 | SFO269 | GTAGATGAAACCTGATGG<br>GTTCTATGGTCACATACTG | 60 | NA | Perry et al., 2005 |
| 119 | SFOD105 | CAGGGAAAATGCTAATGTGC<br>GGTTGTGTCGAATGGAGTTC | 60 | NA | King TL, unpublished |
| 120 | Ssa85 | AGCTGGGTCCTCCAAGCTAC<br>ACCCGCTCCTCACTTAATC | 60 | NA | King TL, unpublished |
| 121 | SFOC115 | CAGTTTCTATCTCCAGGCAATC<br>TTCTGAAAGCACTCAACATGG | 60 | NA | King TL, unpublished |
| 122 | SSA197 | GGGTTGAGTAGGGAGGCTTG<br>TGGCAGGGATTTGACATAAC | 60 | NA | O'Reilly et al., 1996 |
| 123 | SFO12 | GGTTTTGAAGAGTGACAG<br>CCCGTTTCACAATCAGAG | 60 | NA | Angers et al., 1995 |
| 124 | SFO177 | CGAATGTGGAGCTGAACTG<br>GGGTATTTGTACAATGGGT | 60 | NA | Perry et al., 2005 |

| | | | | | |
|---|---|---|---|---|---|
| 125 | ONE8 | AACATTCTGGGATGACAGGGGTA<br>CTGTTCTGCTCCAGTGAAGTGGA | 60 | NA | Scribner et al., 1996 |
| 126 | SFO226 | GAGGGCTAGAGACTAGCTTCAG<br>GCAGTGGAACAAATACCCAG | 60 | NA | Perry et al., 2005 |


McGowan, CR, Davidson, EA, Woram, RA, Danzmann, RG, Ferguson, MM and Davidson, WS, 2004. Ten polymorphic microsatellite markers from Arctic charr (Salvelinus alpinus): linkage analysis and amplification in other salmonids Anim. Genet. 35:479-481.

Olsen, JB, Bentzen, P, and Seeb, JE, 1998. Characterization of seven microsatellite loci derived from pink salmon. Mol. Ecol. 7: 1087-1089

O'Reilly P, Hamilton L, McConnell S, Wright J (1996). Rapid analysis of genetic variation in Atlantic salmon (Salmo salar) by PCR multiplexing of dinucleotide and tetranucleotide microsatellites. Canadian Journal of Fisheries and Aquatic Sciences 53: 2292-2298.

Phillips, RB, Keatley, KA, Morasch, MR, Ventura, AB Lubieniecki, KP, Koop, BF, Danzmann, RG and Davidson, WS, 2009. Assignment of Atlantic salmon (Salmo salar) linkage groups to specific chromosomes: Conservation of large syntenic blocks corresponding to whole chromosome arms in rainbow trout (Oncorhynchus mykiss) BMC Genetics 10:46

Palti, Y, Fincham, MR and Rexroad, CE, III, 2002. Characterization of 38 polymorphic microsatellite markers for rainbow trout (Oncorhynchus mykiss). Mol. Ecol. Notes. 2: 449-452

Perry GML, King TL, St-Cyr J, Valcourt M, Bernatchez L 2005. Isolation and cross-familial amplification of 41 microsatellites for the brook charr (Salvelinus fontinalis). Molecular Ecology Notes 5(2): 346-351.

Rexroad, CE, III, Lee, Y, Keele, JW, Karamycheva, S, Brown, G, Koop, B, Gahr, SA, Palti, Y, and Quackenbush, J. 2003. Sequence analysis of a rainbow trout cDNA library and creation of a gene index. Cytogenet. Genome Res. 102: 347-354

Rise, ML, von Schalburg, KR, Brown, GD, Mawer, MA, Devlin, RH, Kuipers, N, Busby, M, Beetz-Sargent, M, Alberto, R, Gibbs, AR, Hunt, P, Shukin, R, Zeznik, JA, Nelson, C, Jones, SR, Smailus, DE, Jones, SJ, Schein, JE, Marra, MA, Butterfield, YS, Stott, JM, Ng, SH, Davidson, WS and Koop, BF, 2004. Development and application of a salmonoid EST database and cDNA microarray: data mining and interspecific hybridization characteristics. Genome Res 14:478-490

Sakamoto,T, Danzmann, RG, Gharbi, K, Howard, P, Ozaki, A, Sokkean, K, Woram, RA, Okamoto, N, Ferguson, MM, Holm, L-E, Guyomard, R and Hoyheim, B, 2000. A microsatellite linkage map of rainbow trout (*Oncorhynchus mykiss*) characterized by large sex-specific differences in recombination rates. Genetics 155:1331-1345.

Scribner KT, Gust JR, Fields RL (1996). Isolation and characterization of novel salmon microsatellite loci: cross-species amplification and population genetic applications. Can J Fish Aquat Sci 53: 833-841.

Timusk, E. R., M. Ferguson, H. K. Mogahadam, J. D. Norman, C. Wilson *et al.*, 2011. Genome evolution in the fish family salmonidae: generation of a brook charr genetic map and comparisons among charrs (Arctic charr and brook charr) with rainbow trout. BMC genetics 68:12.

Thorsen, J, Zhu, B, Frengen, E, Osoegawa, K, de Jong, PJ, Koop, BF, Davidson, WS and Hoyheim, B, 2005. A highly redundant BAC library of Atlantic salmon (*Salmo salar*): an important tool for salmon projects. BMC Genomics 6:50

**Table S2 Description of the Sequenom panel used to genotype the SNP markers in the F2 progeny**

| Technology | Panel | Marker Name | Oligo_Forward | Forward_oligo_Sequence | Oligo_Reverse | Reverse_Oligo_Sequence | Oligo_Probe | Probe_Sequence |
|---|---|---|---|---|---|---|---|---|
| sequenom | SEQ010 | sf000032_01CT | sf000032_01CT_GA_F | ACGTTGGATGGGCGTCCCTGTTGAATTATC | sf000032_01CT_GA_R | ACGTTGGATGGGCACACCCTTCTTTATCAC | sf000032_01CT_GA_Pa | CTTTATCACACACACTATTACTG |
| sequenom | SEQ010 | sf000124_02CT | sf000124_02CT_AG_F | ACGTTGGATGGCCCAGTTGCCTATAGATTC | sf000124_02CT_AG_R | ACGTTGGATGGTCTCCCTTATTCCCTTTGC | sf000124_02CT_AG02_P | CCTTATTCCCTTTGCACCAGTTTGTTAG |
| sequenom | SEQ010 | sf000178_01CT | sf000178_01CT_GA_F | ACGTTGGATGCTTGCCACTTTTCCTGGTAG | sf000178_01CT_GA_R | ACGTTGGATGCTTGTCTGGGACAAACTGAG | sf000178_01CT_GA_Pa | GGGACAAACTGAGATACGA |
| sequenom | SEQ010 | sf000191_01AG | sf000191_01AG_GC_F | ACGTTGGATGGACCTCTGTTTTACCGTGAG | sf000191_01AG_GC_R | ACGTTGGATGGGCACTTCTTTGTATTGTGG | sf000191_01AG_GC02_Pa | CCACTTGATAGGCAGCCA |
| sequenom | SEQ010 | sf000228_01AC | sf000228_01AC_TA_F | ACGTTGGATGTACAACAATTGTTCCCACCC | sf000228_01AC_TA_R | ACGTTGGATGGTTCCCACAATCAACCACTG | sf000228_01AC_TA_Pa | TGCTCCAATCACAGGT |
| sequenom | SEQ010 | sf000327_02AC | sf000327_02AC_GA_F | ACGTTGGATGATGAGTGACAGACGAGCTAC | sf000327_02AC_GA_R | ACGTTGGATGTGCCTTGGTAGTCTATTCTC | sf000327_02AC_GA_Pa | AAGACAAAGACTAACAAGTTAATTAC |
| sequenom | SEQ010 | sf000657_01CT | sf000657_01CT_TC_F | ACGTTGGATGTTTCACACACTGTTCGAGGG | sf000657_01CT_TC_R | ACGTTGGATGCGGTTTTGAACCGATGAAGC | sf000657_01CT_TC_Pa | AAAAAATGTTCGAGGGTTTCTATG |
| sequenom | SEQ010 | sf000679_01CT | sf000679_01CT_TC_F | ACGTTGGATGGGATATTTTAGTGACATCGG | sf000679_01CT_TC_R | ACGTTGGATGCCAGTGTGACAAAGGTTATG | sf000679_01CT_TC_Pa | CATTGTAATATGTGTATGTAGGCCTAGTA |
| sequenom | SEQ010 | sf000810_03CT | sf000810_03CT_CT_F | ACGTTGGATGCCTAAACACAGAAACAAGGG | sf000810_03CT_CT_R | ACGTTGGATGTGAAGTATGCAAGGAGGGAG | sf000810_03CT_CT02_P | GGCAAGGAGGGAGTTTATCACAGA |
| sequenom | SEQ010 | sf000878_01AG | sf000878_01AG_CT_F | ACGTTGGATGACGTTAGTTGGGTGCAAGGG | sf000878_01AG_CT_R | ACGTTGGATGAACAAACTAGGCTAGCTCCC | sf000878_01AG_CT02_P | CCCCTAGTTAACTCTGGAAAATAGGA |
| sequenom | SEQ010 | sf000891_02AC | sf000891_02AC_CA_F | ACGTTGGATGCAGAAATCCTACACTCGTCC | sf000891_02AC_CA_R | ACGTTGGATGTCCTTCTTTAAAGAGGCAGG | sf000891_02AC_CA02_Pa | GGAGAGACGGAAGATTC |
| sequenom | SEQ010 | sf000961_01AC | sf000961_01AC_CT_F | ACGTTGGATGCCCCTCAAGGATAGAAGAAC | sf000961_01AC_CT_R | ACGTTGGATGACTGTAATACAGAAACCAGC | sf000961_01AC_CT_P | ACCCAGCTATTCTCTGAATGTACAATT |
| sequenom | SEQ010 | sf000970_01AC | sf000970_01AC_GA_F | ACGTTGGATGAGGAAGCGATGAGTTAGGAC | sf000970_01AC_GA_R | ACGTTGGATGAAATGGCAACTCGGTTGAGG | sf000970_01AC_GA_Pa | TAAACTCGGTTGAGGGAGGGAGATA |
| sequenom | SEQ010 | sf001181_01CT | sf001181_01CT_GA_F | ACGTTGGATGTGTGCAGTGATCCACAGTTC | sf001181_01CT_GA_R | ACGTTGGATGCAAACACACACACAAACCCC | sf001181_01CT_GA_Pa | GTTCTGTTGGAGCATTG |
| sequenom | SEQ010 | sf001661_01AC | sf001661_01AC_GT_F | ACGTTGGATGTCAGAATCCAGGGCTTGAAC | sf001661_01AC_GT_R | ACGTTGGATGGGGTACTTCAGAGCCAAATG | sf001661_01AC_GT02_P | TGTGTTGGGTTCAGAGAAT |
| sequenom | SEQ010 | sf001740_01GT | sf001740_01GT_GA_F | ACGTTGGATGCCAAAGCCCAACAAAAAAGG | sf001740_01GT_GA_R | ACGTTGGATGGAGTTTCCTGAAAGTTCAC | sf001740_01GT_GA_Pa | TGAAAGTTCACATTAGACTCTAG |
| sequenom | SEQ010 | sf002175_02GT | sf002175_02GT_TC_F | ACGTTGGATGTGCCTGACATCTGAACTCTG | sf002175_02GT_TC_R | ACGTTGGATGTTTTCACAGTGGCGGAACGG | sf002175_02GT_TC02_Pa | AGCGGAACGGGCCAATG |
| sequenom | SEQ010 | sf003063_01AC | sf003063_01AC_CA_F | ACGTTGGATGACCCTCAGCCTATCATATCC | sf003063_01AC_CA_R | ACGTTGGATGGCAGTACCTACATTGTGTCC | sf003063_01AC_CA_P | GGTAGCAAATGTTGCTACAGCAACAATGAG |
| sequenom | SEQ010 | sf003084_01AG | sf003084_01AG_AG_F | ACGTTGGATGCCATGGCTCTCTCTATAGAC | sf003084_01AG_AG_R | ACGTTGGATGGCAATATGGGGTAACACAAG | sf003084_01AG_AG_P | CAGCATTATTACTGGTACAATTAATGCATA |
| sequenom | SEQ010 | sf003407_01AG | sf003407_01AG_TC_F | ACGTTGGATGTGCAGGCCATTCCAAAAAGC | sf003407_01AG_TC_R | ACGTTGGATGCTGCCCATTCTGAAGAAACG | sf003407_01AG_TC02_Pa | CCTGAGCATGTTATTACCTGACAAGAA |
| sequenom | SEQ010 | sf003601_01GT | sf003601_01GT_CT_F | ACGTTGGATGGTAAAGTAGCTGGTGCCTTG | sf003601_01GT_CT_R | ACGTTGGATGAGCCCCGGATTTATGTATGC | sf003601_01GT_CT_P | ACAGTAGCAACAAACCCCT |
| sequenom | SEQ010 | sf003621_01AC | sf003621_01AC_TC_F | ACGTTGGATGGAGCCATAGGTAATGTCCAG | sf003621_01AC_TC_R | ACGTTGGATGCATTCCTACCGGAGTGTAAC | sf003621_01AC_TC02_Pa | CCAACTCTGTTTGAGATGTC |
| sequenom | SEQ010 | sf003698_01AT | sf003698_01AT_GT_F | ACGTTGGATGTTGGTTGAAGGGAAAGGCTG | sf003698_01AT_GT_R | ACGTTGGATGAACACAGTGCCTTCTCACTC | sf003698_01AT_GT_Pa | TCTCACTCACCCTAAAACAAAT |
| sequenom | SEQ010 | sf003713_01AG | sf003713_01AG_TA_F | ACGTTGGATGCGATTCAGACACATCTATGG | sf003713_01AG_TA_R | ACGTTGGATGTAGATAGCAGATCTCGGAAG | sf003713_01AG_TA_Pa | CTACTCCACATGTGATGTTAAT |
| sequenom | SEQ010 | sf003724_01CG | sf003724_01CG_TC_F | ACGTTGGATGCTGGCTTTGCTTGTAAACCC | sf003724_01CG_TC_R | ACGTTGGATGGGAAAAATCTGCATGGTTTAC | sf003724_01CG_TC_Pa | CCCCCCATCTGCATGGTTTACCCATTG |

| | | | | | | | | |
|---|---|---|---|---|---|---|---|---|
| sequenom | SEQ010 | sf003897_01CT | sf003897_01CT_GA_F | ACGTTGGATGGGGAATCATAGGCAATGAGG | sf003897_01CT_GA_R | ACGTTGGATGGGCGTAGTCGGTACTTTATG | sf003897_01CT_GA02_Pa | CCATACCTCCAGCTTGACT |
| sequenom | SEQ010 | sf003919_01CT | sf003919_01CT_CT_F | ACGTTGGATGCAGTCCCTTGGTATAATGGC | sf003919_01CT_CT_R | ACGTTGGATGGAATCCTGACCAGAGTAGTG | sf003919_01CT_CT_P | ACTGACCAGAGTAGTGACCCAATT |
| sequenom | SEQ010 | sf004030_01AG | sf004030_01AG_AT_F | ACGTTGGATGGAGGAAAGACAGTGGGAAAG | sf004030_01AG_AT_R | ACGTTGGATGGGCTGGTCTAGCCTATTTAG | sf004030_01AG_AT_P | TGGATTCAAACATGTGAGC |
| sequenom | SEQ010 | sf004070_25AC | sf004070_25AC_GA_F | ACGTTGGATGCACAAATTTCTGGGTCTGGG | sf004070_25AC_GA_R | ACGTTGGATGAGCTGTTAGCAGAAAGCTGG | sf004070_25AC_GA_Pa | ATCAATCAATCACACTCACTT |
| sequenom | SEQ011 | sf000071_02CT | sf000071_02CT_CA_F | ACGTTGGATGCAGATTAGGCCTAGACAACG | sf000071_02CT_CA_R | ACGTTGGATGCACAGGTACTAAGAGTGTGC | sf000071_02CT_CA_Pa | GGACTAAGAGTGTGCTGAAATT |
| sequenom | SEQ011 | sf000078_02AG | sf000078_02AG_AT_F | ACGTTGGATGAGGGAAAGGGTTTTAAGCGG | sf000078_02AG_AT_R | ACGTTGGATGGCCACATTAACATACACCTG | sf000078_02AG_AT_P | GACCTACAGAAGAAAATAAAATAAGC |
| sequenom | SEQ011 | sf000137_01AG | sf000137_01AG_TA_F | ACGTTGGATGTTCGAAACGCAATCACCGAG | sf000137_01AG_TA_R | ACGTTGGATGACTGAATTGACGGGATCAGC | sf000137_01AG_TA_Pa | AGACATGGAGTCCTATACTAT |
| sequenom | SEQ011 | sf000324_05AC | sf000324_05AC_CA_F | ACGTTGGATGTCTGGAAACTGCGTTCTACC | sf000324_05AC_CA_R | ACGTTGGATGAGACAGCGGATTTGAGACAG | sf000324_05AC_CA_P | GTCTCCACACTTTACTTCCT |
| sequenom | SEQ011 | sf000390_01AC | sf000390_01AC_TC_F | ACGTTGGATGCACAGGTATACTGTTGGTCG | sf000390_01AC_TC_R | ACGTTGGATGACCATGGTACTCATGACTGG | sf000390_01AC_TC_Pa | TGGAGGGCTTTGACAT |
| sequenom | SEQ011 | sf000397_07CG | sf000397_07CG_CG_F | ACGTTGGATGTTGCCTGTTTGAGTGTTTGG | sf000397_07CG_CG_R | ACGTTGGATGCAAAGACTAAACAACAGCCC | sf000397_07CG_CG_P | GTTGAGCACCAACCACAA |
| sequenom | SEQ011 | sf000428_01AT | sf000428_01AT_AT_F | ACGTTGGATGACCGTATGTTATGCAGCCAG | sf000428_01AT_AT_R | ACGTTGGATGCTGGACAATCTAAGCACTGG | sf000428_01AT_AT02_P | ACATTAAGCATGGTAGGCTATATAA |
| sequenom | SEQ011 | sf000584_01GT | sf000584_01GT_GA_F | ACGTTGGATGGGGACTCCACACATTACAAA | sf000584_01GT_GA_R | ACGTTGGATGAACTAACAGTTAGCATGGTG | sf000584_01GT_GA_Pa | GAGAAAAGCTGAACAAAGTAATTTTCTA |
| sequenom | SEQ011 | sf000854_01CT | sf000854_01CT_GT_F | ACGTTGGATGCGGTGTCTGCGATGATTTAG | sf000854_01CT_GT_R | ACGTTGGATGTCTCCCTCCTCTCAATCTTC | sf000854_01CT_GT02_P | CACGTCGGCTCAGCATCA |
| sequenom | SEQ011 | sf001086_01CT | sf001086_01CT_TC_F | ACGTTGGATGTACTGTCACGACCAACACTC | sf001086_01CT_TC_R | ACGTTGGATGATTTACACCCCAGGTCTAAC | sf001086_01CT_TC_Pa | TAACCCAGGTCTAACATACCT |
| sequenom | SEQ011 | sf001184_01CT | sf001184_01CT_TC_F | ACGTTGGATGTCTCTCAACAGCAAGAAGGG | sf001184_01CT_TC_R | ACGTTGGATGCACTGCTCAGACGCTCTTTG | sf001184_01CT_TC02_Pa | CCAACACATTACTTGCTCATCAATATT |
| sequenom | SEQ011 | sf001284_01AG | sf001284_01AG_GT_F | ACGTTGGATGGCTATTCCCCAAGTTTCCTC | sf001284_01AG_GT_R | ACGTTGGATGCGGCCGTTCTTTAAATTACC | sf001284_01AG_GT_Pa | CTTTTCCTCAAAGCCACA |
| sequenom | SEQ011 | sf001770_01AG | sf001770_01AG_AG_F | ACGTTGGATGAGGCACTGGTGTCTTTGTTC | sf001770_01AG_AG_R | ACGTTGGATGTCACGCTTAACTAAGGTCGG | sf001770_01AG_AG_P | TAAGGTCGGCCCCTAT |
| sequenom | SEQ011 | sf002024_02AC | sf002024_02AC_AG_F | ACGTTGGATGGCTATCTCTGGATAGAGAGG | sf002024_02AC_AG_R | ACGTTGGATGCCTGTGTGTTTGTACATGCG | sf002024_02AC_AG_P | TTGGCTTCCAGTTATACTACCAC |
| sequenom | SEQ011 | sf002033_01AG | sf002033_01AG_TA_F | ACGTTGGATGAGTTGAGGTTGGAGGAGTAG | sf002033_01AG_TA_R | ACGTTGGATGGAAACTACACCTACTGTCAC | sf002033_01AG_TA_Pa | GGAGTAGTGATTGTCATTGG |
| sequenom | SEQ011 | sf002155_01CT | sf002155_01CT_AG_F | ACGTTGGATGGTACCACCTTGATTGTCACG | sf002155_01CT_AG_R | ACGTTGGATGATGAAGCCTGATATTCCAAC | sf002155_01CT_AG02_P | CTCTTTTTAATCAAACATTCCATTTGCA |
| sequenom | SEQ011 | sf002948_01CG | sf002948_01CG_GT_F | ACGTTGGATGAAGGTCAGAGCAGTGTTTCC | sf002948_01CG_GT_R | ACGTTGGATGATCTGAGCGGTAGATCTAGG | sf002948_01CG_GT02_P | CGACTCAGAAACGTTGGT |
| sequenom | SEQ011 | sf003141_03AC | sf003141_03AC_TA_F | ACGTTGGATGTGTTGGATGCCTTTGTTCGG | sf003141_03AC_TA_R | ACGTTGGATGCTGACAGACTCCACTTTTCC | sf003141_03AC_TA02_Pa | AGTCCACTTTTCCCAGCGCAT |
| sequenom | SEQ011 | sf003197_01AG | sf003197_01AG_GA_F | ACGTTGGATGCAGAGCACCTATGATGTGAC | sf003197_01AG_GA_R | ACGTTGGATGAGCCTCCTTTACGAGTTACC | sf003197_01AG_GA02_Pa | GGGGTACCTCCCCATTGTCCGTCTGATTC |
| sequenom | SEQ011 | sf003589_01AC | sf003589_01AC_CT_F | ACGTTGGATGGCAGGGACACGAGTTATTTG | sf003589_01AC_CT_R | ACGTTGGATGTTCACACTCACCACGTATGC | sf003589_01AC_CT_P | CCTAGTCACTTTAACCCTA |
| sequenom | SEQ011 | sf003610_01AT | sf003610_01AT_AT_F | ACGTTGGATGTAGCTGGGCGTGCTTAACTG | sf003610_01AT_AT_R | ACGTTGGATGGACTCTGAGAACGTATACCG | sf003610_01AT_AT_P | GGGTATACCGTAAACCCATTCCCCCC |
| sequenom | SEQ011 | sf003700_02GT | sf003700_02GT_TC_F | ACGTTGGATGGCGGTGACTATACTGTTGTG | sf003700_02GT_TC_R | ACGTTGGATGTACAATCGACCGAGAGCATC | sf003700_02GT_TC_Pa | AGACCGAGAGCATCTCATTTATAC |
| sequenom | SEQ011 | sf003728_02AT | sf003728_02AT_TC_F | ACGTTGGATGAACCAGCCTATCAAGTGGTC | sf003728_02AT_TC_R | ACGTTGGATGACAACAGGAACAGCATCCAG | sf003728_02AT_TC02_Pa | ACAGCATCCAGACTGAACATAG |
| sequenom | SEQ011 | sf003836_01CT | sf003836_01CT_TA_F | ACGTTGGATGGAGGTTTCTACTGTCACCTG | sf003836_01CT_TA_R | ACGTTGGATGCGACTGCAACTGAAAGCAAC | sf003836_01CT_TA_Pa | AAACTGAAAGCAACATGGGCATGC |

| | | | | | | | | |
|---|---|---|---|---|---|---|---|---|
| sequenom | SEQ011 | sf004064_01AT | sf004064_01AT_CA_F | ACGTTGGATGGAACACAGTGGTCTCCATAG | sf004064_01AT_CA_R | ACGTTGGATGCACATGCCACCCAATATGAG | sf004064_01AT_CA02_Pa | AGTATAATAAACTAGGACTAATAGACTA |
| sequenom | SEQ011 | sf004112_01AC | sf004112_01AC_AG_F | ACGTTGGATGATATGGCCTGTTGAAGTGGG | sf004112_01AC_AG_R | ACGTTGGATGCAGACAGGATTAGGAACGAC | sf004112_01AC_AG_P | ACTCATCTAAACGCTCC |
| sequenom | SEQ011 | sf004602_04AG | sf004602_04AG_GA_F | ACGTTGGATGAAAGAGACAGTTCTGCCTCC | sf004602_04AG_GA_R | ACGTTGGATGCTAGGAATGCTTACTGTGAC | sf004602_04AG_GA02_Pa | TGGCCTCCATTACCTCAAAG |
| sequenom | SEQ011 | sf006081_01AT | sf006081_01AT_GT_F | ACGTTGGATGCGTGGCGGATAGAATTACTC | sf006081_01AT_GT_R | ACGTTGGATGGTCAGGGCATAGCACTTTTC | sf006081_01AT_GT_P | TTCCCGCCTTACATCAAAGTTACCTGAA |
| sequenom | SEQ012 | sf000023_02AC | sf000023_02AC_TC_F | ACGTTGGATGCAAACGTGATATCTGTCCCC | sf000023_02AC_TC_R | ACGTTGGATGAGTCCCCAGTCAAAGTGAAG | sf000023_02AC_TC_Pa | TTCCAATAATGGTCAGAATCA |
| sequenom | SEQ012 | sf000179_01AC | sf000179_01AC_TC_F | ACGTTGGATGACAGCGGAGGAGATTTGTTC | sf000179_01AC_TC_R | ACGTTGGATGACCTGCATTGACACAGATGG | sf000179_01AC_TC_Pa | GGGAGCTATAGTGACAGCAGTTAG |
| sequenom | SEQ012 | sf000209_01AG | sf000209_01AG_CT_F | ACGTTGGATGTCTGTTCATTAGCCAGAGCC | sf000209_01AG_CT_R | ACGTTGGATGCGGTCCGATCAGTTTTTCTC | sf000209_01AG_CT_P | AGCCATTGTGACTTAAATGTCTC |
| sequenom | SEQ012 | sf000230_01CG | sf000230_01CG_TA_F | ACGTTGGATGTGAAAGTAGCCTGGGTTGTG | sf000230_01CG_TA_R | ACGTTGGATGCATGAGCGTCACCAACCAAT | sf000230_01CG_TA02_Pa | TGCGTCACCAACCAATGAAAAG |
| sequenom | SEQ012 | sf000633_01AT | sf000633_01AT_GT_F | ACGTTGGATGGAGATTTCCACTCCTTCACC | sf000633_01AT_GT_R | ACGTTGGATGGCCGTTGATAGAACGGTAAC | sf000633_01AT_GT02_Pa | CAGGTCGTGGTTTTTAAATTGTCTGG |
| sequenom | SEQ012 | sf000706_01CT | sf000706_01CT_AG_F | ACGTTGGATGGGTCAATGAATGCATCCTCG | sf000706_01CT_AG_R | ACGTTGGATGTTGCTATGGGCTGGACAATG | sf000706_01CT_AG_P | GCTGGACAATGGCAGAGGTGTCCAATTGCA |
| sequenom | SEQ012 | sf000719_01CT | sf000719_01CT_AT_F | ACGTTGGATGTCAGCCTTCAGCTAAAACCC | sf000719_01CT_AT_R | ACGTTGGATGAATGGGAATGTCACTCGAGG | sf000719_01CT_AT_P | ATGAATGTGAATGTCACTCGAGGGGAGAG |
| sequenom | SEQ012 | sf000905_02CT | sf000905_02CT_AT_F | ACGTTGGATGCTTGTTATGGGCGTCTCTTC | sf000905_02CT_AT_R | ACGTTGGATGCTGCAATTGGTCAAGTTGGG | sf000905_02CT_AT_P | AAACAATACACTTTCGAGTTTGTACA |
| sequenom | SEQ012 | sf000966_03CT | sf000966_03CT_AG_F | ACGTTGGATGTCTCCCTGATGTGAGGAAAG | sf000966_03CT_AG_R | ACGTTGGATGCTGCAGCAGAACACACATAC | sf000966_03CT_AG_P | CCCCTCTGGTTGGCTCAGTCTT |
| sequenom | SEQ012 | sf001069_01AG | sf001069_01AG_GT_F | ACGTTGGATGCCTGGTTTTCTCCTCAGATG | sf001069_01AG_GT_R | ACGTTGGATGGGGACAAACCTGCATGTTTC | sf001069_01AG_GT02_P | GTTCTTCTCATGGGCTTT |
| sequenom | SEQ012 | sf001437_01AT | sf001437_01AT_AG_F | ACGTTGGATGTGTCGACCAGGGTGAGGTTT | sf001437_01AT_AG_R | ACGTTGGATGTTTCTCTTGTTCCAGGCGAG | sf001437_01AT_AG_P | GGGAAGGGTGAGGTTTGTCACAACC |
| sequenom | SEQ012 | sf001601_01AC | sf001601_01AC_CT_F | ACGTTGGATGTCCAAGACCATGGATGAGAG | sf001601_01AC_CT_R | ACGTTGGATGATTGCAGGGATGTGCAGTAG | sf001601_01AC_CT_P | ATGTCTGACCAATACTGTCCTAT |
| sequenom | SEQ012 | sf001644_01AG | sf001644_01AG_GT_F | ACGTTGGATGCAAACAGACATGTCATCACG | sf001644_01AG_GT_R | ACGTTGGATGTTACATGCACCAGATGCTCC | sf001644_01AG_GT_Pa | TTTTGGGGACCGAGCAAAGAATTTC |
| sequenom | SEQ012 | sf001686_01CT | sf001686_01CT_GA_F | ACGTTGGATGGAGTTGAGCCGTTCTGTATC | sf001686_01CT_GA_R | ACGTTGGATGTCTGTCAGACAGTCCAGGTG | sf001686_01CT_GA_Pa | GGCAACGTGGTGACAG |
| sequenom | SEQ012 | sf001996_02CG | sf001996_02CG_AG_F | ACGTTGGATGACCATGGGCCAAAAAGCTTG | sf001996_02CG_AG_R | ACGTTGGATGTTCATTCTCCACAGTGCCTC | sf001996_02CG_AG_P | TGCCTCACAAGTGCTA |
| sequenom | SEQ012 | sf002203_01CT | sf002203_01CT_AG_F | ACGTTGGATGGGTTCAATCCCTTTGCATCC | sf002203_01CT_AG_R | ACGTTGGATGGGTGGCAGAACACCAGTTTT | sf002203_01CT_AG_P | CCTTTCAATGCTTTTCAGC |
| sequenom | SEQ012 | sf002250_01AG | sf002250_01AG_CA_F | ACGTTGGATGAAAGATGGCTGATCTCCCTC | sf002250_01AG_CA_R | ACGTTGGATGTTGTGCACTTTTCGTCCTTG | sf002250_01AG_CA_P | CCCCACTACAAATGGGCCAAAACAAT |
| sequenom | SEQ012 | sf002353_02AT | sf002353_02AT_CT_F | ACGTTGGATGATTTCAACCAGCCATGGGAG | sf002353_02AT_CT_R | ACGTTGGATGAGTTGTTGCCACTCACCCTG | sf002353_02AT_CT_P | GGTGGTTGATGCCCTT |
| sequenom | SEQ012 | sf002792_01AG | sf002792_01AG_AG_F | ACGTTGGATGGTTCATTGTGGTGAACGGAC | sf002792_01AG_AG_R | ACGTTGGATGAAGAGTTTCAGTAGCCCTGC | sf002792_01AG_AG02_P | GGCAAGCCTCTGTAGAACAATGG |
| sequenom | SEQ012 | sf003076_01CT | sf003076_01CT_AG_F | ACGTTGGATGCAGTGGTTTAGATGGTACGC | sf003076_01CT_AG_R | ACGTTGGATGACACCGCTATTTCCTGGTTG | sf003076_01CT_AG_P | CCCTTTTGGTCACATAAACTGAAATTAGGC |
| sequenom | SEQ012 | sf003092_01CT | sf003092_01CT_TC_F | ACGTTGGATGTTCCAAGCCCTAGATTTGCC | sf003092_01CT_TC_R | ACGTTGGATGATATCAACTGACTGGAGGGC | sf003092_01CT_TC_Pa | ATTCATAATTCTATATCATTACAGC |
| sequenom | SEQ012 | sf003118_01GT | sf003118_01GT_GA_F | ACGTTGGATGCTATGAGGAGTGGTGTGTTC | sf003118_01GT_GA_R | ACGTTGGATGCCATACAAAACCCTCTGTAG | sf003118_01GT_GA_Pa | GGAAAAAACCCTCTGTAGTGTATGGCGG |
| sequenom | SEQ012 | sf003334_01CT | sf003334_01CT_AG_F | ACGTTGGATGTTATAGCTTCAAATAGGCCC | sf003334_01CT_AG_R | ACGTTGGATGGGCCTATGTAACCTAGCCAG | sf003334_01CT_AG_P | CCATTTATTTGTGTTCTGGAAAATGTGAAC |
| sequenom | SEQ012 | sf003500_02CT | sf003500_02CT_CT_F | ACGTTGGATGAGAGGCGCGATGAATTTAAG | sf003500_02CT_CT_R | ACGTTGGATGTGACAGCTGACAATGTGCTC | sf003500_02CT_CT02_P | CGTGCTCTTGGCTCGAGC |

| | | | | | | | | |
|---|---|---|---|---|---|---|---|---|
| sequenom | SEQ012 | sf003595_01CT | sf003595_01CT_AT_F | ACGTTGGATGTGTACACTTTTCCAATGC | sf003595_01CT_AT_R | ACGTTGGATGGCCTAGTCACAGCATTTACG | sf003595_01CT_AT_P | CACAATAAAGTGTGTACAG |
| sequenom | SEQ012 | sf003614_01CT | sf003614_01CT_GT_F | ACGTTGGATGGACCTGAAGTTCAGCAGTAG | sf003614_01CT_GT_R | ACGTTGGATGTCTGACTGACGGAACTGATG | sf003614_01CT_GT_Pa | CATCTTTAAACCTTACAGGT |
| sequenom | SEQ012 | sf003813_11CT | sf003813_11CT_AG_F | ACGTTGGATGAAGACATGGTTACTGACGGC | sf003813_11CT_AG_R | ACGTTGGATGTTCTCTCTCTCCCAGCCAAC | sf003813_11CT_AG02_P | CCTGACCCCAACGAGGAGCT |
| sequenom | SEQ012 | sf004055_02CT | sf004055_02CT_AT_F | ACGTTGGATGTAGCGCAGGATTTTACGACC | sf004055_02CT_AT_R | ACGTTGGATGCCAGTGTCAGTGTCCTAATG | sf004055_02CT_AT_P | CAGGGAGACTTGTAATTCAGC |
| sequenom | SEQ012 | sf006073_01CT | sf006073_01CT_GA_F | ACGTTGGATGAGCCTGGAGTGTTTTTTCTG | sf006073_01CT_GA_R | ACGTTGGATGAGTGGCCGTCGTCAAGTTTG | sf006073_01CT_GA_Pa | TCTGTTTGCTGCTAGTG |
| sequenom | SEQ013 | sf000002_01AG | sf000002_01AG_AG_F | ACGTTGGATGGCACAAGAGAGCTATATTAC | sf000002_01AG_AG_R | ACGTTGGATGGAGAGCCCTTCACAGTTTAG | sf000002_01AG_AG_P | CCTCCCTTTGCTACGTGGATA |
| sequenom | SEQ013 | sf000017_01CT | sf000017_01CT_GC_F | ACGTTGGATGCACAATGCAGATCCCAGATG | sf000017_01CT_GC_R | ACGTTGGATGGGCCTCCAGCTTTAATTGTG | sf000017_01CT_GC02_Pa | ATAAGTACTCTCTGAATGGGAT |
| sequenom | SEQ013 | sf000021_01AG | sf000021_01AG_CA_F | ACGTTGGATGTAATGACCTCTAGCCACCTC | sf000021_01AG_CA_R | ACGTTGGATGATAAACAGCTGTGTGGGACG | sf000021_01AG_CA_Pa | GGGATGGGTCTGCTGGAT |
| sequenom | SEQ013 | sf000086_01CT | sf000086_01CT_CT_F | ACGTTGGATGTATCCATAGGGCAAGCGTAG | sf000086_01CT_CT_R | ACGTTGGATGGCGAAGTCGTGACACAAAAG | sf000086_01CT_CT_P | AAACGTGCTTATAGACAAC |
| sequenom | SEQ013 | sf000088_01AC | sf000088_01AC_GA_F | ACGTTGGATGGGTTTGCGAGAACACAAAGC | sf000088_01AC_GA_R | ACGTTGGATGTCCACTGGTCTGGTGTATTC | sf000088_01AC_GA_Pa | CAACTATATTGTATTTAATAAACTTGGGAC |
| sequenom | SEQ013 | sf000119_01AG | sf000119_01AG_AG_F | ACGTTGGATGCAGCAGCTATTGTTGCTATC | sf000119_01AG_AG_R | ACGTTGGATGACAAACAGTGTCCTAGGGAG | sf000119_01AG_AG_P | GAATTACTTACAGCAGACTGAATACAGT |
| sequenom | SEQ013 | sf000139_01CG | sf000139_01CG_GT_F | ACGTTGGATGCCTAATGTTACGAAATACCC | sf000139_01CG_GT_R | ACGTTGGATGGCGGTCCTTTTGACTGCAAC | sf000139_01CG_GT_Pa | CAAGTATAAGCCTAATAGACACATTT |
| sequenom | SEQ013 | sf000157_01AT | sf000157_01AT_CA_F | ACGTTGGATGAGATCAGCAGAGCAGACAAG | sf000157_01AT_CA_R | ACGTTGGATGCGATATTTTCCCATGGCCTG | sf000157_01AT_CA_P | TGGAGTTGTGTAGTCTAA |
| sequenom | SEQ013 | sf000175_01CT | sf000175_01CT_GT_F | ACGTTGGATGTTGTCCCAACAGAAACACAG | sf000175_01CT_GT_R | ACGTTGGATGAGACGATTGGGCCAATTTCC | sf000175_01CT_GT02_P | CGGCCAATTTCCCAAAGCA |
| sequenom | SEQ013 | sf000392_01AC | sf000392_01AC_CT_F | ACGTTGGATGAGCCGTTTTGAAGCTGGTCC | sf000392_01AC_CT_R | ACGTTGGATGGTGATGCACCTCATTTGAGC | sf000392_01AC_CT_P | TTGGAATGCAGTTGGT |
| sequenom | SEQ013 | sf000583_02CT | sf000583_02CT_TC_F | ACGTTGGATGTTAGTGCTACCACTAGAGGG | sf000583_02CT_TC_R | ACGTTGGATGTTAGACTGGGCTTCTCTTCC | sf000583_02CT_TC_Pa | TCCACAGTTAGCTGCAGGCGTGC |
| sequenom | SEQ013 | sf000717_02AG | sf000717_02AG_TC_F | ACGTTGGATGGTTTCTGTTTCTCATCTCAC | sf000717_02AG_TC_R | ACGTTGGATGGTCTTGTAGGAAGTCAGATG | sf000717_02AG_TC02_Pa | CTGTGGCTTTGAAATGTATCC |
| sequenom | SEQ013 | sf000747_02CT | sf000747_02CT_CT_F | ACGTTGGATGATGTTGTATTCTGACACTGG | sf000747_02CT_CT_R | ACGTTGGATGAAAGTCACTCTTAAGGCCCC | sf000747_02CT_CT_P | AGTAAGGCCCCATGTAGGTT |
| sequenom | SEQ013 | sf000978_02CT | sf000978_02CT_GA_F | ACGTTGGATGGCGAACCGTCTTGCATTAAC | sf000978_02CT_GA_R | ACGTTGGATGGAAACGTCTCTAGTTGCCAG | sf000978_02CT_GA_Pa | TTGCTAGAACATTGCCA |
| sequenom | SEQ013 | sf001164_02GT | sf001164_02GT_TC_F | ACGTTGGATGAACTAGCTGGAAATGATCAC | sf001164_02GT_TC_R | ACGTTGGATGAAACCTCTGACGTCAGTAGC | sf001164_02GT_TC_Pa | GGAGATCACTGGACAAGTTTAAGG |
| sequenom | SEQ013 | sf001692_03CG | sf001692_03CG_CT_F | ACGTTGGATGTTCCTGTGGCCTTCTGTTTC | sf001692_03CG_CT_R | ACGTTGGATGGTAGGAAGAACAAAGGACGC | sf001692_03CG_CT_P | CAAAGTACGCTAACAAAGAGAAACGCTGAT |
| sequenom | SEQ013 | sf002333_01AC | sf002333_01AC_AG_F | ACGTTGGATGTGCCTTATCTGTATACGCTC | sf002333_01AC_AG_R | ACGTTGGATGTGCATCACACTACTGTACAC | sf002333_01AC_AG_P | GGGGAGGAACTGGAAATTGAAGTGC |
| sequenom | SEQ013 | sf003018_09CT | sf003018_09CT_AG_F | ACGTTGGATGGTTCAGCACTGCCTCATTTC | sf003018_09CT_AG_R | ACGTTGGATGGGTATGACGTTCACTCTGAC | sf003018_09CT_AG_P | CCCTATCCACCAGGTGGCT |
| sequenom | SEQ013 | sf003105_01CT | sf003105_01CT_TC_F | ACGTTGGATGCTAACTTTGCATCCGCACTG | sf003105_01CT_TC_R | ACGTTGGATGACAACTCCATGCACAAGGAC | sf003105_01CT_TC_Pa | GTCACGGAACATTTTACCAG |
| sequenom | SEQ013 | sf003208_01CT | sf003208_01CT_TC_F | ACGTTGGATGTACACAAACCTTAGCTCCCC | sf003208_01CT_TC_R | ACGTTGGATGTGTACAAAGAGAGGATGCGG | sf003208_01CT_TC_Pa | CTCACAGGCGGCCATCTTAATTT |
| sequenom | SEQ013 | sf003442_01CG | sf003442_01CG_AG_F | ACGTTGGATGAGCACAGCCAACCACTTTAC | sf003442_01CG_AG_R | ACGTTGGATGAAGCTGTCTCCTGGGCTTAA | sf003442_01CG_AG_P | GGTTACATTATGCAGGGCATC |
| sequenom | SEQ013 | sf003538_01AG | sf003538_01AG_GT_F | ACGTTGGATGCAAATCAAGCACTTCAAATG | sf003538_01AG_GT_R | ACGTTGGATGTGGTGCTACTTCACATGGAG | sf003538_01AG_GT_Pa | CCTCCTAGAGGCTACATATGCAAGACAA |
| sequenom | SEQ013 | sf003697_01CT | sf003697_01CT_CA_F | ACGTTGGATGCAAAAATGTGTGTCGTTGGG | sf003697_01CT_CA_R | ACGTTGGATGATGGGCAAAGAGGGCTATTC | sf003697_01CT_CA_Pa | CTGCAAAGAGGGCTATTCTAAAAT |

| | | | | | | | | |
|---|---|---|---|---|---|---|---|---|
| sequenom | SEQ013 | sf003715_01AT | sf003715_01AT_CT_F | ACGTTGGATGTAGATGTTCAGGCCAAGACC | sf003715_01AT_CT_R | ACGTTGGATGCTTACAAGTCTGGAAATGTG | sf003715_01AT_CT02_P | TGTGATAATGCATGGAATGC |
| sequenom | SEQ013 | sf003726_01CT | sf003726_01CT_GT_F | ACGTTGGATGCCATCATTCAACACTCCTCC | sf003726_01CT_GT_R | ACGTTGGATGTGCTCATCTAGCAACTCAGG | sf003726_01CT_GT02_P | CCAAACTCAGGTGCTTTCATTA |
| sequenom | SEQ013 | sf004016_01AT | sf004016_01AT_AG_F | ACGTTGGATGCCAGTCTGTCATAACCCATC | sf004016_01AT_AG_R | ACGTTGGATGGAACACAGAGACAATGGAGG | sf004016_01AT_AG_P | GGGGAATTTTGAGGTGTGTAATTACAACA |
| sequenom | SEQ013 | sf004099_01CG | sf004099_01CG_TA_F | ACGTTGGATGGGAAACACATATCTGGTGAG | sf004099_01CG_TA_R | ACGTTGGATGGTGGAGAGTTCATCATTAGC | sf004099_01CG_TA_Pa | GGGCAGATCTGTTGCCAATTGG |
| sequenom | SEQ013 | sf004299_15GT | sf004299_15GT_CT_F | ACGTTGGATGGGGACTCCACACATTACAAA | sf004299_15GT_CT_R | ACGTTGGATGAACTAACAGTTAGCATGGTG | sf004299_15GT_CT_P | GAAAAGCTGAACAAAGTAATTTTCTA |
| sequenom | SEQ013 | sf005138_03AG | sf005138_03AG_CA_F | ACGTTGGATGGGAAGGCATCCTCAAAATTC | sf005138_03AG_CA_R | ACGTTGGATGCTCATCACTGTATCGTGTGC | sf005138_03AG_CA_Pa | GGGGTAGTGTGCATTTCTCCAGGGTCGC |
| sequenom | SEQ014 | sf004149_01AG | sf004149_01AG_GC_F | ACGTTGGATGGGACTATGTACAACAAGTGG | sf004149_01AG_GC_R | ACGTTGGATGATGATGGCATGACTATGAGG | sf004149_01AG_GC_Pa | CCCTGTATTTTCATCCATATCGGAT |
| sequenom | SEQ014 | sf004155_01AG | sf004155_01AG_GT_F | ACGTTGGATGGCTTTTGCTTTTACTACGGC | sf004155_01AG_GT_R | ACGTTGGATGGATGCTGGTAGCCTAGAATA | sf004155_01AG_GT_P | CAGCCTAGAATAAATGTTATGGAAG |
| sequenom | SEQ014 | sf004209_01GT | sf004209_01GT_AT_F | ACGTTGGATGCCTCACGCTAACAATTACCC | sf004209_01GT_AT_R | ACGTTGGATGATCCCCAACACTCCTTTGTC | sf004209_01GT_AT_P | CACGCCTTTGTCGTACTATAGGC |
| sequenom | SEQ014 | sf004240_01AG | sf004240_01AG_GA_F | ACGTTGGATGTAAACTCCCCCCTCATACTC | sf004240_01AG_GA_R | ACGTTGGATGTATGACACCTCCAATGTGGG | sf004240_01AG_GA_Pa | CAGACAAAAAACTGCTCGA |
| sequenom | SEQ014 | sf004252_02GT | sf004252_02GT_GT_F | ACGTTGGATGGTTCATGTCTCAGACACTGG | sf004252_02GT_GT_R | ACGTTGGATGTGAGGAGGTATTAAGTCCAG | sf004252_02GT_GT_P | TGAAGGCTGCTGTGGCTCTGCT |
| sequenom | SEQ014 | sf004257_02CG | sf004257_02CG_GA_F | ACGTTGGATGTTATGTTCCACAAGTGGCCC | sf004257_02CG_GA_R | ACGTTGGATGTTAACATGGCAAGACAGAAC | sf004257_02CG_GA_Pa | AGACAGAACAAATTCTTATTTACAA |
| sequenom | SEQ014 | sf004260_01AT | sf004260_01AT_TC_F | ACGTTGGATGCAGAACTGGCAGATGGAAAG | sf004260_01AT_TC_R | ACGTTGGATGATTTGTCTTCTGCCGTTCCC | sf004260_01AT_TC_Pa | TCTTTCCCAATGCCCACAA |
| sequenom | SEQ014 | sf004387_01AG | sf004387_01AG_TC_F | ACGTTGGATGTCATTAGTGCTGCATCTGCG | sf004387_01AG_TC_R | ACGTTGGATGCCTACGTGTTAGATACAGGC | sf004387_01AG_TC_Pa | CTGCAACATTCCCAGTACAC |
| sequenom | SEQ014 | sf004426_01AG | sf004426_01AG_CT_F | ACGTTGGATGGAAGCACTGCTTTGTTACGG | sf004426_01AG_CT_R | ACGTTGGATGAGCAAAGCCCTACATCTCTG | sf004426_01AG_CT_P | CTGTTCCTGAATACAGGCAGCCTACA |
| sequenom | SEQ014 | sf004438_02CT | sf004438_02CT_CT_F | ACGTTGGATGACTGGACCTGGAAAGATTGG | sf004438_02CT_CT_R | ACGTTGGATGGTTAGTAGCCTAATTCACAG | sf004438_02CT_CT_P | GGTAACAGTAAACAATAGAGCTAGA |
| sequenom | SEQ014 | sf004529_01CT | sf004529_01CT_AT_F | ACGTTGGATGGATCTCATTTTAGCAGCGAC | sf004529_01CT_AT_R | ACGTTGGATGGGCCAGATATTGCAATTGCG | sf004529_01CT_AT_P | CTCCATCAGACAAGTGAC |
| sequenom | SEQ014 | sf004545_01AC | sf004545_01AC_CA_F | ACGTTGGATGCCTTATATTAAACAAACCAG | sf004545_01AC_CA_R | ACGTTGGATGTGTCTTAGTGTTGGTGTGCC | sf004545_01AC_CA_Pa | TTTTATTGTAATTTATTTTGTTTACAATT |
| sequenom | SEQ014 | sf004562_01AG | sf004562_01AG_CA_F | ACGTTGGATGTCAAAGTGCACAGTGAGGAG | sf004562_01AG_CA_R | ACGTTGGATGTAACCTATTGGCTAGCCCAG | sf004562_01AG_CA_Pa | GTTAGGAATGGTCAACACAG |
| sequenom | SEQ014 | sf004632_01AG | sf004632_01AG_CT_F | ACGTTGGATGGAAAGACAAATGTTTCTTTCG | sf004632_01AG_CT_R | ACGTTGGATGAGAGTAGGTGTATAACGTGG | sf004632_01AG_CT_P | CAACGATAACTAAGAAGTAGAA |
| sequenom | SEQ014 | sf004642_07AG | sf004642_07AG_AG_F | ACGTTGGATGCGTCCATAATGTCAACAGGG | sf004642_07AG_AG_R | ACGTTGGATGACCTCTGGTTTGGAGTTGTG | sf004642_07AG_AG_P | GGGAGGGGTGATTGAACTGCAC |
| sequenom | SEQ014 | sf004684_01AG | sf004684_01AG_AT_F | ACGTTGGATGGTAGGTTGGTAAGACTGGAG | sf004684_01AG_AT_R | ACGTTGGATGAACTGACATGCCAAGGATCG | sf004684_01AG_AT_P | ACTCATATCGACCATATATTGTGTTCCC |
| sequenom | SEQ014 | sf004732_01CG | sf004732_01CG_CT_F | ACGTTGGATGTTCAACAGACTTTTTCCCGC | sf004732_01CG_CT_R | ACGTTGGATGAGATTGCCTCTGACTGTTCC | sf004732_01CG_CT_P | TTCCCCACGTTCTTTG |
| sequenom | SEQ014 | sf004762_01AT | sf004762_01AT_CT_F | ACGTTGGATGTCGCTTGCAGCAATGTTGAG | sf004762_01AT_CT_R | ACGTTGGATGATAACAGCAGCAGAGGAGTC | sf004762_01AT_CT_P | CCCCTCTCATCGGCAACTAATA |
| sequenom | SEQ014 | sf004765_01CG | sf004765_01CG_CT_F | ACGTTGGATGGCACCTGTAATGAGTGAATG | sf004765_01CG_CT_R | ACGTTGGATGGTTGTGTGACTCACATGCTG | sf004765_01CG_CT_P | TTACCACAAGGGATGACAA |
| sequenom | SEQ014 | sf004817_02GT | sf004817_02GT_CG_F | ACGTTGGATGTGGGTGTCATAAAGAAAGGG | sf004817_02GT_CG_R | ACGTTGGATGACTGTCCCTATTCCTGTTAG | sf004817_02GT_CG_P | GACATGACTTTACCATGTTG |
| sequenom | SEQ014 | sf004818_06CT | sf004818_06CT_CA_F | ACGTTGGATGGGGATAGCCAATCCAGATTC | sf004818_06CT_CA_R | ACGTTGGATGCATTAAGGCCACATAGCACC | sf004818_06CT_CA_Pa | CGGAGTTGGTCATTTTGG |
| sequenom | SEQ014 | sf004848_01CT | sf004848_01CT_AT_F | ACGTTGGATGCAAGACATGGCATAAGAGGG | sf004848_01CT_AT_R | ACGTTGGATGTAACGACGAACGATTGCTTG | sf004848_01CT_AT_P | TAAAGTATCGTCCAACCC |

| | | | | | | | | |
|---|---|---|---|---|---|---|---|---|
| sequenom | SEQ014 | sf004870_01CG | sf004870_01CG_GA_F | ACGTTGGATGCACGCCTTACAACAGAATAG | sf004870_01CG_GA_R | ACGTTGGATGGAAGTCTGCCCAGTCAAAAG | sf004870_01CG_GA_Pa | GAGGATTAATAATTATACTGTACAAG |
| sequenom | SEQ014 | sf004904_09CT | sf004904_09CT_CG_F | ACGTTGGATGGGCATGCAGAGTTCAATTAG | sf004904_09CT_CG_R | ACGTTGGATGAAGACCGGGCATTCAACTTG | sf004904_09CT_CG_P | TTGAAGTTGATACTGTTTCCATGGTAA |
| sequenom | SEQ014 | sf004909_01GT | sf004909_01GT_TA_F | ACGTTGGATGGCCCTGTTAACTCCTTAGTG | sf004909_01GT_TA_R | ACGTTGGATGATGGGACAATGATCACTGAG | sf004909_01GT_TA_Pa | GAGCACTGAGATCATATGCAAAT |
| sequenom | SEQ014 | sf004913_01GT | sf004913_01GT_CA_F | ACGTTGGATGGAAGCCAGATGTTAAGATGAC | sf004913_01GT_CA_R | ACGTTGGATGCCCCAGATTTGAGTCTGTAG | sf004913_01GT_CA_P | TTTCAAATGACAAGGCAATCTCACAA |
| sequenom | SEQ014 | sf004975_01CG | sf004975_01CG_TC_F | ACGTTGGATGGGAAGTTTGCTGCAAATGAG | sf004975_01CG_TC_R | ACGTTGGATGAACGGCCTCGTACTCTATTC | sf004975_01CG_TC_Pa | CAGCCTCGTACTCTATTCTAGATC |
| sequenom | SEQ014 | sf004977_01AT | sf004977_01AT_CT_F | ACGTTGGATGGCCTTGATGTTCATCAGTCC | sf004977_01AT_CT_R | ACGTTGGATGAGTCATCTTTCCCTTCCAGC | sf004977_01AT_CT_P | CCTCTCCATTTATAGACAATTTGTC |
| sequenom | SEQ014 | sf005000_09CT | sf005000_09CT_TC_F | ACGTTGGATGATGCCGTTCTGTACCATCAC | sf005000_09CT_TC_R | ACGTTGGATGCCTAACAATTCCACAACTAC | sf005000_09CT_TC_Pa | GTCCACAACTACTACCTTATACAC |
| sequenom | SEQ014 | sf005054_02AC | sf005054_02AC_GA_F | ACGTTGGATGCCACCCAACTACTCAATTCC | sf005054_02AC_GA_R | ACGTTGGATGTGTTCTCAATTGAGGCGCAG | sf005054_02AC_GA_Pa | GAGGCGCAGATAAGCG |
| sequenom | SEQ014 | sf005079_01AG | sf005079_01AG_CA_F | ACGTTGGATGACCCACCATCACAACTCGAC | sf005079_01AG_CA_R | ACGTTGGATGGAACATCTGAAATATTGTCTG | sf005079_01AG_CA_P | ATTCCGATCTGTGTCTTTCCATTGCTGTG |
| sequenom | SEQ014 | sf005248_01AG | sf005248_01AG_CT_F | ACGTTGGATGTTGCTGGGATTTACCACCAC | sf005248_01AG_CT_R | ACGTTGGATGGGGTAGCCAATCCAGATTTC | sf005248_01AG_CT_P | TTACACACTCAGGTATAGCAC |
| sequenom | SEQ014 | sf005272_01CG | sf005272_01CG_GC_F | ACGTTGGATGAGTATGAGGGAGTGTGAGAG | sf005272_01CG_GC_R | ACGTTGGATGTTAGCCATTTTTCCTCCCCG | sf005272_01CG_GC_Pa | CCGGTGTCATGTGACT |
| sequenom | SEQ014 | sf005409_04AT | sf005409_04AT_CA_F | ACGTTGGATGAGATGTATTGGAGTAGTGCC | sf005409_04AT_CA_R | ACGTTGGATGTCAGGCATTTACAGCCTCAC | sf005409_04AT_CA_P | TGTGGTATGGCATCAGACAGAAAT |
| sequenom | SEQ014 | sf005410_01AG | sf005410_01AG_CA_F | ACGTTGGATGGGATGAGCAATGTCAGAGCG | sf005410_01AG_CA_R | ACGTTGGATGTATCCATGCCCAATGGGAAC | sf005410_01AG_CA_Pa | ACTCCATTCCGGCTCCA |
| sequenom | SEQ014 | sf005420_01CG | sf005420_01CG_GA_F | ACGTTGGATGTTGAAGATGGTGATGGTCTC | sf005420_01CG_GA_R | ACGTTGGATGGATGCCATCAAGGCCAAAAC | sf005420_01CG_GA_Pa | ACACTTTAGTGTTTTAAAGCA |
| sequenom | SEQ015 | sf000020_CT | sf000020_CT_CT_F | ACGTTGGATGGGCGATCTCTGTTCCTAATG | sf000020_CT_CT_R | ACGTTGGATGGGTAAAGTGGTAGAGGTAGC | sf000020_CT_CT_P | AGATCAGATTATTCTTTGTGAGAG |
| sequenom | SEQ015 | sf000382_AG | sf000382_AG_GA_F | ACGTTGGATGTGGCAAAATCAAAGCCAGAC | sf000382_AG_GA_R | ACGTTGGATGGTTTGTAGACTGGCTACATC | sf000382_AG_GA_Pa | GGCTACATCTTATATCATCATCT |
| sequenom | SEQ015 | sf000508_CT | sf000508_CT_TC_F | ACGTTGGATGGTGTGTGACATATTCTCCCC | sf000508_CT_TC_R | ACGTTGGATGTTGCTCAGAGAGCCAGAGAG | sf000508_CT_TC_Pa | GAGTTACACAGAGACGTGTCATC |
| sequenom | SEQ015 | sf000559_AG | sf000559_AG_GA_F | ACGTTGGATGGGTGATAGTGGGACTGATAG | sf000559_AG_GA_R | ACGTTGGATGAAGAAGAGCAGGTTGCCCAG | sf000559_AG_GA_Pa | GGGTGTCCTTCTCCTTCATAGGAC |
| sequenom | SEQ015 | sf000681_AT | sf000681_AT_AT_F | ACGTTGGATGGATACTCCCACACAAAACCC | sf000681_AT_AT_R | ACGTTGGATGGAGGTATGAGGTATTGGAGG | sf000681_AT_AT_P | GCAAGGTAAAGTATGTAGTAT |
| sequenom | SEQ015 | sf000684_CT | sf000684_CT_TC_F | ACGTTGGATGTGTATAATGAGTAGGCCCCC | sf000684_CT_TC_R | ACGTTGGATGGCATGCTCCACTTTTGAACC | sf000684_CT_TC_Pa | CCCCTCTTTGTATAACCTATGCATACAC |
| sequenom | SEQ015 | sf000744_AT | sf000744_AT_AT_F | ACGTTGGATGCAGTGAAGGGAACTGACCAC | sf000744_AT_AT_R | ACGTTGGATGCACTCGGTATGTTCTTCCTC | sf000744_AT_AT_P | CTATGGTCTTCCTCTGTATCTTGAGAGCTG |
| sequenom | SEQ015 | sf001105_AG | sf001105_AG_AG_F | ACGTTGGATGTGTCTCTGAAAGCACCAATC | sf001105_AG_AG_R | ACGTTGGATGTACCACACATGGCACATGAG | sf001105_AG_AG_P | CCTCCTTATATTAATAACTCCCCTGTAGT |
| sequenom | SEQ015 | sf001667_AG | sf001667_AG_GA_F | ACGTTGGATGGCCTCTCACACAAACTGTAG | sf001667_AG_GA_R | ACGTTGGATGAGTCACTGCAATTCACAGCC | sf001667_AG_GA_Pa | TGGGCCGGGTTTAGATT |
| sequenom | SEQ015 | sf001831_AC | sf001831_AC_CA_F | ACGTTGGATGCCAGTACATTTCCAGCTGTG | sf001831_AC_CA_R | ACGTTGGATGTCATTGGTATCAACTGATGC | sf001831_AC_CA_Pa | CTCCTCAACTGATGCCCTACT |
| sequenom | SEQ015 | sf002145_CG | sf002145_CG_CG_F | ACGTTGGATGTCTGACAGGTACACACACAG | sf002145_CG_CG_R | ACGTTGGATGGTCATTCTGGACAGGAGTTC | sf002145_CG_CG_P | GCAGTCTGGACAGGAGTTCCTACAGTA |
| sequenom | SEQ015 | sf003455_AG | sf003455_AG_AG_F | ACGTTGGATGATAACCAGTAGGGCAACGTC | sf003455_AG_AG_R | ACGTTGGATGTATACGAAGTCGCTGACAAC | sf003455_AG_AG_P | GGTCGTTGATATAAATGTTAATGAATTAGC |
| sequenom | SEQ015 | sf003520_AG | sf003520_AG_GA_F | ACGTTGGATGGATGCAGGGAGACAGTATTG | sf003520_AG_GA_R | ACGTTGGATGATGAGAGAGGATAGAAAGGG | sf003520_AG_GA_Pa | GGCAGAATGAATATCATGTCAATTA |
| sequenom | SEQ015 | sf003994_GT | sf003994_GT_GT_F | ACGTTGGATGCCCCATGTATGTTTCTCAAG | sf003994_GT_GT_R | ACGTTGGATGTCAAGCCCCAACAATATAGG | sf003994_GT_GT_P | ACCTCTTTAAGAGCTACACATTTGT |

| | | | | | | | | |
|---|---|---|---|---|---|---|---|---|
| sequenom | SEQ015 | sf004013_AC | sf004013_AC_CA_F | ACGTTGGATGTGTGTTGTGCGTGCATG | sf004013_AC_CA_R | ACGTTGGATGGGATTTGGGAAGTGAACTGC | sf004013_AC_CA_P | CCTCACACTGACACCA |
| sequenom | SEQ015 | sf004038_AG | sf004038_AG_AG_F | ACGTTGGATGAACTAGAGCGGATAACCATC | sf004038_AG_AG_R | ACGTTGGATGCCGGGTGTAAGATAACAATG | sf004038_AG_AG_P | CCCCAGGGTTAAACCGTTCC |
| sequenom | SEQ015 | sf004090_CG | sf004090_CG_GC_F | ACGTTGGATGAATAAACCAGGCCAGAGATG | sf004090_CG_GC_R | ACGTTGGATGCGGCCGTTTTATGGTTTTGC | sf004090_CG_GC_Pa | CCGCCTGTCAACCTCTATT |
| sequenom | SEQ015 | sf004131_AG | sf004131_AG_AG_F | ACGTTGGATGGAAAACCCCAAACCAGGAAC | sf004131_AG_AG_R | ACGTTGGATGAGATTCTGCGTGTAAACGCC | sf004131_AG_AG_P | GGGGTACGTAGAATGATGTGATTAC |
| sequenom | SEQ015 | sf004357_CT | sf004357_CT_CT_F | ACGTTGGATGAACTGCAAACATGGCAACCC | sf004357_CT_CT_R | ACGTTGGATGATGTCTAATTCCTCCGCTCC | sf004357_CT_CT_P | TGTTGCAGCAATGATAATGTGGA |
| sequenom | SEQ015 | sf004379_CT | sf004379_CT_TC_F | ACGTTGGATGGTGAGGTCAACTCAGAGTTC | sf004379_CT_TC_R | ACGTTGGATGGTTTCCTAGGTGACCTGGAG | sf004379_CT_TC_Pa | GACCTGGAGGTGAGCC |
| sequenom | SEQ015 | sf004475_CT | sf004475_CT_TC_F | ACGTTGGATGCCGATGTTGTACCTTTGAGC | sf004475_CT_TC_R | ACGTTGGATGTTTGTGTCCCAAATGGCACC | sf004475_CT_TC_Pa | ATGGCACCCTATGTATAATACACTATATA |
| sequenom | SEQ015 | sf004521_CG | sf004521_CG_CG_F | ACGTTGGATGGATCCATGTGATTTCGTGCC | sf004521_CG_CG_R | ACGTTGGATGGTGTCAGGAGAAACGTTAC | sf004521_CG_CG_P | ACATATAGTGATATAAATGATTTTGTATA |
| sequenom | SEQ015 | sf004541_AC | sf004541_AC_CA_F | ACGTTGGATGCCCCTCTTTTCTCATTCCTC | sf004541_AC_CA_R | ACGTTGGATGCGCATGACACCACAATTGAG | sf004541_AC_CA_Pa | CCCATCTAACTAAATCCCAGACGACA |
| sequenom | SEQ015 | sf004614_AG | sf004614_AG_AG_F | ACGTTGGATGGGAACTAGCGCTATCAATGG | sf004614_AG_AG_R | ACGTTGGATGCAGGCCTGATTTCCCACATA | sf004614_AG_AG_P | CCCTTTCCCACATAATTCCCTTCACTC |
| sequenom | SEQ015 | sf004897_AT | sf004897_AT_TA_F | ACGTTGGATGGTGGCTATGACGGTGATTTC | sf004897_AT_TA_R | ACGTTGGATGTTTTTGCGTCTGTTTCCCCC | sf004897_AT_TA_Pa | GGCATAGCTGCCTATTAAAG |
| sequenom | SEQ015 | sf005186_CT | sf005186_CT_TC_F | ACGTTGGATGCCTTTCTTCCAACCTCCATC | sf005186_CT_TC_R | ACGTTGGATGGTTGACCAGGGCTTTGTTAG | sf005186_CT_TC_Pa | CTTGTTAGTAACACTCCTAGA |
| sequenom | SEQ015 | sf005258_AC | sf005258_AC_CA_F | ACGTTGGATGTTGGCTTCCTGGCTTGTAAC | sf005258_AC_CA_R | ACGTTGGATGAGAGCTCCCCCATCTGATAG | sf005258_AC_CA_Pa | CGATAGCAGAGTCGAAGTA |
| sequenom | SEQ015 | sf005286_CT | sf005286_CT_CT_F | ACGTTGGATGAAAAAGTTGACGCGCGTCTG | sf005286_CT_CT_R | ACGTTGGATGCCACTCTTTCCCGATTTACC | sf005286_CT_CT_P | ACTTGCACGATCTACAC |
| sequenom | SEQ015 | sf005443_CG | sf005443_CG_GC_F | ACGTTGGATGGCAATCACAACCAATTGAGC | sf005443_CG_GC_R | ACGTTGGATGTCCATCTTGGCCATCCATTC | sf005443_CG_GC_Pa | TCAAGCGTTGAAGTCAATATCT |
| sequenom | SEQ015 | sf005501_AG | sf005501_AG_GA_F | ACGTTGGATGAGACACATCCTGCCATTTGC | sf005501_AG_GA_R | ACGTTGGATGTTTTCCAAGTACCAAAGCTG | sf005501_AG_GA_Pa | ATGTTCCTTACTCTGTGG |
| sequenom | SEQ015 | sf005540_CT | sf005540_CT_TC_F | ACGTTGGATGCAGAGCATTTATCCCACAAC | sf005540_CT_TC_R | ACGTTGGATGTGGACATCTCAATTTCTCCC | sf005540_CT_TC_Pa | ACTTATGCAAACACTTGCAACATGACCT |
| sequenom | SEQ015 | sf005590_CT | sf005590_CT_TC_F | ACGTTGGATGTGCAGTGTGTGACTGAAAGC | sf005590_CT_TC_R | ACGTTGGATGATGTGACATGTGACGGTGAG | sf005590_CT_TC_Pa | AGGGGAGACAGTGATAGTGGTAT |
| sequenom | SEQ015 | sf005652_AG | sf005652_AG_GA_F | ACGTTGGATGGAGCGAGCATGAATATGA | sf005652_AG_GA_R | ACGTTGGATGTGCATTTTAGGCTGTCGCTG | sf005652_AG_GA_Pa | GACACCATTTCACTGTTGT |
| sequenom | SEQ015 | sf005654_CT | sf005654_CT_CT_F | ACGTTGGATGATCTACCGGCACAATCTCAC | sf005654_CT_CT_R | ACGTTGGATGTAAATGACGTTTCTGGCGGG | sf005654_CT_CT_P | CTCACTATAGCCTTTGCATCACACTGTTGT |
| sequenom | SEQ015 | sf005769_CTb | sf005769_CTb_CT_F | ACGTTGGATGGCCGCTGAAAAGAATCCTTG | sf005769_CTb_CT_R | ACGTTGGATGTGTGTATCTGGACCACACTG | sf005769_CTb_CT_P | TTTCCTCCTGAGAGCA |
| sequenom | SEQ015 | sf006000_AG | sf006000_AG_GA_F | ACGTTGGATGATTGACACACCATAAGTAGC | sf006000_AG_GA_R | ACGTTGGATGACGTTGGCAGATGCAGTGGA | sf006000_AG_GA_Pa | GATGCAGTGGATTGAGA |
| sequenom | SEQ015 | sf006410_AG | sf006410_AG_GA_F | ACGTTGGATGCTGTTGAAAAGGCAGTCATC | sf006410_AG_GA_R | ACGTTGGATGACTCAAGACCCTCACATAAG | sf006410_AG_GA_Pa | AACCATGTTCTATGATTTCTAATATTT |
| sequenom | SEQ016 | sf000518_GT | sf000518_GT_GT_F | ACGTTGGATGCCACTTCACTGTTATGCTGG | sf000518_GT_GT_R | ACGTTGGATGGGCTAGTGGAAGAAGACATC | sf000518_GT_GT_P | GCTGAGAAACCATTGAGCTCAGAAATAT |
| sequenom | SEQ016 | sf000554_CT | sf000554_CT_TC_F | ACGTTGGATGCAGGTTGACTGTGTCTCTAC | sf000554_CT_TC_R | ACGTTGGATGCTAGCCAGGTCTCTAGACAG | sf000554_CT_TC_Pa | GAGGGGAGAGACAGCATGCATGTA |
| sequenom | SEQ016 | sf000656_AG | sf000656_AG_GA_F | ACGTTGGATGCCGACTGCATATGAATAGAG | sf000656_AG_GA_R | ACGTTGGATGTACGCGTGATTGGATTCCTG | sf000656_AG_GA_Pa | ATCCCAATAAATTATTTCTCCCAATCA |
| sequenom | SEQ016 | sf001196_AG | sf001196_AG_GA_F | ACGTTGGATGAATGGGATACGCTGTCTGAG | sf001196_AG_GA_R | ACGTTGGATGTACAGTCACCTTTGCCATCC | sf001196_AG_GA_Pa | GCTTGCGTTAATGACCT |
| sequenom | SEQ016 | sf001288_CT | sf001288_CT_CT_F | ACGTTGGATGAGACCAGACACAAACATGAG | sf001288_CT_CT_R | ACGTTGGATGTCCAGTCACCAGATGGATTG | sf001288_CT_CT_P | AGCTTATTCATCTGCCACA |

| | | | | | | | | |
|---|---|---|---|---|---|---|---|---|
| sequenom | SEQ016 | sf001359_AG | sf001359_AG_GA_F | ACGTTGGATGCCGGAGTGCTTATAGTACAG | sf001359_AG_GA_R | ACGTTGGATGCATCCCTTTCCTTTGAATAC | sf001359_AG_GA_Pa | GGCGTTGAATACACATTGTCATTTATATCA |
| sequenom | SEQ016 | sf001812_AG | sf001812_AG_AG_F | ACGTTGGATGTGTGCAACTGCAGCTTTACG | sf001812_AG_AG_R | ACGTTGGATGCCCATCTGCTGGAACCAAC | sf001812_AG_AG_P | TGGAACCAACCCTTTC |
| sequenom | SEQ016 | sf001916_AG | sf001916_AG_GA_F | ACGTTGGATGAAGAACAGTGTACCGGAAAG | sf001916_AG_GA_R | ACGTTGGATGCGGCAAGTTAGCTAATGTTC | sf001916_AG_GA_Pa | GTTCCGAATATGAGGCAATC |
| sequenom | SEQ016 | sf002018_AC | sf002018_AC_CA_F | ACGTTGGATGGATTGAGGTGGAGCTGATTG | sf002018_AC_CA_R | ACGTTGGATGCCCAGGAAGGTTTTATAACTG | sf002018_AC_CA_P | ACAGGAAGGTTTTATAACTGTAATAA |
| sequenom | SEQ016 | sf002131_AG | sf002131_AG_GA_F | ACGTTGGATGGTCATCAGTATCACATTGGC | sf002131_AG_GA_R | ACGTTGGATGCTGACACTGCTATCTTTGCC | sf002131_AG_GA_Pa | GGGGAGTCTATTAAACATGGAGTCATGTA |
| sequenom | SEQ016 | sf002439_CT | sf002439_CT_CT_F | ACGTTGGATGTTCCACGGCCTGTAAGTAAC | sf002439_CT_CT_R | ACGTTGGATGGATAACACGCTGAGTCAACC | sf002439_CT_CT_P | CGCTGAGTCAACCTAAAGAT |
| sequenom | SEQ016 | sf002640_AG | sf002640_AG_AG_F | ACGTTGGATGCCCTTACACAATCAGTTTGC | sf002640_AG_AG_R | ACGTTGGATGCTTGAGATGATTCCCACAGG | sf002640_AG_AG_P | TGTCAACTCTCCCTCC |
| sequenom | SEQ016 | sf003633_AG | sf003633_AG_GA_F | ACGTTGGATGGAAGGTATTGTGAGAGACGC | sf003633_AG_GA_R | ACGTTGGATGTACACACCTCCACATGGAAC | sf003633_AG_GA_Pa | CACATGGAACATTAAAGAACAAG |
| sequenom | SEQ016 | sf003647_CG | sf003647_CG_GC_F | ACGTTGGATGGCAATCACAACCAATTGAGC | sf003647_CG_GC_R | ACGTTGGATGTCCATCTTGGCCATCCATTC | sf003647_CG_GC_Pa | CTCTGTCAAGCGTTGAAGTCAATATCT |
| sequenom | SEQ016 | sf003788_GT | sf003788_GT_GT_F | ACGTTGGATGTTATTGCAGTCCATTTGTG | sf003788_GT_GT_R | ACGTTGGATGCTCCATCAAGAAGTCATGGG | sf003788_GT_GT_Pa | GAATCTTGAGCTATCAGTTATCCTGTAT |
| sequenom | SEQ016 | sf004056_CT | sf004056_CT_TC_F | ACGTTGGATGGAAGGATGTCATTGGACCAG | sf004056_CT_TC_R | ACGTTGGATGAACCTCTGGATTGTCAAGGG | sf004056_CT_TC_Pa | CTTAATCAAATCTCCCCTTGAATCTATC |
| sequenom | SEQ016 | sf004192_AG | sf004192_AG_GA_F | ACGTTGGATGGTGTGAGAATAGCCTAGATG | sf004192_AG_GA_R | ACGTTGGATGTGGGAGTCTGCTGAATAGTC | sf004192_AG_GA_Pa | AGAAAATCTTAGGATTTTGATACCATTATA |
| sequenom | SEQ016 | sf004214_AG | sf004214_AG_GA_F | ACGTTGGATGAACTACCTGTACCCTTTGTC | sf004214_AG_GA_R | ACGTTGGATGGGGTGTGATTTATCAGTATG | sf004214_AG_GA_Pa | CCCTTTACTCTTTCAAATTCTACTGTATGA |
| sequenom | SEQ016 | sf004288_AG | sf004288_AG_GA_F | ACGTTGGATGGAAGCATAGTGAATTAAAC | sf004288_AG_GA_R | ACGTTGGATGCCTAACCCACTCACAATATG | sf004288_AG_GA_Pa | TTCACCCAAAAGGCATT |
| sequenom | SEQ016 | sf004416_AC | sf004416_AC_CA_F | ACGTTGGATGTCCCGTTGTTTGATGCTTGG | sf004416_AC_CA_R | ACGTTGGATGTCCTCCATATGTATCAGCAG | sf004416_AC_CA_P | GGGGAGCAGAAATAAGACCGAT |
| sequenom | SEQ016 | sf004423_AG | sf004423_AG_AG_F | ACGTTGGATGACACAGCAGTGAATCTTTCC | sf004423_AG_AG_R | ACGTTGGATGGGCCACCTAATCAACATTTC | sf004423_AG_AG_P | TTCCACAAGGTCTTTCCCTG |
| sequenom | SEQ016 | sf004714_CT | sf004714_CT_CT_F | ACGTTGGATGCAGATGCAGACTCAGATTTT | sf004714_CT_CT_R | ACGTTGGATGACACATTGGCTGTGTACAGG | sf004714_CT_CT_P | ATACTGGTGCATAGAGTTGTA |
| sequenom | SEQ016 | sf004795_CT | sf004795_CT_CT_F | ACGTTGGATGGGGATCTTTCAAGAAGCTTAG | sf004795_CT_CT_R | ACGTTGGATGCCAGCAGTTATGTCATGCAG | sf004795_CT_CT_P | AGAAATAAGGCATAACTGTTAAGAA |
| sequenom | SEQ016 | sf004811_AT | sf004811_AT_TA_F | ACGTTGGATGCATGAAGAAAATCAAGGTCTC | sf004811_AT_TA_R | ACGTTGGATGCTCTTCAAAACAACTGTGTC | sf004811_AT_TA_Pa | AATACCCGTTATAGCATAATCGTTA |
| sequenom | SEQ016 | sf004890_AC | sf004890_AC_CA_F | ACGTTGGATGGAACAGAGCAATACTCAGTC | sf004890_AC_CA_R | ACGTTGGATGAGGTTTCTACCCTACATTCC | sf004890_AC_CA_P | TCTATAGAATTTCTTAATATTATGTAGTT |
| sequenom | SEQ016 | sf004917_CT | sf004917_CT_TC_F | ACGTTGGATGAGCAATAAGATATGTTGGGG | sf004917_CT_TC_R | ACGTTGGATGAATCTGTTTCCTGCCGTCTG | sf004917_CT_TC_Pa | AGGGGTAGGGGACTTGCCTGAAC |
| sequenom | SEQ016 | sf004984_AG | sf004984_AG_GA_F | ACGTTGGATGGGGATGTGCAGACATTATTC | sf004984_AG_GA_R | ACGTTGGATGTGCACCCATAGTTATGTCAC | sf004984_AG_GA_Pa | CATAGTTATGTCACATGCTTTC |
| sequenom | SEQ016 | sf005052_AG | sf005052_AG_GA_F | ACGTTGGATGTAGAACCGATCTGGGACTC | sf005052_AG_GA_R | ACGTTGGATGATTACACATACCCGAGACCC | sf005052_AG_GA_Pa | TACCCGTGACAATCAGATCAGA |
| sequenom | SEQ016 | sf005168_AC | sf005168_AC_CA_F | ACGTTGGATGGGCCCAGTGGAAATGAATAG | sf005168_AC_CA_R | ACGTTGGATGTGAGTGTTACTTCTCCCGTG | sf005168_AC_CA_Pa | AATGTGGTGGATTATTGAAG |
| sequenom | SEQ016 | sf005344_AT | sf005344_AT_TA_F | ACGTTGGATGCACAAGCGTCACATGTCTTC | sf005344_AT_TA_R | ACGTTGGATGATTAACCCCTTTTTGGGTAG | sf005344_AT_TA_Pa | TTTGGGTAGGCACAAA |
| sequenom | SEQ016 | sf005440_CT | sf005440_CT_TC_F | ACGTTGGATGGATACAAGGAAAAAGATTGG | sf005440_CT_TC_R | ACGTTGGATGTCCACAACATGAACAGACAC | sf005440_CT_TC_Pa | CCAAATGAACAGACACAATATCAC |
| sequenom | SEQ016 | sf005459_AC | sf005459_AC_CA_F | ACGTTGGATGAAGCATAATGGAGTCCCCTG | sf005459_AC_CA_R | ACGTTGGATGGTCTGATTTACCTCATGCAG | sf005459_AC_CA_Pa | TAACAGACACATGTTTAAAAAATG |
| sequenom | SEQ016 | sf005663_CT | sf005663_CT_TC_F | ACGTTGGATGACAACCTGTTGATCGAGGAC | sf005663_CT_TC_R | ACGTTGGATGTCACAGAGCTGACTGATCAC | sf005663_CT_TC_Pa | TGGGTGTATGTGTGCGCGA |

| | | | | | | | | |
|---|---|---|---|---|---|---|---|---|
| sequenom | SEQ016 | sf005769_Cta | sf005769_CTa_CT_F | ACGTTGGATGGAGAGCTATCATCCAGATCG | sf005769_CTa_CT_R | ACGTTGGATGGCAAAACGTCTTCTAGTTGG | sf005769_CTa_CT_P | AAAGTTGGGCTTCCATAG |
| sequenom | SEQ016 | sf006000_CT | sf006000_CT_CT_F | ACGTTGGATGTTGGCAGATGCAGTGGATTG | sf006000_CT_CT_R | ACGTTGGATGATTGACACACCATAAGTAGC | sf006000_CT_CT_P | AGAGATTTTTGCATGGGC |
| sequenom | SEQ017 | sf000132_AG | sf000132_AG_GA_F | ACGTTGGATGAGTAACACAGATCCGCCTTG | sf000132_AG_GA_R | ACGTTGGATGATGCTTTATACACCCCGTGC | sf000132_AG_GA_Pa | GGGAAATTGGGGATTGTGTTTACTTTG |
| sequenom | SEQ017 | sf000364_AG | sf000364_AG_GA_F | ACGTTGGATGCTTGCTCTTGTGATGAGATG | sf000364_AG_GA_R | ACGTTGGATGTCTGCTTCTAGCTCCTATGC | sf000364_AG_GA_Pa | CCATTACTAGCTCTTTCCTA |
| sequenom | SEQ017 | sf000505_CT | sf000505_CT_CT_F | ACGTTGGATGAACCGGACGATTCCATTCAG | sf000505_CT_CT_R | ACGTTGGATGTGTTTCACTCCACGCTGTTG | sf000505_CT_CT_P | CTTTCAGGATTGTCGTCTA |
| sequenom | SEQ017 | sf000612_AT | sf000612_AT_AT_F | ACGTTGGATGCCGACAGAGACTTCTCAAAC | sf000612_AT_AT_R | ACGTTGGATGGCAAAATGTCTAGTTGTAATG | sf000612_AT_AT_P | AAAATGTCTAGTTGTAATGACGTCA |
| sequenom | SEQ017 | sf000754_AC | sf000754_AC_CA_F | ACGTTGGATGCAGGTTTGTGTGTTTCACCG | sf000754_AC_CA_R | ACGTTGGATGGAGGGAATAACTCTCATTCTG | sf000754_AC_CA_Pa | AACTCTCATTCTGACATTTTAC |
| sequenom | SEQ017 | sf000899_CT | sf000899_CT_CT_F | ACGTTGGATGACCCTGATGTGCCTTATTGC | sf000899_CT_CT_R | ACGTTGGATGTGGCTGTGTCTTGAATGCTG | sf000899_CT_CT_P | ACATGATATACCACGGGTA |
| sequenom | SEQ017 | sf000991_AT | sf000991_AT_TA_F | ACGTTGGATGGGGAATAGTCTCATAATAGGC | sf000991_AT_TA_R | ACGTTGGATGGACTCTTACACGAATAGAAC | sf000991_AT_TA_Pa | TGGATATTACATGACTATTACATGACTATT |
| sequenom | SEQ017 | sf001353_GT | sf001353_GT_GT_F | ACGTTGGATGGAGAGTCTGAGGAAGTGTTG | sf001353_GT_GT_R | ACGTTGGATGATGGACCATAGAGCATGGAG | sf001353_GT_GT_Pa | GGCATGGAGACTCTCCTC |
| sequenom | SEQ017 | sf003142_AG | sf003142_AG_GA_F | ACGTTGGATGTCTCTGATCAAATCTTCTC | sf003142_AG_GA_R | ACGTTGGATGCGAACTTCCATTTAGCATAG | sf003142_AG_GA_Pa | CAATTTTATATGTACATACTATCTGAT |
| sequenom | SEQ017 | sf003899_CT | sf003899_CT_TC_F | ACGTTGGATGGCACAATGTCCAACAATTCC | sf003899_CT_TC_R | ACGTTGGATGATGTCACCCTATGACTTTGG | sf003899_CT_TC_Pa | ACCCTATGACTTTGGAAACAT |
| sequenom | SEQ017 | sf004211_GT | sf004211_GT_GT_F | ACGTTGGATGGCCACTAGCACCAATTATAC | sf004211_GT_GT_R | ACGTTGGATGCTGGTTCAGAATGCTAAGCC | sf004211_GT_GT_Pa | CCTAAAATGCTAATATACGCTAACA |
| sequenom | SEQ017 | sf004254_CT | sf004254_CT_CT_F | ACGTTGGATGCGTTTCTCACTTGGAAACAG | sf004254_CT_CT_R | ACGTTGGATGAGCTCAAGGTTGCTCAACTG | sf004254_CT_CT_P | TAGGGAAGAGAGATGTACT |
| sequenom | SEQ017 | sf004319_GT | sf004319_GT_GT_F | ACGTTGGATGTATGGGACAGGAAGTCACAG | sf004319_GT_GT_R | ACGTTGGATGGGCTTGCAAATCCTAAGTGG | sf004319_GT_GT_Pa | AAGTTTGGTTAAATGGAAATAAACAT |
| sequenom | SEQ017 | sf004353_AG | sf004353_AG_GA_F | ACGTTGGATGGGCTCAATAGCAACACACAG | sf004353_AG_GA_R | ACGTTGGATGCCTGATTAATGCCAGATCTT | sf004353_AG_GA_Pa | ACAAAACATGAGCAGC |
| sequenom | SEQ017 | sf004373_AG | sf004373_AG_GA_F | ACGTTGGATGACGCTTAGATAAGTCCCCTG | sf004373_AG_GA_R | ACGTTGGATGTGAGGTGACAAGGGATACAG | sf004373_AG_GA_Pa | CCTATTGATTTTTATGGGTGACCTAAC |
| sequenom | SEQ017 | sf004560_GT | sf004560_GT_GT_F | ACGTTGGATGGTGCTGAATTCCTGAAGAAC | sf004560_GT_GT_R | ACGTTGGATGAGATGGGTGAGGGATATTTG | sf004560_GT_GT_P | GAATTGAGCATGGCAGC |
| sequenom | SEQ017 | sf004583_AC | sf004583_AC_CA_F | ACGTTGGATGAGCAGTGGGCTAATCCAAAG | sf004583_AC_CA_R | ACGTTGGATGGAGTAAGGAAACCCATAGCC | sf004583_AC_CA_Pa | GCTAACGTTACTTACTTTTAAAT |
| sequenom | SEQ017 | sf004624_CT | sf004624_CT_CT_F | ACGTTGGATGGAACCAGTTTGCTACAGCAC | sf004624_CT_CT_R | ACGTTGGATGCACTAAGGTCACTTGCAAAC | sf004624_CT_CT_P | TCCTGTTGTTGCATGAT |
| sequenom | SEQ017 | sf004651_AG | sf004651_AG_AG_F | ACGTTGGATGAAAGGGTCTCTGTGATGGTG | sf004651_AG_AG_R | ACGTTGGATGCGAGAGAGAGAAACAACACG | sf004651_AG_AG_P | CGTTATACAAGAAAACAACAGATC |
| sequenom | SEQ017 | sf004685_AGa | sf004685_AGa_AG_F | ACGTTGGATGCTCAGACAGATCCACTGAAC | sf004685_AGa_AG_R | ACGTTGGATGCACTAACAAAGCCAGTGTGC | sf004685_AGa_AG_P | GTGTGCAGTAGGGCGG |
| sequenom | SEQ017 | sf004685_CG | sf004685_CG_GC_F | ACGTTGGATGCACTAACAAAGCCAGTGTGC | sf004685_CG_GC_R | ACGTTGGATGCTCAGACAGATCCACTGAAC | sf004685_CG_GC_Pa | GGGGTACTAGGCGATGATGCAT |
| sequenom | SEQ017 | sf004839_CT | sf004839_CT_CT_F | ACGTTGGATGCGTGCTTTTTCTCCAAACCC | sf004839_CT_CT_R | ACGTTGGATGTGTCCAAAAGTGCTTGGCAG | sf004839_CT_CT_P | GGCTTGGCAGGTCATATTTGATAA |
| sequenom | SEQ017 | sf004938_CT | sf004938_CT_TC_F | ACGTTGGATGCCAATTATGCTTTGCTTTTG | sf004938_CT_TC_R | ACGTTGGATGCTCAACTGTGAATGGTCCTG | sf004938_CT_TC_Pa | AGTTTGGATTTGGCTTCACGTTAATCGT |
| sequenom | SEQ017 | sf005311_CG | sf005311_CG_CG_F | ACGTTGGATGGTTACTTGTGTAGTGGAGTC | sf005311_CG_CG_R | ACGTTGGATGTGGTTAGCTTCACCTACCTG | sf005311_CG_CG_P | GCATGGTAGTAACATGAGTTG |
| sequenom | SEQ017 | sf005440_AG | sf005440_AG_GA_F | ACGTTGGATGTGTGTCTGTTCATGTTGTGG | sf005440_AG_GA_R | ACGTTGGATGAGCTGTAACTTCTCAGCTAC | sf005440_AG_GA_Pa | TCTGTAACTTCTCAGCTACATATTG |
| sequenom | SEQ018 | sf000020_CT | sf000020_CT_TC_F | ACGTTGGATGGAGAAGGCCCTAATGCAATG | sf000020_CT_TC_R | ACGTTGGATGGCGATCTCTGTTCCTAATGC | sf000020_CT_TC_Pa | AGGATGCAACTGACCTGGC |

| | | | | | | | | |
|---|---|---|---|---|---|---|---|---|
| sequenom | SEQ018 | sf000062_CG | sf000062_CG_CG_F | ACGTTGGATGGGGTAGTAGTCTGGGTAAAG | sf000062_CG_CG_R | ACGTTGGATGCCTTGGAACAAGGATGGAAC | sf000062_CG_CG_P | ACGCCACTGAAACACACA |
| sequenom | SEQ018 | sf000151_AT | sf000151_AT_TA_F | ACGTTGGATGTTATCATGTGAGGCTCAGGG | sf000151_AT_TA_R | ACGTTGGATGGACCTAATAGTCAACGGGAG | sf000151_AT_TA_Pa | GACGGGAGTACTCATGATGGCACA |
| sequenom | SEQ018 | sf000310_AC | sf000310_AC_CA_F | ACGTTGGATGCTGTGGCTAAACATGTTAAGT | sf000310_AC_CA_R | ACGTTGGATGGGGAAGAGTACAAACAGAGC | sf000310_AC_CA_P | CCTGAAGAGTACAAACAGAGCTGGGAC |
| sequenom | SEQ018 | sf000364_GT | sf000364_GT_GT_F | ACGTTGGATGTTGTAAAATGGCGTCGACAG | sf000364_GT_GT_R | ACGTTGGATGGGAAAGATCTGTTCTTGCTC | sf000364_GT_GT_P | AGGAAAGAGCTAGTAATGG |
| sequenom | SEQ018 | sf001287_CT | sf001287_CT_TC_F | ACGTTGGATGCTGTCTATATATCGACCAGG | sf001287_CT_TC_R | ACGTTGGATGGAGACTTTAAAATCTCAAG | sf001287_CT_TC_Pa | GCTCGATATTCAATTAAGAAGCG |
| sequenom | SEQ018 | sf001394_AT | sf001394_AT_TA_F | ACGTTGGATGAATGGGATGGGCTCTTAGTG | sf001394_AT_TA_R | ACGTTGGATGGCTATGACCTTCATGCAATG | sf001394_AT_TA_Pa | AGAGTAAATGAGGTGAGAGATTA |
| sequenom | SEQ018 | sf001546_AC | sf001546_AC_CA_F | ACGTTGGATGTACGAGGTGTAGCTGTGATG | sf001546_AC_CA_R | ACGTTGGATGGGCTGTCAACAGACAAGTAG | sf001546_AC_CA_P | AAGTAGTCACAAACGCAACCACACACA |
| sequenom | SEQ018 | sf001846_AG | sf001846_AG_AG_F | ACGTTGGATGCACACTAGATATGGATCACG | sf001846_AG_AG_R | ACGTTGGATGATGCTTATCGTTCCACCACC | sf001846_AG_AG_P | CCGTCGGCTTTAATATTTGACTT |
| sequenom | SEQ018 | sf002055_AG | sf002055_AG_GA_F | ACGTTGGATGGACTGATGCATTGTCACCTG | sf002055_AG_GA_R | ACGTTGGATGTAAACCCCAGTTTGCTGTCG | sf002055_AG_GA_Pa | CCACCACTGAATCAGACAGTG |
| sequenom | SEQ018 | sf002721_GT | sf002721_GT_GT_F | ACGTTGGATGATTAGTGATGATGGGACTGC | sf002721_GT_GT_R | ACGTTGGATGGCCTCTGGAAAAACACACTG | sf002721_GT_GT_Pa | ACTCTGGAAAAACACACTGACTATATGAA |
| sequenom | SEQ018 | sf003016_AG | sf003016_AG_GA_F | ACGTTGGATGCCTCTATTTACAGAGGGAAG | sf003016_AG_GA_R | ACGTTGGATGGACCAAAGTCCCACAAACAC | sf003016_AG_GA_Pa | CCCCCAGCACATAACTACAGATCTTTGTC |
| sequenom | SEQ018 | sf003382_AG | sf003382_AG_GA_F | ACGTTGGATGGCACTACTTTTGACCAGAGC | sf003382_AG_GA_R | ACGTTGGATGTCTAGCACAACGTCATAAGC | sf003382_AG_GA_Pa | AACCCTAATCCCTGTATAGTG |
| sequenom | SEQ018 | sf003611_AC | sf003611_AC_CA_F | ACGTTGGATGGACATAGCTCAACATGTCTG | sf003611_AC_CA_R | ACGTTGGATGGCTGCAGGAGAATCTAAATG | sf003611_AC_CA_P | TGTCGATGTACCTTTATCCAGATAA |
| sequenom | SEQ018 | sf003639_CT | sf003639_CT_CT_F | ACGTTGGATGCCGTTGTTATTTCGAGCGTG | sf003639_CT_CT_R | ACGTTGGATGAACAAACCTTTAATCCACC | sf003639_CT_CT_P | ACAAACCTTTAATCCACCATGTAACC |
| sequenom | SEQ018 | sf003755_CT | sf003755_CT_TC_F | ACGTTGGATGGCAGGAGTACTTTAAATTGTG | sf003755_CT_TC_R | ACGTTGGATGCTGCAGCAACAGAACATGTG | sf003755_CT_TC_Pa | TGTGAATTAAGTGGATTATAATTAAATAAC |
| sequenom | SEQ018 | sf003922_GT | sf003922_GT_GT_F | ACGTTGGATGCCACAATGTAAGACATGCAC | sf003922_GT_GT_R | ACGTTGGATGCCTTTCCTGGGTAGCTTATC | sf003922_GT_GT_P | CAAGGATTTGTGATCACATTCTCA |
| sequenom | SEQ018 | sf003974_GT | sf003974_GT_GT_F | ACGTTGGATGTCTTTGTTCCCTACCCTTGC | sf003974_GT_GT_R | ACGTTGGATGGGTAGTCACTCTGCTCTTTC | sf003974_GT_GT_Pa | ATCTTTCCTGAAGTCCATGATC |
| sequenom | SEQ018 | sf004008_AG | sf004008_AG_AG_F | ACGTTGGATGTCTTCAGTTCCCAGTTTAGC | sf004008_AG_AG_R | ACGTTGGATGCTAGCTGGATAAAACATGGC | sf004008_AG_AG_P | AGGATTGAATTCCATGAGACTTCTT |
| sequenom | SEQ018 | sf004045_CT | sf004045_CT_CT_F | ACGTTGGATGTGTCTCTGTGTGATGCTGTG | sf004045_CT_CT_R | ACGTTGGATGCGGATGACATTTTCCACCTG | sf004045_CT_CT_P | GAGGAAAAACACCAACAGAAAAACAC |
| sequenom | SEQ018 | sf004434_AT | sf004434_AT_CG_F | ACGTTGGATGTGCAAGATATGGGCTACTAC | sf004434_AT_CG_R | ACGTTGGATGCGAGTGGAAATCCAATAGCC | sf004434_AT_CG_P | GGTCTTTGCTTTTTAGACC |
| sequenom | SEQ018 | sf004471_AG | sf004471_AG_GA_F | ACGTTGGATGGATTGCCCAGTGACATTGTG | sf004471_AG_GA_R | ACGTTGGATGGACTCCATCTTCTGGTTGGC | sf004471_AG_GA_Pa | TGGTTGGCTCTCTCCGC |
| sequenom | SEQ018 | sf004521_CG | sf004521_CG_CG02_F | ACGTTGGATGTCACTTAATGGACTCCAGGC | sf004521_CG_CG02_R | ACGTTGGATGGTGTCAGGAGAAACGTTAC | sf004521_CG_CG02_P | ACATATAGTGATATAAATGATTTTGTATA |
| sequenom | SEQ018 | sf004656_AC | sf004656_AC_CA_F | ACGTTGGATGATGTTTCGAAAGCAGGCTCC | sf004656_AC_CA_R | ACGTTGGATGTATCTACTGCCCGAGAAGTG | sf004656_AC_CA_Pa | GGGTTTGCTTATTAAAAGAGACATAAA |
| sequenom | SEQ018 | sf004668_AC | sf004668_AC_CA_F | ACGTTGGATGTCACTGAGATAGCCAAGGAG | sf004668_AC_CA_R | ACGTTGGATGTCCCAGAAGTGGTTAGATTC | sf004668_AC_CA_P | AGTTAGCTGGTTTCTGACGT |
| sequenom | SEQ018 | sf004712_CT | sf004712_CT_CT_F | ACGTTGGATGTCCACCTTGGCTTATTGCAC | sf004712_CT_CT_R | ACGTTGGATGTGCAGTTATCTTAGCCAGCC | sf004712_CT_CT_P | TTGCAGCTAACAATTAGCTAAATGACAT |
| sequenom | SEQ018 | sf004715_AC | sf004715_AC_CA_F | ACGTTGGATGACTGTACTAGTGCACGTGAC | sf004715_AC_CA_R | ACGTTGGATGGGGTTTGGTAATGTCCCAC | sf004715_AC_CA_Pa | TGTCCCACACCCAACC |
| sequenom | SEQ018 | sf004741_CG | sf004741_CG_CG_F | ACGTTGGATGAGATGACAGGAAATGGAGCG | sf004741_CG_CG_R | ACGTTGGATGCAGTCAATTTGCAGAGCAGG | sf004741_CG_CG_P | GTTGTTCTAGATGAAGGC |
| sequenom | SEQ018 | sf004774_AG | sf004774_AG_AG_F | ACGTTGGATGAACCTGCAGCCTGATCAAAG | sf004774_AG_AG_R | ACGTTGGATGTGGCAATGGTATGGCACATC | sf004774_AG_AG_P | TTGGGTACGTAGCACAAGCATCTAGA |

| | | | | | | | | |
|---|---|---|---|---|---|---|---|---|
| sequenom | SEQ018 | sf005157_CT | sf005157_CT_CT_F | ACGTTGGATGGAACATCATTGTTGGAGCAG | sf005157_CT_CT_R | ACGTTGGATGCTTTTAAAATGGGCCTTTGC | sf005157_CT_CT_P | GATTAGAGAAACTCATGAAACA |
| sequenom | SEQ018 | sf005160_AC | sf005160_AC_CA_F | ACGTTGGATGCTTGAAGGCTCTTACGTTGG | sf005160_AC_CA_R | ACGTTGGATGGTTGAGGTCAGAAGTAGAAG | sf005160_AC_CA_Pa | ATCAGATGGAGTTGAAGACGA |
| sequenom | SEQ018 | sf006410_AG | sf006410_AG_GA02_F | ACGTTGGATGGCTTCAATTGGCTCCATATC | sf006410_AG_GA02_R | ACGTTGGATGTGTAAACTCAAGACCCTCAC | sf006410_AG_GA02_Pa | AAACCATGTTCTATGATTTCTAATATTT |

**Table S3 Description and annotation of the 281 SNP (single nucleotide-polymorphism) markers used to build the linkage map in the Brook charr, *Salvelinus fontinalis*.**

**Ts: transition; Tv: transversion; C: coding region; NC: non coding region; S: synonymous; NS: non synonymous; LG: linkage group.**

| | SNP Name | Accession number | GenInfo Identifiers (GI) | Variation | Ts/Tv | C/NC | S/NS | LG |
|---|---|---|---|---|---|---|---|---|
| 1 | sf000002 | BT050259 | GI:209738381 | A/G | Ts | NC | - | 22 |
| 2 | sf000017 | XP_691985.3 | GI:189515371 | C/G | Tv | C | - | 31 |
| 3 | sf000020 | | | C/T | Ts | C | NS | 35 |
| 4 | sf000021 | BT044685.1 | GI:209148638 | A/C | Tv | C | - | 14 |
| 5 | sf000023 | | | C/T | Ts | NC | - | - |
| 6 | sf000032 | | | A/G | Ts | C | - | 15 |
| 7 | sf000062 | | | C/G | Tv | NC | - | - |
| 8 | sf000071 | | | A/C | Tv | NC | - | 3 |
| 9 | sf000078 | | | A/T | Tv | NC | - | 27 |
| 10 | sf000086 | XM_001921499.1 | GI:189516301 | C/T | Ts | NC | - | - |
| 11 | sf000088 | XP_002609992.1 | GI:260830085 | A/G | Ts | NC | - | - |
| 12 | sf000119 | | | A/G | Ts | NC | - | 12 |
| 13 | sf000124 | | | A/G | Ts | C | - | 17 |
| 14 | sf000132 | | | A/G | Ts | NC | - | 13 |
| 15 | sf000137 | NM_200955 | GI:41055673 | A/T | Tv | C | NS | - |
| 16 | sf000139 | | | G/T | Tv | NC | - | 4 |
| 17 | sf000151 | | | A/T | Tv | NC | - | 15 |
| 18 | sf000157 | NP_775368 | GI:27545272 | A/C | Tv | C | NS | 25 |
| 19 | sf000175 | XP_001054847 | GI:109498064 | G/T | Tv | NC | - | - |
| 20 | sf000178 | | | A/G | Ts | C | - | 19 |
| 21 | sf000179 | | | C/T | Ts | NC | - | 3 |
| 22 | sf000191 | | | C/G | Tv | NC | - | - |

| | | | | | | | | |
|---|---|---|---|---|---|---|---|---|
| 23 | sf000209 | | | C/T | Ts | NC | - | - |
| 24 | sf000228 | | | A/T | Tv | NC | - | - |
| 25 | sf000310 | | | A/T | Tv | NC | - | 10 |
| 26 | sf000324 | | | A/C | Tv | NC | - | 8 |
| 27 | sf000327 | | | A/G | Ts | NC | - | - |
| 28 | sf000364 | | | A/G | Ts | C | NS | 31 |
| 29 | sf000364 | | | G/T | Tv | C | NS | 31 |
| 30 | sf000382 | | | A/G | Ts | NC | - | - |
| 31 | sf000390 | BT057320 | GI:221221062 | C/T | Ts | NC | - | - |
| 32 | sf000392 | CAAE01015004.1 | GI:47226310 | C/T | Ts | C | - | 23 |
| 33 | sf000397 | | | C/G | Tv | NC | - | 20 |
| 34 | sf000428 | | | A/T | Tv | NC | - | 3 |
| 35 | sf000505 | | | C/T | Ts | C | - | 1 |
| 36 | sf000508 | | | C/T | Ts | NC | - | 4 |
| 37 | sf000518 | | | G/T | Tv | NC | - | - |
| 38 | sf000554 | BT059059.1 | GI:223648027 | C/T | Ts | C | NS | 2 |
| 39 | sf000559 | XM_001502487 | GI:149728067 | A/G | Ts | NC | - | - |
| 40 | sf000583 | EF467299.1 | GI:154795566 | C/T | Ts | NC | - | 8 |
| 41 | sf000584 | EU025714 | GI:158702319 | A/G | Ts | C | - | 10 |
| 42 | sf000612 | NM_001020538 | GI:66472671 | A/T | Tv | NC | - | 37 |
| 43 | sf000633 | | | G/T | Tv | C | - | - |
| 44 | sf000656 | | | A/G | Ts | NC | - | 20 |
| 45 | sf000657 | | | C/T | Ts | NC | - | 2 |
| 46 | sf000679 | BT060419 | GI:223673194 | C/T | Ts | NC | - | - |
| 47 | sf000684 | GU129140 | GI:213512874 | C/T | Ts | C | - | 34 |
| 48 | sf000706 | NP_001117688 | GI:185135493 | A/G | Ts | NC | - | 6 |
| 49 | sf000717 | | | C/T | Ts | NC | - | 18 |
| 50 | sf000719 | | | A/T | Tv | NC | - | 16 |

| | | | | | | | | |
|---|---|---|---|---|---|---|---|---|
| 51 | sf000744 | | | A/T | Tv | C | NS | 5 |
| 52 | sf000747 | | | C/T | Ts | NC | - | - |
| 53 | sf000754 | | | A/C | Tv | NC | - | 8 |
| 54 | sf000810 | BT045174 | GI:209154407 | C/T | Ts | NC | - | 2 |
| 55 | sf000854 | | | G/T | Tv | NC | - | - |
| 56 | sf000878 | | | C/T | Ts | NC | - | - |
| 57 | sf000891 | | | A/C | Tv | C | NS | 6 |
| 58 | sf000899 | EU025705 | GI:158702261 | C/T | Ts | NC | - | 12 |
| 59 | sf000905 | | | A/T | Tv | C | - | 31 |
| 60 | sf000961 | | | C/T | Ts | NC | - | 5 |
| 61 | sf000966 | | | A/G | Ts | NC | - | 13 |
| 62 | sf000970 | | | A/G | Ts | C | - | 9 |
| 63 | sf000978 | | | A/G | Ts | NC | - | 1 |
| 64 | sf000991 | | | A/T | Tv | C | - | - |
| 65 | sf001069 | | | G/T | Tv | NC | - | - |
| 66 | sf001086 | NM_001141083 | GI:213513628 | C/T | Ts | NC | - | - |
| 67 | sf001105 | | | A/G | Ts | NC | - | 17 |
| 68 | sf001164 | | | C/T | Ts | NC | - | - |
| 69 | sf001181 | | | A/G | Ts | C | - | - |
| 70 | sf001184 | CR391965 | GI:167908776 | C/T | Ts | NC | - | 1 |
| 71 | sf001196 | | | A/G | Ts | NC | - | - |
| 72 | sf001284 | | | G/T | Tv | NC | - | 33 |
| 73 | sf001287 | | | C/T | Ts | C | - | - |
| 74 | sf001288 | | | C/T | Ts | C | NS | 2 |
| 75 | sf001353 | | | G/T | Tv | C | - | 5 |
| 76 | sf001359 | GQ505860 | GI:260182183 | A/G | Ts | NC | - | - |
| 77 | sf001394 | | | A/T | Tv | NC | - | 6 |
| 78 | sf001437 | | | A/G | Ts | NC | - | - |

| | | | | | | | | |
|---|---|---|---|---|---|---|---|---|
| 79 | sf001546 | | | A/C | Tv | C | NS | 28 |
| 80 | sf001601 | | | C/T | Ts | NC | - | 8 |
| 81 | sf001644 | | | G/T | Tv | C | - | 2 |
| 82 | sf001661 | | | G/T | Tv | C | - | - |
| 83 | sf001667 | | | A/G | Ts | C | NS | 17 |
| 84 | sf001686 | | | A/G | Ts | NC | - | - |
| 85 | sf001692 | | | C/T | Ts | NC | - | 12 |
| 86 | sf001740 | | | A/G | Ts | NC | - | 7 |
| 87 | sf001770 | | | A/G | Ts | NC | - | 21 |
| 88 | sf001812 | AC203446 | GI:163955243 | A/G | Ts | NC | - | 9 |
| 89 | sf001831 | | | A/C | Tv | NC | - | 40 |
| 90 | sf001846 | | | A/G | Ts | NC | - | 30 |
| 91 | sf001880 | EF467300 | GI:154795567 | C/T | Ts | NC | - | - |
| 92 | sf001996 | DQ246664 | GI:80971723 | A/G | Ts | NC | - | 28 |
| 93 | sf002018 | | | A/C | Tv | NC | - | 25 |
| 94 | sf002024 | | | A/G | Ts | NC | - | 3 |
| 95 | sf002033 | AJ311906 | GI:20873289 | A/T | Tv | C | - | 2 |
| 96 | sf002055 | | | A/G | Ts | C | NS | 31 |
| 97 | sf002131 | | | A/G | Ts | C | - | 1 |
| 98 | sf002145 | XM_683887 | GI:292622326 | C/G | Tv | C | NS | 31 |
| 99 | sf002155 | | | A/G | Ts | NC | - | - |
| 100 | sf002175 | | | C/T | Ts | NC | - | 34 |
| 101 | sf002203 | | | A/G | Ts | C | - | 7 |
| 102 | sf002250 | XM_001920907 | GI:189520811 | A/C | Tv | C | - | 28 |
| 103 | sf002333 | | | A/G | Ts | NC | - | - |
| 104 | sf002353 | NM_001141843 | GI:213513502 | C/T | Ts | NC | - | 20 |
| 105 | sf002439 | | | C/T | Ts | NC | - | 31 |
| 106 | sf002633 | BT072804 | GI:224587929 | A/C | Tv | C | - | - |

| | | | | | | | | |
|---|---|---|---|---|---|---|---|---|
| 107 | sf002640 | | | A/G | Ts | NC | - | 21 |
| 109 | sf002721 | | | A/G | Ts | C | - | 32 |
| 110 | sf002948 | CAAE01015039 | GI:47222189 | G/T | Tv | C | S | 7 |
| 111 | sf003018 | | | A/G | Ts | NC | - | 26 |
| 112 | sf003063 | | | A/C | Tv | NC | - | 1 |
| 113 | sf003076 | EU025709 | GI:158702304 | A/G | Ts | NC | - | 23 |
| 114 | sf003084 | | | A/G | Ts | C | - | 4 |
| 115 | sf003092 | | | C/T | Ts | C | - | 9 |
| 116 | sf003105 | | | C/T | Ts | NC | - | 3 |
| 117 | sf003118 | | | A/G | Ts | NC | - | 6 |
| 118 | sf003141 | BT056462 | GI:221219345 | A/T | Tv | NC | - | 11 |
| 119 | sf003142 | CU062627 | GI:150171078 | A/G | Ts | NC | - | - |
| 120 | sf003197 | | | A/G | Ts | NC | - | - |
| 121 | sf003208 | | | C/T | Ts | NC | - | - |
| 122 | sf003334 | | | A/G | Ts | NC | - | 24 |
| 123 | sf003382 | | | A/G | Ts | C | NS | 14 |
| 124 | sf003407 | | | C/T | Ts | NC | - | 10 |
| 125 | sf003442 | | | A/G | Ts | C | - | 24 |
| 126 | sf003455 | | | A/G | Ts | NC | - | 11 |
| 127 | sf003500 | BT049606.1 | GI:209737075 | C/T | Ts | NC | - | 1 |
| 128 | sf003520 | | | A/G | Ts | C | S | 2 |
| 129 | sf003538 | AL840630 | GI:41322894 | G/T | Tv | NC | - | 9 |
| 130 | sf003560 | | | C/T | Ts | C | S | - |
| 131 | sf003589 | | | C/T | Ts | C | - | - |
| 132 | sf003595 | | | A/T | Tv | NC | - | - |
| 133 | sf003601 | | | C/T | Ts | NC | - | - |
| 134 | sf003610 | | | A/T | Tv | C | - | 1 |
| 135 | sf003611 | | | A/C | Tv | NC | - | - |

| | | | | | | | | |
|---|---|---|---|---|---|---|---|---|
| 136 | sf003614 | CAAE01014563 | GI:47223303 | G/T | Tv | NC | - | 8 |
| 137 | sf003621 | BT059052 | GI:223648013 | C/T | Ts | NC | - | - |
| 138 | sf003633 | | | A/G | Ts | C | S | 21 |
| 139 | sf003639 | EF427381 | GI:148362130 | C/T | Ts | NC | - | 30 |
| 140 | sf003647 | | | C/G | Tv | NC | - | 40 |
| 141 | sf003697 | | | A/C | Tv | NC | - | 37 |
| 142 | sf003698 | | | G/T | Tv | C | - | - |
| 143 | sf003700 | | | C/T | Ts | NC | - | 9 |
| 144 | sf003713 | XM_686639.3 | GI:189530935 | A/T | Tv | NC | - | 25 |
| 145 | sf003715 | EU816603 | GI:209962458 | C/T | Ts | NC | - | 11 |
| 146 | sf003724 | | | C/T | Ts | NC | - | - |
| 147 | sf003728 | | | C/T | Ts | C | - | 31 |
| 148 | sf003755 | | | G/T | Tv | NC | - | 7 |
| 149 | sf003788 | XM_002660927 | GI:292611089 | G/T | Tv | NC | - | - |
| 150 | sf003813 | EU621898 | GI:187936476 | A/G | Ts | NC | - | 8 |
| 151 | sf003836 | | | A/T | Tv | NC | - | 15 |
| 152 | sf003897 | | | A/G | Ts | NC | - | 10 |
| 153 | sf003899 | | | C/T | Ts | NC | - | 11 |
| 154 | sf003919 | GQ505860 | GI:260182183 | C/T | Ts | NC | - | 1 |
| 155 | sf003977 | EU481821 | GI:171474994 | C/G | Tv | NC | - | - |
| 156 | sf003994 | | | G/T | Tv | NC | - | - |
| 157 | sf004008 | | | A/G | Ts | C | NS | - |
| 158 | sf004013 | EU221180 | GI:159033051 | A/C | Tv | C | NS | 38 |
| 159 | sf004016 | | | A/G | Ts | NC | - | 2 |
| 160 | sf004030 | XM_689729 | GI:189528958 | A/T | Tv | NC | - | 19 |
| 161 | sf004038 | NM_001165151 | GI:259089170 | A/G | Ts | NC | - | 34 |
| 162 | sf004055 | XP_001921123 | GI:189530039 | A/T | Tv | C | NS | 22 |
| 163 | sf004056 | EF467297 | GI:154795564 | C/T | Ts | C | NS | - |

| | | | | | | | | |
|---|---|---|---|---|---|---|---|---|
| 164 | sf004064 | | | A/C | Tv | NC | - | 28 |
| 165 | sf004070 | | | A/G | Ts | NC | - | 24 |
| 166 | sf004090 | | | C/G | Tv | C | NS | - |
| 167 | sf004099 | | | A/T | Tv | NC | - | - |
| 168 | sf004112 | | | A/G | Ts | NC | - | - |
| 169 | sf004131 | NM_001173900 | GI:291190431 | A/G | Ts | NC | - | 40 |
| 170 | sf004149 | FJ969490 | GI:262189352 | C/G | Tv | NC | - | 6 |
| 171 | sf004155 | EU025709 | GI:158702304 | G/T | Tv | NC | - | - |
| 172 | sf004192 | | | A/G | Ts | C | - | - |
| 173 | sf004209 | | | A/T | Tv | NC | - | - |
| 174 | sf004211 | | | G/T | Tv | NC | - | 3 |
| 175 | sf004214 | | | A/G | Ts | NC | - | 6 |
| 176 | sf004240 | EU621900 | GI:187936480 | A/G | Ts | NC | - | 6 |
| 177 | sf004252 | NM_001140784 | GI:213515209 | G/T | Tv | NC | - | 5 |
| 178 | sf004254 | BT071904 | GI:224613219 | C/T | Ts | C | - | 13 |
| 179 | sf004257 | AB294121 | GI:149773365 | A/G | Ts | NC | - | 3 |
| 180 | sf004260 | | | C/T | Ts | C | - | - |
| 181 | sf004288 | | | A/G | Ts | C | - | - |
| 182 | sf004299 | XM_001922165 | GI:189531595 | C/T | Ts | NC | - | 10 |
| 183 | sf004319 | BT058994 | GI:223647897 | G/T | Tv | C | - | 23 |
| 184 | sf004353 | NM_001124574 | GI:185135642 | A/G | Ts | C | S | 40 |
| 185 | sf004357 | EU025708 | GI:158702285 | C/T | Ts | C | NS | 4 |
| 186 | sf004373 | | | A/G | Ts | C | - | 5 |
| 187 | sf004379 | | | C/T | Ts | NC | - | 15 |
| 188 | sf004387 | | | C/T | Ts | C | - | - |
| 189 | sf004416 | GU129140 | GI:290760588 | A/C | Tv | NC | - | 31 |
| 190 | sf004423 | | | A/G | Ts | C | - | - |
| 191 | sf004426 | | | C/T | Ts | NC | - | 24 |

| | | | | | | | | |
|---|---|---|---|---|---|---|---|---|
| 192 | sf004438 | BT045405 | GI:209154869 | C/T | Ts | C | NS | 31 |
| 193 | sf004475 | EU621899 | GI:187936478 | C/T | Ts | C | S | 25 |
| 194 | sf004521 | | | C/G | Tv | NC | - | 1 |
| 195 | sf004529 | | | A/T | Tv | NC | - | 38 |
| 196 | sf004541 | | | A/C | Tv | NC | - | 2 |
| 197 | sf004545 | EF427379 | GI:148362119 | A/C | Tv | NC | - | - |
| 198 | sf004560 | | | G/T | Tv | NC | - | 3 |
| 199 | sf004562 | | | A/C | Tv | NC | - | 7 |
| 200 | sf004583 | NM_001013274 | GI:61651703 | A/C | Tv | NC | - | - |
| 201 | sf004602 | XM_001922165 | GI:189531595 | A/G | Ts | NC | - | - |
| 202 | sf004614 | | | A/G | Ts | C | - | 13 |
| 203 | sf004624 | EU816603 | GI:209962458 | C/T | Ts | NC | - | 10 |
| 204 | sf004632 | EU025707 | GI:158702273 | C/T | Ts | NC | - | - |
| 205 | sf004642 | | | A/G | Ts | NC | - | 6 |
| 206 | sf004651 | | | A/G | Ts | C | NS | 6 |
| 207 | sf004656 | | | A/C | Tv | NC | - | 21 |
| 208 | sf004668 | FJ969489 | GI:262189345 | A/C | Tv | C | - | 38 |
| 209 | sf004684 | | | A/T | Tv | NC | - | - |
| 210 | sf004685 | | | A/G | Ts | NC | - | 39 |
| 211 | sf004685 | | | A/G | Ts | C | NS | 39 |
| 212 | sf004685 | | | C/G | Tv | C | NS | - |
| 213 | sf004714 | EU025714 | GI:158702319 | C/T | Ts | C | - | 27 |
| 214 | sf004715 | | | C/T | Ts | C | - | 10 |
| 215 | sf004732 | AB258536 | GI:118596560 | C/T | Ts | NC | - | 29 |
| 216 | sf004741 | | | C/G | Tv | NC | - | 31 |
| 217 | sf004762 | | | C/T | Ts | NC | - | 18 |
| 218 | sf004765 | | | C/T | Ts | C | NS | 4 |
| 219 | sf004774 | | | A/G | Ts | C | - | 3 |

| | | | | | | | | |
|---|---|---|---|---|---|---|---|---|
| 220 | sf004795 | BT057087 | GI:221220595 | C/T | Ts | NC | - | 10 |
| 221 | sf004811 | EU025709 | GI:158702304 | A/T | Tv | C | NS | 15 |
| 222 | sf004817 | EU025714 | GI:158702319 | C/G | Tv | NC | - | - |
| 223 | sf004818 | EU481821 | GI:171474994 | A/C | Tv | NC | - | 2 |
| 224 | sf004828 | AJ716203 | GI:52673235 | A/T | Tv | NC | - | - |
| 225 | sf004839 | | | C/T | Ts | C | - | 39 |
| 226 | sf004848 | BT058155.1 | GI:223646219 | A/T | Tv | NC | - | 17 |
| 227 | sf004870 | | | A/G | Ts | NC | - | 7 |
| 228 | sf004890 | FJ969489 | GI:262189345 | A/C | Tv | NC | - | - |
| 229 | sf004897 | | | A/T | Tv | NC | - | - |
| 230 | sf004904 | | | C/G | Tv | NC | - | - |
| 231 | sf004909 | EU481821 | GI:171474994 | A/T | Tv | C | NS | 13 |
| 232 | sf004913 | BT059404 | GI:223648717 | A/C | Tv | NC | - | 4 |
| 233 | sf004917 | BT072749 | GI:224587877 | C/T | Ts | NC | - | 12 |
| 234 | sf004938 | EU025708 | GI:158702285 | C/T | Ts | NC | - | 9 |
| 235 | sf004975 | EU221176 | GI:159147122 | C/T | Ts | NC | - | 31 |
| 236 | sf004977 | EU025707 | GI:158702273 | C/T | Ts | NC | - | - |
| 237 | sf004984 | NM_001140391 | GI:213511551 | A/G | Ts | C | NS | 5 |
| 238 | sf005000 | DQ246664 | GI:80971723 | C/T | Ts | C | NS | - |
| 239 | sf005052 | BT058930 | GI:223647769 | A/G | Ts | C | - | - |
| 240 | sf005054 | BT059612 | GI:223649133 | A/G | Ts | NC | - | 3 |
| 241 | sf005079 | | | A/C | Tv | NC | - | - |
| 242 | sf005104 | EU025714 | GI:158702319 | A/C | Tv | NC | - | - |
| 243 | sf005138 | NM_001145584 | GI:224496041 | A/C | Tv | NC | - | - |
| 244 | sf005160 | | | A/C | Tv | C | - | 31 |
| 245 | sf005168 | BX537340 | GI:51592271 | A/C | Tv | C | - | 2 |
| 246 | sf005182 | SSSAT48S1 | GI:501083 | A/G | Ts | NC | - | - |
| 247 | sf005182 | SSSAT48S1 | GI:501083 | A/G | Ts | NC | - | - |

| | | | | | | | | |
|---|---|---|---|---|---|---|---|---|
| 248 | sf005186 | | | C/T | Ts | NC | - | 39 |
| 249 | sf005248 | EU481821 | GI:171474994 | C/T | Ts | NC | - | 9 |
| 250 | sf005258 | EU221180 | GI:159033051 | A/C | Tv | NC | - | - |
| 251 | sf005272 | EF467298 | GI:154795565 | C/G | Tv | NC | - | - |
| 252 | sf005286 | | | C/T | Ts | NC | - | - |
| 253 | sf005311 | GQ505859 | GI:260182171 | C/G | Tv | NC | - | - |
| 254 | sf005344 | GU129140 | GI:290760588 | A/T | Tv | C | NS | 32 |
| 255 | sf005399 | AM748538 | GI:242098051 | A/T | Tv | C | NS | - |
| 256 | sf005409 | CR847272 | GI:55058237 | A/C | Tv | C | NS | 8 |
| 257 | sf005410 | FJ969489 | GI:262189345 | A/C | Tv | NC | - | - |
| 258 | sf005420 | | | A/G | Ts | NC | - | 38 |
| 259 | sf005440 | | | C/T | Ts | NC | - | 21 |
| 260 | sf005440 | | | A/G | Ts | C | S | - |
| 261 | sf005443 | | | C/G | Tv | C | - | 37 |
| 262 | sf005459 | | | A/C | Tv | NC | - | 1 |
| 263 | sf005501 | BT072704 | GI:224587835 | A/G | Ts | NC | - | 9 |
| 264 | sf005540 | EU816603 | GI:209962458 | C/T | Ts | C | - | 19 |
| 265 | sf005590 | | | C/T | Ts | C | NS | 35 |
| 266 | sf005652 | | | A/G | Ts | NC | - | 8 |
| 267 | sf005654 | NM_001165392 | GI:259155245 | C/T | Ts | C | - | - |
| 268 | sf005663 | | | A/G | Ts | NC | - | 1 |
| 269 | sf005695 | | | C/T | Ts | C | - | - |
| 270 | sf005769 | NM_001140223 | GI:213511597 | A/G | Ts | NC | - | 14 |
| 271 | sf005769 | NM_001140223 | GI:213511597 | C/T | Ts | NC | - | 14 |
| 272 | sf005917 | EU221180 | GI:159033051 | C/T | Ts | NC | - | - |
| 273 | sf006000 | EU621898 | GI:187936476 | C/T | Ts | C | - | 35 |
| 274 | sf006000 | EU621898 | GI:187936476 | A/G | Ts | NC | - | - |
| 275 | sf006057 | XM_001919059 | GI:189519277 | A/G | Ts | C | S | - |

| | | | | | | | | |
|---|---|---|---|---|---|---|---|---|
| 276 | sf006073 | BX927363 | GI:112183280 | A/G | Ts | C | NS | 39 |
| 277 | sf006078 | BT059612 | GI:223649133 | A/T | Tv | NC | - | - |
| 278 | sf006081 | FJ969488 | GI:262189341 | G/T | Tv | NC | - | 33 |
| 279 | sf006102 | GU129139 | GI:290760560 | A/C | Tv | C | NS | - |
| 280 | sf006197 | NM_001140134 | GI:213511349 | A/T | Tv | NC | - | - |
| 281 | sf006410 | XM_002663717 | GI:292618742 | A/G | Ts | NC | - | 9 |

**Table S4 Sequences of the contig build from the assembly of the 454 raw data**

Table S4 is available for download at http://www.g3journal.org/lookup/suppl/doi:10.1534/g3.111.001867/-/DC1.

**Table S5 Description of the consenssus and sex-specific linkage maps build using Crimap in brook charr, *Salvelinus fontinalis.* SNP: single nucleotide polymorphism; Msats: microsatellites**

| Map | Consensus | | | Female | | | Male | | |
|---|---|---|---|---|---|---|---|---|---|
| Linkage group | Number of markers (SNP/Msats) | Total length (cM) | Average marker spacing (cM) | Number of markers (SNP/Msats) | Total length (cM) | Average marker spacing (cM) | Number of markers (SNP/Msats) | Total length (cM) | Average marker spacing (cM) |
| 1 | 16 (11/5) | 132.2 | 8.26 | 16 (11/5) | 187 | 11.6 | 16 (11/5) | 77.8 | 4.23 |
| 2 | 16 (11/5) | 130.2 | 8.14 | 16 (11/5) | 169.3 | 10.5 | 16 (11/5) | 65.4 | 4.08 |
| 3 | 15 (10/5) | 95.7 | 6.38 | 15 (10/5) | 101.1 | 6.74 | 15 (10/5) | 33.3 | 2.22 |
| 4 | 8 (6/2) | 73.2 | 9.15 | 8 (6/2) | 65.4 | 8.2 | 8 (6/2) | 24.1 | 3.01 |
| 5 | 11 (6/5) | 95.2 | 8.65 | 11 (6/5) | 70.3 | 6.39 | 11 (6/5) | 28.3 | 2.57 |
| 6 | 11 (9/2) | 77.5 | 7.04 | 11 (9/2) | 67.8 | 6.2 | 11 (9/2) | 25.9 | 2.35 |
| 7 | 9 (6/3) | 46.6 | 5.17 | 9 (6/3) | 34.5 | 3.8 | 9 (6/3) | 12.3 | 1.36 |
| 8 | 11 (8/3) | 81.2 | 7.38 | 10 (7/3) | 129.3 | 12.9 | 10 (7/3) | 44.2 | 4.42 |
| 9 | 10 (9/1) | 82.3 | 8.23 | 10 (9/1) | 45.4 | 4.5 | 10 (9/1) | 26.5 | 2.65 |
| 10 | 9 (8/1) | 63.6 | 7.06 | 9 (8/1) | 43.6 | 4.8 | 8 (7/1) | 17.8 | 2.22 |
| 11 | 8 (4/4) | 70.1 | 8.76 | 8 (4/4) | 64.4 | 8.1 | 8 (4/4) | 25.9 | 3.23 |
| 12 | 6 (4/2) | 78.4 | 13.06 | 6 (4/2) | 58.1 | 9.7 | 6 (4/2) | 24.8 | 4.13 |
| 13 | 7 (5/2) | 74 | 10.57 | 7 (5/2) | 67.9 | 9.7 | 7 (5/2) | 26.9 | 3.84 |
| 14 | 6 (4/2) | 68.5 | 11.41 | 6 (4/2) | 52.1 | 8.7 | 6 (4/2) | 21.3 | 3.55 |
| 15 | 6 (5/1) | 52.6 | 8.76 | 6 (5/1) | 42.6 | 7.1 | 6 (5/1) | 17.4 | 2.9 |
| 16 | 4 (1/3) | 42.8 | 10.7 | 4 (1/3) | 29.9 | 7.4 | 4 (1/3) | 11.9 | 2.97 |
| 17 | 6 (4/2) | 30.3 | 5.05 | 6 (4/2) | 27.5 | 4.6 | 6 (4/2) | 21.3 | 1.83 |
| 18 | 5 (2/3) | 24.9 | 4.98 | 5 (2/3) | 34.5 | 6.9 | 5 (2/3) | 6.9 | 1.38 |
| 19 | 4 (3/1) | 61.1 | 15.27 | 4 (3/1) | 66.8 | 16.7 | 4 (3/1) | 22.4 | 5.6 |
| 20 | 5 (3/2) | 67.2 | 13.44 | 5 (3/2) | 71.2 | 14.2 | 5 (3/2) | 21.9 | 4.38 |
| 21 | 6 (5/1) | 60.7 | 10.11 | 6 (5/1) | 61.2 | 10.2 | 6 (5/1) | 20.4 | 3.4 |

| | | | | | | | | | |
|---|---|---|---|---|---|---|---|---|---|
| 22 | 5 (2/3) | 45.8 | 9.16 | 5 (2/3) | 77.8 | 15.6 | 5 (2/3) | 27.1 | 5.42 |
| 23 | 4 (3/1) | 29.2 | 7.3 | 4 (3/1) | 33.9 | 8.5 | 4 (3/1) | 11.4 | 2.85 |
| 24 | 5 (4/1) | 49.3 | 9.86 | 5 (4/1) | 66.1 | 13.2 | 5 (4/1) | 20.3 | 4.06 |
| 25 | 4 (4/0) | 29.8 | 7.45 | 4 (4/0) | 35.6 | 8.9 | 4 (4/0) | 11.2 | 2.8 |
| 26 | 3 (2/1) | 11.5 | 3.83 | 3 (2/1) | 34.1 | 11.4 | 3 (2/1) | 4.5 | 1.5 |
| 27 | 3 (2/1) | 27.8 | 9.26 | 3 (2/1) | 43.5 | 14.5 | 3 (2/1) | 11.5 | 3.83 |
| 28 | 4 (4/0) | 3.9 | 0.975 | 4 (4/0) | 23.1 | 5.7 | 4 (4/0) | 2.8 | 0.7 |
| 29 | 2 (1/1) | 42.3 | 21.15 | 2 (1/1) | 44.4 | 22.2 | 2 (1/1) | 24.5 | 12.25 |
| 30 | 3 (2/1) | 63.9 | 21.3 | 3 (2/1) | 22.4 | 7.5 | 3 (2/1) | 32.3 | 10.76 |
| 31 | 17 (13/4) | 72.3 | 4.25 | 16 (12/4) | 121.2 | 7.5 | 15 (11/4) | 41.9 | 2.79 |
| 32 | 3 (2/1) | 59.1 | 19.7 | 3 (2/1) | 78.5 | 26.2 | 3 (2/1) | 32.5 | 10.83 |
| 33 | 2 (2/0) | 1.4 | 0.7 | 2 (2/0) | 3.6 | 1.8 | 2 (2/0) | 1.1 | 0.55 |
| 34 | 3 (3/0) | 41.8 | 13.93 | 3 (3/0) | 66.1 | 22.1 | 3 (3/0) | 24.5 | 8.16 |
| 35 | 3 (3/0) | 2.4 | 0.8 | 3 (3/0) | 11.5 | 3.8 | 3 (3/0) | 3.8 | 1.26 |
| 36 | 3 (2/1) | 29.1 | 9.7 | 2 (1/1) | 48.7 | 24.3 | 3 (2/1) | 20.3 | 6.76 |
| 37 | 3 (3/0) | 6.5 | 2.16 | 3 (3/0) | 12.7 | 4.2 | 3 (3/0) | 4.5 | 1.5 |
| 38 | 11 (5/6) | 12.9 | 1.17 | 11 (5/6) | 24.1 | 2.2 | 10 (4/6) | 5.9 | 0.59 |
| 39 | 5 (5/0) | 4.5 | 0.9 | 5 (5/0) | 5.2 | 1.1 | 5 (5/0) | 2.1 | 0.42 |
| 40 | 4 (4/0) | 5.6 | 1.4 | 4 (4/0) | 5.9 | 1.4 | 4 (4/0) | 2.4 | 0.6 |
| **Total** | **266** | **2047.4** | **8.31** | **263** | **2248.3** | **9.53** | **261** | **861.3** | **3.6** |
