# Supplementary material for: Coding Gene Single Nucleotide Polymorphism Mapping and Quantitative Trait Loci Detection for Physiological Reproductive Traits in Brook Charr, Salvelinus fontinalis
Source: G3 (Bethesda). 2012 Mar 1;2(3):379–92. doi: 10.1534/g3.111.001867 (PMC3291508; doi:10.1534/g3.111.001867)
Supplement: Supporting Information [file supp_2.3.379_TableS1.pdf]

Table S1 Description of the SSR markers used to build the linkage map in the Brook charr, *Salvelinus fontinalis*

	Marker	Primer F et R	Annealing Tempearature	Accession Number	Associated publication
1	BHMS206	CCAAATAACTGACAAGTGAG	54	AF256680	Timusk et al. 2011
		CAGAGGTTGATAATGGGG			
2	BHMS238	GATAATGCCTGGATGTGC	54	AF256682	Timusk et al. 2011
		CTAGAGCCGACCCTTTAC			
3	BHMS272	AGCTTGACAGCAGCTTGG	54	AF256690	Timusk et al. 2011
		TGCAATGCAGACTGACTG			
4	BHMS331	CAGCACCAGAACATAACC	54	AF256744	Timusk et al. 2011
		AGCCATCAACACTCCCTG			
5	BHMS377	TGGCTACAACAGGGATAC	54	AF256707	Timusk et al. 2011
		AGTCTCTTACATGGAGGC			
6	BHMS417/i/ii/iii/	ACATAGACCATGACGCTC	54	AF256752	Timusk et al. 2011
		TGACACGCTCTCTGATCC			
7	BHMS429	CCCCTGTCAAACGTCTTC	54	AF256719	Thorsen et al., 2005
		AGCACACTGGATTCAAGG			
8	BHMS465/i/ii	ACTCATCAACTGAGCCCC	54	AF256857	Timusk et al. 2011
		GTGATCTGTAGTTTTCCATC			
9	BHMS7.011	GGGACAGCTAATGGATCG	54	AF256834	Timusk et al. 2011
		GTTTTAGTAATCGGAGTGTG			
10	BX073647/i/ii	TATGGCTTTCCAAACCAAAG	54	BX073647	Timusk et al. 2011
		ACAGGGGGTTAACAGTGACA			
11	BX073974	TGTACGGAAAGATGGGCTCC	54	BX073974	Timusk et al. 2011
		CTCGAAGTCACCCAAACAGC			
12	BX076085	AAGACAGGAGATGAAGACACCG	54	BX076085	Timusk et al. 2011
		ATATGTCGTGGGAAACATGTAGG			
13	BX079862	TGTGAGAAGAACACGAGAGTTGG	54	BX079862	Timusk et al. 2011

		GAATGAGGTGTTAGAACGACTGC			
14	BX087664/i/ii	ATAGCCAGAGGGAAGCCTGC	54	BX087664	Timusk et al. 2011
		GCATCTCCAGCAGTCATTTGG			
15	BX299451	CCTGGTCTCCTTCACTTTCA	54	BX299451	
		CTGTGCTTACTGGGCAACTT			
16	BX305863	TACTGTACAGGATGGGTCTCTGC	54	BX305863	Timusk et al. 2011
		GGAGTACTACGTGTGAGGATTGG			
17	BX311224	CCGTGTGAAACCTGCATCC	54	BX311224	Timusk et al. 2011
		CCTTGGAATGCTTACCCTACC			
18	BX311884/i/ii	TGGACAACTTCAGCAAGGACC	54	BX311884	Timusk et al. 2011
		TGGTGACAGTTTCTGCTGAACC			
19	BX313739	CATGGAGTGTAAGGCAGGGC	54	BX313739	Timusk et al. 2011
		CGACAGATCAGCATCGCTGC			
20	BX318599	GATTTTCTGACCAGCACCTCC	54	BX318599	Timusk et al. 2011
		CCAACTGGACCCAGAACAGC			
21	BX319197	CGTCATCCATGTATGTTTCATGC	54	BX319197	Timusk et al. 2011
		GACAACTCACACATCCACATGG			
22	BX319411/i/ii	GCTGTGGCCCTCTGTCATACC	54	BX319411	Timusk et al. 2011
		GACATCAACGTGACACCAGGC			
23	BX861121/i/ii	CCTTCATCAAGGATGCAGCC	50	BX861121	Timusk et al. 2011
		TATGTTTCCCAGTACATACCGGG			
24	BX870052/i/ii	CAGATGAAATTGATCCAGATGACG	54	BX87005	Timusk et al. 2011
		GGAAGGGGATGATGTGATTGG			
25	BX873441	GAAGAGTTCCGGTCCATCGG	54	BX873441	Timusk et al. 2011
		CGTGCATGTAATTCAGCCTGC			
26	BX881655	AGAAAGACCTGGCAAGGACC	54	BX881655	Timusk et al. 2011
		CCTCTGGCACAAACTCCAGC			
27	BX890355/i/ii	ACTGAGAACACTTCAGCCAAGG	54	BX890355	Timusk et al. 2011

28	CA060381	ACCCACTAGCTGCTACATTATGG<br>AGTGGTGAAGTGGGATGGGG	54	CA060381	Rise et al., 2004
29	CA061336	CCCGATGCTTTCTTCATGG<br>TGCCATGTTTATTGAAATGCC	52	CA061336	Rise et al., 2004
30	CA344270	GGATCCAAAGGAGAGACTCCTG<br>CACACTTCCTGGACCACTTCC	54	CA344270	Rexroad et al., 2003
31	CA345149	TTCAAACTAGGGATTTCGTTGC<br>ACCACCTCAGAGTGTCTTTCTCC	54	CA345149	Rexroad et al., 2003
32	CA350064	GACTTGACATACAAACGACGTGG<br>GACCTGGTTTCGGTGTCAGAGC	54	CA350064	Rexroad et al., 2003
33	CA368462/i/ii	CCACTGTGAAGTTAGGTGTTCCC<br>CGACAGACTCAGACCACTGTCC	56	CA368462	Rexroad et al., 2003
34	CA376300/i/ii	CGACAGACTCAGACCACTGTCC<br>TTAAGCACTAAGGGGAAGACGG	54	CA376300	Rexroad et al., 2003
35	CA378164	TGGGGTGGAAGCAAAGAGC<br>GAACGGGGTGTCGTAGATGG	54	CA378164	Rexroad et al., 2003
36	CL4778	CCCTGACCTGTCCTCTTTGG<br>GAGGATACTGCCATTCAACA	54	NA	
37	Clock3-7C2-3	ACGGTCCCACCTTACCATAAA<br>GAGTACTGCCCTGCAGGTTG	54	NA	Timusk et al. 2011
38	CR363293	TTGACCATGGCCCTCTTATG<br>TCCGCAACAAGTACGCTGG	54	CR363293	Timusk et al. 2011
39	Ogo4UW	TTCTCTTCTGGCAACTTCAGACC<br>GTCGTCACTGGCATCAGCTA	54	AF009796	Olsen et al., 1998
40	Omi30TUF/i/ii	GAGTGGAGATGCAGCCAAAG<br>AGAAGACGAAGTGGATGCTG<br>GTCACCGTTTCTTTACCTGC	54	NA	Timusk et al. 2011

41	Omi126TUF	TTAAAGGAAACACACGCATACG	54	NA	Timusk et al. 2011
		TTCACACGACCGTTGGTG			
42	Omi179TUF	TTATCCTAGTGCCGGGTCTG	54	NA	Timusk et al. 2011
		ATGCAGCTTTTCAGTGGCTT			
43	OkeSLINRA	GAAAATAACTATAGACATTGCTGG	54	NA	Sakamoto et al., 2000
		CGTCCTTACACTCCAGAGGG			
44	OMM1195	GCGAGGTTAGGATACACACAT	54	AF469980	Timusk et al. 2011
		CTTCAGCCTGGAAACACAA			
45	OMM1197/i/ii	CTAGGAGAACAAGAAGACCATCGC	54	AF469982	Timusk et al. 2011
		AGGACAGAAAGGAGGTAAAACGG			
46	OMM1201	CCGGAAAAGCTAGGGAGAG	54	AF469986	
		CCCTTCTGTATCCATTCCGTT			
47	OMM1205	AAACGGTGCCCTCCTTCCTCTATA	54	AF469990	Timusk et al. 2011
		CCCAAGCCAATAAAGCCCTTACAT			
48	OMM1210	CATCAGACAGCACAGAGCAG	54	AF469994	Timusk et al. 2011
		GGAGGAGCAAGCCTTCTAAC			
49	OMM1211	ACCCACTCTCCCACTCAGTATT	54	AF469995	Timusk et al. 2011
		GAAGGAGGCTTGGAAGTGTATC			
50	OMM1220	CTCTGGGACAGACTTATCAC	54	AF470002	Timusk et al. 2011
		CTATTGGACGATGCACAC			
51	OMM1228	CCCTTCCTGTGTGTCGTTGTT	54	AF470009	Timusk et al. 2011
		CAGGAGTCACTTGGCAGTAGGAG			
52	OMM1237/i/ii	GTCAGAGTCGTGGGTATCAA	54	AF470017	Timusk et al. 2011
		CAGAGTTTCCACGGTCACT			
53	OMM1238	CGGAAATACGGAGGCTACTGTTG	54	AF470018	Timusk et al. 2011
		CTTCTCCCTGGCATCTTTCATCAG			
54	OMM1263/i/ii	CTGCATTCCAATACTCCACAG	54	AF470029	Timusk et al. 2011
		TGGACGAACACTGGATCAG			

55	OMM1290	GCCTCAGCACTGTCTTAA	54	AF470050	Timusk et al. 2011
		CGGAGGTCCCTAGAGA			
56	OMM1329	GGGAAGTGTTCACCATTACACAAG	54	G73564	Palti, Y et al., 2002
		CATCCAGGAACGCACCTTTA			
57	OMM1345	CCCTGGATTCTCCTGTTAG	54	G73576	Palti, Y et al., 2002
		ACATAGACACAGCACTCATGG			
58	OMM1372/i/ii	CACTTCATGATGCCGAAAGCAG	54	BV005159	Palti, Y et al., 2002
		CCCCCATCATGACTCCTTCTAGTT			
59	OMM1445	CTGCGTTATTGGTAGCTTGTG	54	BV079589	Timusk et al. 2011
		CCCGGTAATGTAGTTCCTGTC			
60	OMM1459	GCAGGTATTCAGGTAGGTCAG	54	BV079593	Timusk et al. 2011
		AATGACCATGGAAAACAACAC			
61	OMM1512	TTTCAAATCAGCCCAGGTTA	54	BV212048	Coulibaly et al., 2005
		AGGAAAAGGCAGGATGGTAT			
62	OMM1579	CTAGGCTCTGTGAATCTGA	54	BV212097	Coulibaly et al., 2005
		GAAGGAATAAGACTGTCCG			
63	OMM3015/i/ii	ACTCTTGCGCTGGTTGTATG	54	BV718488	Timusk et al. 2011
		GAAGAGTGTGAAAGTTTGGCTG			
64	OMM3075	CATTTAATTGAGCTGGCCAC	54	BV676508	Timusk et al. 2011
		CCAGACAGTTCTGAGCAACC			
65	OMM3095	CTTCCATTCAGGGTAGAGCAC	54	BV676517	Timusk et al. 2011
		CCAGGTGTGAAAGGGTTTG			
66	OMM5000/i/ii	AACAGAGCAGTGAGGGGACTGAGA	54	CO805106	Timusk et al. 2011
		CAAGTGATGTTGGTGCGAGGG			
67	OMM5007	AGATGCCTGTCGAGTGTTG	54	CO805113	Timusk et al. 2011
		GAGGAGCATCATTTAGAGACTACA			
68	OMM5008	CTGTTTCGTTGTCCTCATATCAACC	54	CO805114	Timusk et al. 2011
		TCCATTATCCAATCAGGAGAGCTCTAT			

69	OMM5014/i/ii	GGGTCTGAAAGGAGCATGG<br>GGAACCTAACATGACGCAACA	54	CO805119	Timusk et al. 2011
70	OMM5018	GAAGGAACGGAACAGAGTGGTAATCAC<br>TCGGACAGGTAACTGGAACGGAT	54	CO805123	Timusk et al. 2011
71	OMM5019	CATGCTGCCTCTCACCGTTTA<br>AACACACCCAGCATCCAACC	54	CO805124	Timusk et al. 2011
72	OMM5056	TCACCATCACCTTCATCGCCT<br>ACATGCTGCCCTTTGACGGAG	54	CA349207	Rexroad et al., 2003
73	OMM5060	TCTCGGGCCAAACCTTCTTATTGC<br>AGCCACTACATCTCCACGCCCTT	54	CA348688	Rexroad et al., 2003
74	OMM5061	GCGTTGGGAGAGAACAATACC<br>CCCATCACACCAGTTGCC	54	CA348688	Rexroad et al., 2003
75	OMM5074	TCGCTTTGGGTAGAAGTTGCCTTTAAC<br>AACATTAAGAACGAGTGGAATCACGC	54	CA348721	Rexroad et al., 2003
76	OMM5091	GCAGGAAAAACACCCAGATACAA<br>ACACTGGCTGGTGTCGTTACATTA	54	CA348850	Rexroad et al., 2003
77	OMM5102/i/ii	ATTCCAAATAACAGGTGCTACTGGTC<br>CTGGTTAACTAGGCAACTGATTGTGTC	54	CA348955	Rexroad et al., 2003
78	OMM5113	TCGGTAACAAGTCCTCTAGACCACA<br>CAGAGACCTAGACTGAGTCATGTCCTG	54	CA349018	Rexroad et al., 2003
79	OMM5146	GACAGATTCATGCAAGCCT<br>CCTCACTACTTGCCAATCA	54	BV211874	Coulibaly et al., 2005
80	OMM5147	CACTGTATGTTCTTACCCTG<br>TATACTGGCTGAGTTCAACC	54	BV211875	Coulibaly et al., 2005
81	OMM5155i/ii	GGACAGAACTGCCACTAAGTGTG<br>GAGGAGACAGGGAAGAGCTATTG	54	BV211883	Coulibaly et al., 2005
82	OMM5161/i/ii	CAAGTGTCCTTTGAGCAC<br>AGCAACTGCTGACACTCC	54	BV211889	Coulibaly et al., 2005

83	OMM5176	CCACTTGCTGCTTCTCTACATA	54	BV211902	Coulibaly et al., 2005
		AAGAACACCTAGCCAATAACCC			
84	OMM5179	CCCTGTCACATGGATGCT	54	BV211905	Coulibaly et al., 2005
		GATTTGGCAACCGAACAC			
85	OMM5312/i/ii/iii	ACTGTCAGCAGCAATACACT	54	BV21202	Coulibaly et al., 2005
		CCCATTTTCTCTTGTCACAC			
86	Omy6DIAS	CCACCAACTTCTTACATGAT	54	AF239042	Timusk et al. 2011
		CTATGGGGACAGCCGAATAA			
87	Omy21INRA/i/ii/iii	GCATTGGCGTAATGAGAAGG	54	NA	Gharbi et al., 2006
		CTGACGGACATATCAGCCC			
88	OmyRGT2TUF	ATAATGTGTCCCCAGGCAAG	54	AB087587	Sakamoto et al., 2000
		GAGGATGCGTCTTTGCATCT			
89	OmyRT16TUF	TGGCTGAGTTATACATGGAACG	54	NA	Timusk et al. 2011
		TCAATCAGGAGCAGTTAAAACA			
90	Otsclock1b_44_L_2	TGCTACTGTGGCAACCTTTG	54	NA	
		CCTGAGAGAGATGAGGGAGAGA			
91	Sal5UoG	TTTGCATTGAGCCTCTGTTG	54	NA	Timusk et al. 2011
		TGTTTCAGCTGCTATTAGGAAT			
92	Sal9UoG/i/ii/iii	TCACTGCTTCAAGGTATTTTTACTT	54	NA	Timusk et al. 2011
		AATTAGAGCTGCTAGGTCAGTGAG			
93	SalD25SFU	GATCTACACAGACCCCCACC	54	AF537305	McGowan et al., 2004
		CCGTTCTTCCAATAACTGCTC			
94	SalD39SFU	GGGGAGTCTGTGTTAAGTTGG	54	AF537310	McGowan et al., 2004
		TGAATGGACGTTCCTCTGAC			
95	SalE38SFU	CGCCTTGTCATACATTACACC	54	AF537309	McGowan et al., 2004
		CCGTTCTTCCAATAACTGCTC			
96	SalF41SFU	ATCCGCTATGAACCACAGG	54	AF537306	McGowan et al., 2004
		ACTGCTCCGGCAACTACAG			

97	Ssa0017BSFU	CGCAACAGGCTTAGTTAGAT	54	AF019154	Timusk et al. 2011
		CAACATTCAGTTCCCTTCAT			
98	Ssa0033BSFU	ATTCTTGATCGTGGTCTTTG	54	NA	Phillips et al., 2009
		CCGTTCTTCCAATAACTGCTC			
99	Ssa0072BSFU	TCCGAAGATAGGGGAGGTT	54	NA	Phillips et al., 2009
		ATAAACATCTGGGTGGCTGC			
100	Ssa0080BSFU/i/ii	CTACTGCACAGCACCTGGAA	54	NA	Phillips et al., 2009
		CACAGAGCACCTCCTGAACA			
101	TC126859i/ii	TTTTCTCCCTTTGACGACAG	54	NA	Phillips et al., 2009
		TGGTTGTGACTCGATGTCTG			
102	C113	GGAGCCCAGACTATATTGACG	64	NA	King TL, unpublished
		CCTTGAAGTCTTGCCAGAT			
103	B52	GCACACGAAACCAGTATATTTC	64	NA	King TL, unpublished
		TTGTCTTGGTGATTTCAGAGC			
104	C28	CAGTTGAAGTGATTGGGTTAGC	64	NA	King TL, unpublished
		TCATCCTTAAAGCAGAATACCAC			
105	C129	AGTGGGTACAACATACCTTTGG	64	NA	King TL, unpublished
		AGGTATTCACACCTCAGATTGG			
106	C88	TAGTCTCTGGTGGGGAATAATG	60	NA	King TL, unpublished
		ATATCAGCCATAAGAGCTGGAG			
107	C24	GCTACTGTTGGATTTCATCTCAG	60	NA	King TL, unpublished
		ATCACAGAGATGGGGTGATG			
108	D100	ACCTTTGACCTGTACATCGTG	60	NA	King TL, unpublished
		CAGACCTAGACTAAAGCATCCG			
109	D75	GTAGTGCCAAAACAGGTAGAGC	60	NA	King TL, unpublished
		CATCCTTATTCCAACCTCAATC			
110	C86	ACCGATGGCCTTCAACAC	60	NA	King TL, unpublished
		ATAGGCCCCTACCTCAAACC			

111	Sfo266	CTGGCAGCATTGTAAAGAAG	64	NA	Perry et al., 2005
		CTGGGTGATTTGACGACC			
112	Sco216	CCTTGTGAGAGCTAAGGTAGTG	64	NA	Dehaan and Ardren 2005
		GGAGGACATATTCCAACTTTG			
113	Sfo262	CCCATGTCAGTATTGGACTC	64	NA	Perry et al., 2005
		CTTCATGGGCAGAATGGAC			
114	Sco218	TTCTAACTGTTGGCACTCTG	60	NA	Dehaan and Ardren 2005
		GTGTGGTTGGGTGGTAAG			
115	SFO241	CTCCATTAGAAAGGGTTTG	60	NA	King TL, unpublished
		CCAGTCTTTAGTCAACGC			
116	SFO091	AAATAACAACAATATGTGAGAAC	60	NA	King TL, unpublished
		TATGCTGATATTGACTTTGG			
117	SFO308	CAGCAATGGGGCTGAAGTAG	60	NA	Perry et al., 2005
		GTCACTGTGTGAATCCTCC			
118	SFO269	GTAGATGAAACCTGATGG	60	NA	Perry et al., 2005
		GTTCTATGGTCACATACTG			
119	SFOD105	CAGGGAAAATGCTAATGTGC	60	NA	King TL, unpublished
		GGTTGTGTCGAATGGAGTTC			
120	Ssa85	AGCTGGGTCCTCCAAGCTAC	60	NA	King TL, unpublished
		ACCCGCTCCTCACTTAATC			
121	SFOC115	CAGTTTCTATCTCCAGGCAATC	60	NA	King TL, unpublished
		TTCTGAAAGCACTCAACATGG			
122	SSA197	GGGTTGAGTAGGGAGGCTTG	60	NA	O'Reilly et al., 1996
		TGGCAGGGATTTGACATAAC			
123	SFO12	GGTTTTGAAGAGTGACAG	60	NA	Angers et al., 1995
		CCCGTTTCACAATCAGAG			
124	SFO177	CGAATGTGGAGCTGAACTG	60	NA	Perry et al., 2005
		GGGTATTTGTACAATGGGT			

125	ONE8	AACATTCTGGGATGACAGGGGTA<br>CTGTTCTGCTCCAGTGAAGTGGA	60	NA	Scribner et al., 1996
126	SFO226	GAGGGCTAGAGACTAGCTTCAG<br>GCAGTGGAACAAATACCCAG	60	NA	Perry et al., 2005

McGowan, CR, Davidson, EA, Woram, RA, Danzmann, RG, Ferguson, MM and Davidson, WS, 2004. Ten polymorphic microsatellite markers from Arctic charr (Salvelinus alpinus): linkage analysis and amplification in other salmonids Anim. Genet. 35:479-481.

Olsen, JB, Bentzen, P, and Seeb, JE, 1998. Characterization of seven microsatellite loci derived from pink salmon. Mol. Ecol. 7: 1087-1089

O'Reilly P, Hamilton L, McConnell S, Wright J (1996). Rapid analysis of genetic variation in Atlantic salmon (Salmo salar) by PCR multiplexing of dinucleotide and tetranucleotide microsatellites. Canadian Journal of Fisheries and Aquatic Sciences 53: 2292-2298.

Phillips, RB, Keatley, KA, Morasch, MR, Ventura, AB Lubieniecki, KP, Koop, BF, Danzmann, RG and Davidson, WS, 2009. Assignment of Atlantic salmon (Salmo salar) linkage groups to specific chromosomes: Conservation of large syntenic blocks corresponding to whole chromosome arms in rainbow trout (Oncorhynchus mykiss) BMC Genetics 10:46

Palti, Y, Fincham, MR and Rexroad, CE, III, 2002. Characterization of 38 polymorphic microsatellite markers for rainbow trout (Oncorhynchus mykiss). Mol. Ecol. Notes. 2: 449-452

Perry GML, King TL, St-Cyr J, Valcourt M, Bernatchez L 2005. Isolation and cross-familial amplification of 41 microsatellites for the brook charr (Salvelinus fontinalis). Molecular Ecology Notes 5(2): 346-351.

Rexroad, CE, III, Lee, Y, Keele, JW, Karamycheva, S, Brown, G, Koop, B, Gahr, SA, Palti, Y, and Quackenbush, J. 2003. Sequence analysis of a rainbow trout cDNA library and creation of a gene index. Cytogenet. Genome Res. 102: 347-354

Rise, ML, von Schalburg, KR, Brown, GD, Mawer, MA, Devlin, RH, Kuipers, N, Busby, M, Beetz-Sargent, M, Alberto, R, Gibbs, AR, Hunt, P, Shukin, R, Zeznik, JA, Nelson, C, Jones, SR, Smailus, DE, Jones, SJ, Schein, JE, Marra, MA, Butterfield, YS, Stott, JM, Ng, SH, Davidson, WS and Koop, BF, 2004. Development and application of a salmonoid EST database and cDNA microarray: data mining and interspecific hybridization characteristics. Genome Res 14:478-490

Sakamoto,T, Danzmann, RG, Gharbi, K, Howard, P, Ozaki, A, Sokkean, K, Woram, RA, Okamoto, N, Ferguson, MM, Holm, L-E, Guyomard, R and Hoyheim, B, 2000. A microsatellite linkage map of rainbow trout (*Oncorhynchus mykiss*) characterized by large sex-specific differences in recombination rates. Genetics 155:1331-1345.

Scribner KT, Gust JR, Fields RL (1996). Isolation and characterization of novel salmon microsatellite loci: cross-species amplification and population genetic applications. Can J Fish Aquat Sci 53: 833-841.

Timusk, E. R., M. Ferguson, H. K. Mogahadam, J. D. Norman, C. Wilson *et al.*, 2011. Genome evolution in the fish family salmonidae: generation of a brook charr genetic map and comparisons among charrs (Arctic charr and brook charr) with rainbow trout. BMC genetics 68:12.

Thorsen, J, Zhu, B, Frengen, E, Osoegawa, K, de Jong, PJ, Koop, BF, Davidson, WS and Hoyheim, B, 2005. A highly redundant BAC library of Atlantic salmon (*Salmo salar*): an important tool for salmon projects. BMC Genomics 6:50
