# Supplementary material for: Coding Gene Single Nucleotide Polymorphism Mapping and Quantitative Trait Loci Detection for Physiological Reproductive Traits in Brook Charr, Salvelinus fontinalis
Source: G3 (Bethesda). 2012 Mar 1;2(3):379–92. doi: 10.1534/g3.111.001867 (PMC3291508; doi:10.1534/g3.111.001867)
Supplement: Supporting Information [file supp_2.3.379_TableS2.pdf]

Table S2 Description of the Sequenom panel used to genotype the SNP markers in the F2 progeny

Technology	Panel	Marker Name	Oligo_Forward	Forward_oligo_Sequence	Oligo_Reverse	Reverse_Oligo_Sequence	Oligo_Probe	Probe_Sequence
sequenom	SEQ010	sf000032_01CT	sf000032_01CT_GA_F	ACGTTGGATGGGCGTCCCTGTTGAATTATC	sf000032_01CT_GA_R	ACGTTGGATGGGCACACCCTTCTTTATCAC	sf000032_01CT_GA_Pa	CTTTATCACACACACTATTACTG
sequenom	SEQ010	sf000124_02CT	sf000124_02CT_AG_F	ACGTTGGATGGCCCAGTTGCCTATAGATTC	sf000124_02CT_AG_R	ACGTTGGATGGTCTCCCTTATTCCCTTTGC	sf000124_02CT_AG02_P	CCTTATTCCCTTTGCACCAGTTTGTTAG
sequenom	SEQ010	sf000178_01CT	sf000178_01CT_GA_F	ACGTTGGATGCTTGCCACTTTTCCTGGTAG	sf000178_01CT_GA_R	ACGTTGGATGCTTGTCTGGGACAAACTGAG	sf000178_01CT_GA_Pa	GGGACAAACTGAGATACGA
sequenom	SEQ010	sf000191_01AG	sf000191_01AG_GC_F	ACGTTGGATGGACCTCTGTTTTACCGTGAG	sf000191_01AG_GC_R	ACGTTGGATGGGCACTTCTTTGTATTGTGG	sf000191_01AG_GC02_Pa	CCACTTGATAGGCAGCCA
sequenom	SEQ010	sf000228_01AC	sf000228_01AC_TA_F	ACGTTGGATGTACAACAATTGTTCCCACCC	sf000228_01AC_TA_R	ACGTTGGATGGTTCCCACAATCAACCACTG	sf000228_01AC_TA_Pa	TGCTCCAATCACAGGT
sequenom	SEQ010	sf000327_02AC	sf000327_02AC_GA_F	ACGTTGGATGATGAGTGACAGACGAGCTAC	sf000327_02AC_GA_R	ACGTTGGATGTGCCTTGGTAGTCTATTCTC	sf000327_02AC_GA_Pa	AAGACAAAGACTAACAAGTTAATTAC
sequenom	SEQ010	sf000657_01CT	sf000657_01CT_TC_F	ACGTTGGATGTTTCACACACTGTTCGAGGG	sf000657_01CT_TC_R	ACGTTGGATGCGGTTTTGAACCGATGAAGC	sf000657_01CT_TC_Pa	AAAAAATGTTCGAGGGTTTCTATG
sequenom	SEQ010	sf000679_01CT	sf000679_01CT_TC_F	ACGTTGGATGGGATATTTTAGTGACATCGG	sf000679_01CT_TC_R	ACGTTGGATGCCAGTGTGACAAAGGTTATG	sf000679_01CT_TC_Pa	CATTGTAATATGTGTATGTAGGCCTAGTA
sequenom	SEQ010	sf000810_03CT	sf000810_03CT_CT_F	ACGTTGGATGCCTAAACACAGAAACAAGGG	sf000810_03CT_CT_R	ACGTTGGATGTGAAGTATGCAAGGAGGGAG	sf000810_03CT_CT02_P	GGCAAGGAGGGAGTTTATCACAGA
sequenom	SEQ010	sf000878_01AG	sf000878_01AG_CT_F	ACGTTGGATGACGTTAGTTGGGTGCAAGGG	sf000878_01AG_CT_R	ACGTTGGATGAACAAACTAGGCTAGCTCCC	sf000878_01AG_CT02_P	CCCCTAGTTAACTCTGGAAAATAGGA
sequenom	SEQ010	sf000891_02AC	sf000891_02AC_CA_F	ACGTTGGATGCAGAAATCCTACACTCGTCC	sf000891_02AC_CA_R	ACGTTGGATGTCCTTCTTTAAAGAGGCAGG	sf000891_02AC_CA02_Pa	GGAGAGACGGAAGATTC
sequenom	SEQ010	sf000961_01AC	sf000961_01AC_CT_F	ACGTTGGATGCCCCTCAAGGATAGAAGAAC	sf000961_01AC_CT_R	ACGTTGGATGACTGTAATACAGAAACCAGC	sf000961_01AC_CT_P	ACCCAGCTATTCTCTGAATGTACAATT
sequenom	SEQ010	sf000970_01AC	sf000970_01AC_GA_F	ACGTTGGATGAGGAAGCGATGAGTTAGGAC	sf000970_01AC_GA_R	ACGTTGGATGAAATGGCAACTCGGTTGAGG	sf000970_01AC_GA_Pa	TAAACTCGGTTGAGGGAGGGAGATA
sequenom	SEQ010	sf001181_01CT	sf001181_01CT_GA_F	ACGTTGGATGTGTGCAGTGATCCACAGTTC	sf001181_01CT_GA_R	ACGTTGGATGCAAACACACACACAAACCCC	sf001181_01CT_GA_Pa	GTTCTGTTGGAGCATTG
sequenom	SEQ010	sf001661_01AC	sf001661_01AC_GT_F	ACGTTGGATGTCAGAATCCAGGGCTTGAAC	sf001661_01AC_GT_R	ACGTTGGATGGGGTACTTCAGAGCCAAATG	sf001661_01AC_GT02_P	TGTGTTGGGTTCAGAGAAT
sequenom	SEQ010	sf001740_01GT	sf001740_01GT_GA_F	ACGTTGGATGCCAAAGCCCAACAAAAAAGG	sf001740_01GT_GA_R	ACGTTGGATGGAGTTTCCTGAAAGTTCAC	sf001740_01GT_GA_Pa	TGAAAGTTCACATTAGACTCTAG
sequenom	SEQ010	sf002175_02GT	sf002175_02GT_TC_F	ACGTTGGATGTGCCTGACATCTGAACTCTG	sf002175_02GT_TC_R	ACGTTGGATGTTTTCACAGTGGCGGAACGG	sf002175_02GT_TC02_Pa	AGCGGAACGGGCCAATG
sequenom	SEQ010	sf003063_01AC	sf003063_01AC_CA_F	ACGTTGGATGACCCTCAGCCTATCATATCC	sf003063_01AC_CA_R	ACGTTGGATGGCAGTACCTACATTGTGTCC	sf003063_01AC_CA_P	GGTAGCAAATGTTGCTACAGCAACAATGAG
sequenom	SEQ010	sf003084_01AG	sf003084_01AG_AG_F	ACGTTGGATGCCATGGCTCTCTCTATAGAC	sf003084_01AG_AG_R	ACGTTGGATGGCAATATGGGGTAACACAAG	sf003084_01AG_AG_P	CAGCATTATTACTGGTACAATTAATGCATA
sequenom	SEQ010	sf003407_01AG	sf003407_01AG_TC_F	ACGTTGGATGTGCAGGCCATTCCAAAAAGC	sf003407_01AG_TC_R	ACGTTGGATGCTGCCCATTCTGAAGAAACG	sf003407_01AG_TC02_Pa	CCTGAGCATGTTATTACCTGACAAGAA
sequenom	SEQ010	sf003601_01GT	sf003601_01GT_CT_F	ACGTTGGATGGTAAAGTAGCTGGTGCCTTG	sf003601_01GT_CT_R	ACGTTGGATGAGCCCCGGATTTATGTATGC	sf003601_01GT_CT_P	ACAGTAGCAACAAACCCCT
sequenom	SEQ010	sf003621_01AC	sf003621_01AC_TC_F	ACGTTGGATGGAGCCATAGGTAATGTCCAG	sf003621_01AC_TC_R	ACGTTGGATGCATTCCTACCGGAGTGTAAC	sf003621_01AC_TC02_Pa	CCAACTCTGTTTGAGATGTC
sequenom	SEQ010	sf003698_01AT	sf003698_01AT_GT_F	ACGTTGGATGTTGGTTGAAGGGAAAGGCTG	sf003698_01AT_GT_R	ACGTTGGATGAACACAGTGCCTTCTCACTC	sf003698_01AT_GT_Pa	TCTCACTCACCCTAAAACAAAT
sequenom	SEQ010	sf003713_01AG	sf003713_01AG_TA_F	ACGTTGGATGCGATTCAGACACATCTATGG	sf003713_01AG_TA_R	ACGTTGGATGTAGATAGCAGATCTCGGAAG	sf003713_01AG_TA_Pa	CTACTCCACATGTGATGTTAAT
sequenom	SEQ010	sf003724_01CG	sf003724_01CG_TC_F	ACGTTGGATGCTGGCTTTGCTTGTAAACCC	sf003724_01CG_TC_R	ACGTTGGATGGGAAAAATCTGCATGGTTTAC	sf003724_01CG_TC_Pa	CCCCCCATCTGCATGGTTTACCCATTG

sequenom	SEQ010	sf003897_01CT	sf003897_01CT_GA_F	ACGTTGGATGGGGAATCATAGGCAATGAGG	sf003897_01CT_GA_R	ACGTTGGATGGGCGTAGTCGGTACTTTATG	sf003897_01CT_GA02_Pa	CCATACCTCCAGCTTGACT
sequenom	SEQ010	sf003919_01CT	sf003919_01CT_CT_F	ACGTTGGATGCAGTCCCTTGGTATAATGGC	sf003919_01CT_CT_R	ACGTTGGATGGAATCCTGACCAGAGTAGTG	sf003919_01CT_CT_P	ACTGACCAGAGTAGTGACCCAATT
sequenom	SEQ010	sf004030_01AG	sf004030_01AG_AT_F	ACGTTGGATGGAGGAAAGACAGTGGGAAAG	sf004030_01AG_AT_R	ACGTTGGATGGGCTGGTCTAGCCTATTTAG	sf004030_01AG_AT_P	TGGATTCAAACATGTGAGC
sequenom	SEQ010	sf004070_25AC	sf004070_25AC_GA_F	ACGTTGGATGCACAAATTTCTGGGTCTGGG	sf004070_25AC_GA_R	ACGTTGGATGAGCTGTTAGCAGAAAGCTGG	sf004070_25AC_GA_Pa	ATCAATCAATCACACTCACTT
sequenom	SEQ011	sf000071_02CT	sf000071_02CT_CA_F	ACGTTGGATGCAGATTAGGCCTAGACAACG	sf000071_02CT_CA_R	ACGTTGGATGCACAGGTACTAAGAGTGTGC	sf000071_02CT_CA_Pa	GGACTAAGAGTGTGCTGAAATT
sequenom	SEQ011	sf000078_02AG	sf000078_02AG_AT_F	ACGTTGGATGAGGGAAAGGGTTTTAAGCGG	sf000078_02AG_AT_R	ACGTTGGATGGCCACATTAACATACACCTG	sf000078_02AG_AT_P	GACCTACAGAAGAAAATAAAATAAGC
sequenom	SEQ011	sf000137_01AG	sf000137_01AG_TA_F	ACGTTGGATGTTCGAAACGCAATCACCGAG	sf000137_01AG_TA_R	ACGTTGGATGACTGAATTGACGGGATCAGC	sf000137_01AG_TA_Pa	AGACATGGAGTCCTATACTAT
sequenom	SEQ011	sf000324_05AC	sf000324_05AC_CA_F	ACGTTGGATGTCTGGAAACTGCGTTCTACC	sf000324_05AC_CA_R	ACGTTGGATGAGACAGCGGATTTGAGACAG	sf000324_05AC_CA_P	GTCTCCACACTTTACTTCCT
sequenom	SEQ011	sf000390_01AC	sf000390_01AC_TC_F	ACGTTGGATGCACAGGTATACTGTTGGTCG	sf000390_01AC_TC_R	ACGTTGGATGACCATGGTACTCATGACTGG	sf000390_01AC_TC_Pa	TGGAGGGCTTTGACAT
sequenom	SEQ011	sf000397_07CG	sf000397_07CG_CG_F	ACGTTGGATGTTGCCTGTTTGAGTGTTTGG	sf000397_07CG_CG_R	ACGTTGGATGCAAAGACTAAACAACAGCCC	sf000397_07CG_CG_P	GTTGAGCACCAACCACAA
sequenom	SEQ011	sf000428_01AT	sf000428_01AT_AT_F	ACGTTGGATGACCGTATGTTATGCAGCCAG	sf000428_01AT_AT_R	ACGTTGGATGCTGGACAATCTAAGCACTGG	sf000428_01AT_AT02_P	ACATTAAGCATGGTAGGCTATATAA
sequenom	SEQ011	sf000584_01GT	sf000584_01GT_GA_F	ACGTTGGATGGGGACTCCACACATTACAAA	sf000584_01GT_GA_R	ACGTTGGATGAACTAACAGTTAGCATGGTG	sf000584_01GT_GA_Pa	GAGAAAAGCTGAACAAAGTAATTTTCTA
sequenom	SEQ011	sf000854_01CT	sf000854_01CT_GT_F	ACGTTGGATGCGGTGTCTGCGATGATTTAG	sf000854_01CT_GT_R	ACGTTGGATGTCTCCCTCCTCTCAATCTTC	sf000854_01CT_GT02_P	CACGTCGGCTCAGCATCA
sequenom	SEQ011	sf001086_01CT	sf001086_01CT_TC_F	ACGTTGGATGTACTGTCACGACCAACACTC	sf001086_01CT_TC_R	ACGTTGGATGATTTACACCCCAGGTCTAAC	sf001086_01CT_TC_Pa	TAACCCAGGTCTAACATACCT
sequenom	SEQ011	sf001184_01CT	sf001184_01CT_TC_F	ACGTTGGATGTCTCTCAACAGCAAGAAGGG	sf001184_01CT_TC_R	ACGTTGGATGCACTGCTCAGACGCTCTTTG	sf001184_01CT_TC02_Pa	CCAACACATTACTTGCTCATCAATATT
sequenom	SEQ011	sf001284_01AG	sf001284_01AG_GT_F	ACGTTGGATGGCTATTCCCCAAGTTTCCTC	sf001284_01AG_GT_R	ACGTTGGATGCGGCCGTTCTTTAAATTACC	sf001284_01AG_GT_Pa	CTTTTCCTCAAAGCCACA
sequenom	SEQ011	sf001770_01AG	sf001770_01AG_AG_F	ACGTTGGATGAGGCACTGGTGTCTTTGTTC	sf001770_01AG_AG_R	ACGTTGGATGTCACGCTTAACTAAGGTCGG	sf001770_01AG_AG_P	TAAGGTCGGCCCCTAT
sequenom	SEQ011	sf002024_02AC	sf002024_02AC_AG_F	ACGTTGGATGGCTATCTCTGGATAGAGAGG	sf002024_02AC_AG_R	ACGTTGGATGCCTGTGTGTTTGTACATGCG	sf002024_02AC_AG_P	TTGGCTTCCAGTTATACTACCAC
sequenom	SEQ011	sf002033_01AG	sf002033_01AG_TA_F	ACGTTGGATGAGTTGAGGTTGGAGGAGTAG	sf002033_01AG_TA_R	ACGTTGGATGGAAACTACACCTACTGTCAC	sf002033_01AG_TA_Pa	GGAGTAGTGATTGTCATTGG
sequenom	SEQ011	sf002155_01CT	sf002155_01CT_AG_F	ACGTTGGATGGTACCACCTTGATTGTCACG	sf002155_01CT_AG_R	ACGTTGGATGATGAAGCCTGATATTCCAAC	sf002155_01CT_AG02_P	CTCTTTTTAATCAAACATTCCATTTGCA
sequenom	SEQ011	sf002948_01CG	sf002948_01CG_GT_F	ACGTTGGATGAAGGTCAGAGCAGTGTTTCC	sf002948_01CG_GT_R	ACGTTGGATGATCTGAGCGGTAGATCTAGG	sf002948_01CG_GT02_P	CGACTCAGAAACGTTGGT
sequenom	SEQ011	sf003141_03AC	sf003141_03AC_TA_F	ACGTTGGATGTGTTGGATGCCTTTGTTCGG	sf003141_03AC_TA_R	ACGTTGGATGCTGACAGACTCCACTTTTCC	sf003141_03AC_TA02_Pa	AGTCCACTTTTCCCAGCGCAT
sequenom	SEQ011	sf003197_01AG	sf003197_01AG_GA_F	ACGTTGGATGCAGAGCACCTATGATGTGAC	sf003197_01AG_GA_R	ACGTTGGATGAGCCTCCTTTACGAGTTACC	sf003197_01AG_GA02_Pa	GGGGTACCTCCCCATTGTCCGTCTGATTC
sequenom	SEQ011	sf003589_01AC	sf003589_01AC_CT_F	ACGTTGGATGGCAGGGACACGAGTTATTTG	sf003589_01AC_CT_R	ACGTTGGATGTTCACACTCACCACGTATGC	sf003589_01AC_CT_P	CCTAGTCACTTTAACCCTA
sequenom	SEQ011	sf003610_01AT	sf003610_01AT_AT_F	ACGTTGGATGTAGCTGGGCGTGCTTAACTG	sf003610_01AT_AT_R	ACGTTGGATGGACTCTGAGAACGTATACCG	sf003610_01AT_AT_P	GGGTATACCGTAAACCCATTCCCCCC
sequenom	SEQ011	sf003700_02GT	sf003700_02GT_TC_F	ACGTTGGATGGCGGTGACTATACTGTTGTG	sf003700_02GT_TC_R	ACGTTGGATGTACAATCGACCGAGAGCATC	sf003700_02GT_TC_Pa	AGACCGAGAGCATCTCATTTATAC
sequenom	SEQ011	sf003728_02AT	sf003728_02AT_TC_F	ACGTTGGATGAACCAGCCTATCAAGTGGTC	sf003728_02AT_TC_R	ACGTTGGATGACAACAGGAACAGCATCCAG	sf003728_02AT_TC02_Pa	ACAGCATCCAGACTGAACATAG
sequenom	SEQ011	sf003836_01CT	sf003836_01CT_TA_F	ACGTTGGATGGAGGTTTCTACTGTCACCTG	sf003836_01CT_TA_R	ACGTTGGATGCGACTGCAACTGAAAGCAAC	sf003836_01CT_TA_Pa	AAACTGAAAGCAACATGGGCATGC

sequenom	SEQ011	sf004064_01AT	sf004064_01AT_CA_F	ACGTTGGATGGAACACAGTGGTCTCCATAG	sf004064_01AT_CA_R	ACGTTGGATGCACATGCCACCCAATATGAG	sf004064_01AT_CA02_Pa	AGTATAATAAACTAGGACTAATAGACTA
sequenom	SEQ011	sf004112_01AC	sf004112_01AC_AG_F	ACGTTGGATGATATGGCCTGTTGAAGTGGG	sf004112_01AC_AG_R	ACGTTGGATGCAGACAGGATTAGGAACGAC	sf004112_01AC_AG_P	ACTCATCTAAACGCTCC
sequenom	SEQ011	sf004602_04AG	sf004602_04AG_GA_F	ACGTTGGATGAAAGAGACAGTTCTGCCTCC	sf004602_04AG_GA_R	ACGTTGGATGCTAGGAATGCTTACTGTGAC	sf004602_04AG_GA02_Pa	TGGCCTCCATTACCTCAAAG
sequenom	SEQ011	sf006081_01AT	sf006081_01AT_GT_F	ACGTTGGATGCGTGGCGGATAGAATTACTC	sf006081_01AT_GT_R	ACGTTGGATGGTCAGGGCATAGCACTTTTC	sf006081_01AT_GT_P	TTCCCGCCTTACATCAAAGTTACCTGAA
sequenom	SEQ012	sf000023_02AC	sf000023_02AC_TC_F	ACGTTGGATGCAAACGTGATATCTGTCCCC	sf000023_02AC_TC_R	ACGTTGGATGAGTCCCCAGTCAAAGTGAAG	sf000023_02AC_TC_Pa	TTCCAATAATGGTCAGAATCA
sequenom	SEQ012	sf000179_01AC	sf000179_01AC_TC_F	ACGTTGGATGACAGCGGAGGAGATTTGTTC	sf000179_01AC_TC_R	ACGTTGGATGACCTGCATTGACACAGATGG	sf000179_01AC_TC_Pa	GGGAGCTATAGTGACAGCAGTTAG
sequenom	SEQ012	sf000209_01AG	sf000209_01AG_CT_F	ACGTTGGATGTCTGTTCATTAGCCAGAGCC	sf000209_01AG_CT_R	ACGTTGGATGCGGTCCGATCAGTTTTTCTC	sf000209_01AG_CT_P	AGCCATTGTGACTTAAATGTCTC
sequenom	SEQ012	sf000230_01CG	sf000230_01CG_TA_F	ACGTTGGATGTGAAAGTAGCCTGGGTTGTG	sf000230_01CG_TA_R	ACGTTGGATGCATGAGCGTCACCAACCAAT	sf000230_01CG_TA02_Pa	TGCGTCACCAACCAATGAAAAG
sequenom	SEQ012	sf000633_01AT	sf000633_01AT_GT_F	ACGTTGGATGGAGATTTCCACTCCTTCACC	sf000633_01AT_GT_R	ACGTTGGATGGCCGTTGATAGAACGGTAAC	sf000633_01AT_GT02_Pa	CAGGTCGTGGTTTTTAAATTGTCTGG
sequenom	SEQ012	sf000706_01CT	sf000706_01CT_AG_F	ACGTTGGATGGGTCAATGAATGCATCCTCG	sf000706_01CT_AG_R	ACGTTGGATGTTGCTATGGGCTGGACAATG	sf000706_01CT_AG_P	GCTGGACAATGGCAGAGGTGTCCAATTGCA
sequenom	SEQ012	sf000719_01CT	sf000719_01CT_AT_F	ACGTTGGATGTCAGCCTTCAGCTAAAACCC	sf000719_01CT_AT_R	ACGTTGGATGAATGGGAATGTCACTCGAGG	sf000719_01CT_AT_P	ATGAATGTGAATGTCACTCGAGGGGAGAG
sequenom	SEQ012	sf000905_02CT	sf000905_02CT_AT_F	ACGTTGGATGCTTGTTATGGGCGTCTCTTC	sf000905_02CT_AT_R	ACGTTGGATGCTGCAATTGGTCAAGTTGGG	sf000905_02CT_AT_P	AAACAATACACTTTCGAGTTTGTACA
sequenom	SEQ012	sf000966_03CT	sf000966_03CT_AG_F	ACGTTGGATGTCTCCCTGATGTGAGGAAAG	sf000966_03CT_AG_R	ACGTTGGATGCTGCAGCAGAACACACATAC	sf000966_03CT_AG_P	CCCCTCTGGTTGGCTCAGTCTT
sequenom	SEQ012	sf001069_01AG	sf001069_01AG_GT_F	ACGTTGGATGCCTGGTTTTCTCCTCAGATG	sf001069_01AG_GT_R	ACGTTGGATGGGGACAAACCTGCATGTTTC	sf001069_01AG_GT02_P	GTTCTTCTCATGGGCTTT
sequenom	SEQ012	sf001437_01AT	sf001437_01AT_AG_F	ACGTTGGATGTGTCGACCAGGGTGAGGTTT	sf001437_01AT_AG_R	ACGTTGGATGTTTCTCTTGTTCCAGGCGAG	sf001437_01AT_AG_P	GGGAAGGGTGAGGTTTGTCACAACC
sequenom	SEQ012	sf001601_01AC	sf001601_01AC_CT_F	ACGTTGGATGTCCAAGACCATGGATGAGAG	sf001601_01AC_CT_R	ACGTTGGATGATTGCAGGGATGTGCAGTAG	sf001601_01AC_CT_P	ATGTCTGACCAATACTGTCCTAT
sequenom	SEQ012	sf001644_01AG	sf001644_01AG_GT_F	ACGTTGGATGCAAACAGACATGTCATCACG	sf001644_01AG_GT_R	ACGTTGGATGTTACATGCACCAGATGCTCC	sf001644_01AG_GT_Pa	TTTTGGGGACCGAGCAAAGAATTTC
sequenom	SEQ012	sf001686_01CT	sf001686_01CT_GA_F	ACGTTGGATGGAGTTGAGCCGTTCTGTATC	sf001686_01CT_GA_R	ACGTTGGATGTCTGTCAGACAGTCCAGGTG	sf001686_01CT_GA_Pa	GGCAACGTGGTGACAG
sequenom	SEQ012	sf001996_02CG	sf001996_02CG_AG_F	ACGTTGGATGACCATGGGCCAAAAAGCTTG	sf001996_02CG_AG_R	ACGTTGGATGTTCATTCTCCACAGTGCCTC	sf001996_02CG_AG_P	TGCCTCACAAGTGCTA
sequenom	SEQ012	sf002203_01CT	sf002203_01CT_AG_F	ACGTTGGATGGGTTCAATCCCTTTGCATCC	sf002203_01CT_AG_R	ACGTTGGATGGGTGGCAGAACACCAGTTTT	sf002203_01CT_AG_P	CCTTTCAATGCTTTTCAGC
sequenom	SEQ012	sf002250_01AG	sf002250_01AG_CA_F	ACGTTGGATGAAAGATGGCTGATCTCCCTC	sf002250_01AG_CA_R	ACGTTGGATGTTGTGCACTTTTCGTCCTTG	sf002250_01AG_CA_P	CCCCACTACAAATGGGCCAAAACAAT
sequenom	SEQ012	sf002353_02AT	sf002353_02AT_CT_F	ACGTTGGATGATTTCAACCAGCCATGGGAG	sf002353_02AT_CT_R	ACGTTGGATGAGTTGTTGCCACTCACCCTG	sf002353_02AT_CT_P	GGTGGTTGATGCCCTT
sequenom	SEQ012	sf002792_01AG	sf002792_01AG_AG_F	ACGTTGGATGGTTCATTGTGGTGAACGGAC	sf002792_01AG_AG_R	ACGTTGGATGAAGAGTTTCAGTAGCCCTGC	sf002792_01AG_AG02_P	GGCAAGCCTCTGTAGAACAATGG
sequenom	SEQ012	sf003076_01CT	sf003076_01CT_AG_F	ACGTTGGATGCAGTGGTTTAGATGGTACGC	sf003076_01CT_AG_R	ACGTTGGATGACACCGCTATTTCCTGGTTG	sf003076_01CT_AG_P	CCCTTTTGGTCACATAAACTGAAATTAGGC
sequenom	SEQ012	sf003092_01CT	sf003092_01CT_TC_F	ACGTTGGATGTTCCAAGCCCTAGATTTGCC	sf003092_01CT_TC_R	ACGTTGGATGATATCAACTGACTGGAGGGC	sf003092_01CT_TC_Pa	ATTCATAATTCTATATCATTACAGC
sequenom	SEQ012	sf003118_01GT	sf003118_01GT_GA_F	ACGTTGGATGCTATGAGGAGTGGTGTGTTC	sf003118_01GT_GA_R	ACGTTGGATGCCATACAAAACCCTCTGTAG	sf003118_01GT_GA_Pa	GGAAAAAACCCTCTGTAGTGTATGGCGG
sequenom	SEQ012	sf003334_01CT	sf003334_01CT_AG_F	ACGTTGGATGTTATAGCTTCAAATAGGCCC	sf003334_01CT_AG_R	ACGTTGGATGGGCCTATGTAACCTAGCCAG	sf003334_01CT_AG_P	CCATTTATTTGTGTTCTGGAAAATGTGAAC
sequenom	SEQ012	sf003500_02CT	sf003500_02CT_CT_F	ACGTTGGATGAGAGGCGCGATGAATTTAAG	sf003500_02CT_CT_R	ACGTTGGATGTGACAGCTGACAATGTGCTC	sf003500_02CT_CT02_P	CGTGCTCTTGGCTCGAGC

sequenom	SEQ012	sf003595_01CT	sf003595_01CT_AT_F	ACGTTGGATGTGTACACTTTTCCAATGC	sf003595_01CT_AT_R	ACGTTGGATGGCCTAGTCACAGCATTTACG	sf003595_01CT_AT_P	CACAATAAAGTGTGTACAG
sequenom	SEQ012	sf003614_01CT	sf003614_01CT_GT_F	ACGTTGGATGGACCTGAAGTTCAGCAGTAG	sf003614_01CT_GT_R	ACGTTGGATGTCTGACTGACGGAACTGATG	sf003614_01CT_GT_Pa	CATCTTTAAACCTTACAGGT
sequenom	SEQ012	sf003813_11CT	sf003813_11CT_AG_F	ACGTTGGATGAAGACATGGTTACTGACGGC	sf003813_11CT_AG_R	ACGTTGGATGTTCTCTCTCTCCCAGCCAAC	sf003813_11CT_AG02_P	CCTGACCCCAACGAGGAGCT
sequenom	SEQ012	sf004055_02CT	sf004055_02CT_AT_F	ACGTTGGATGTAGCGCAGGATTTTACGACC	sf004055_02CT_AT_R	ACGTTGGATGCCAGTGTCAGTGTCCTAATG	sf004055_02CT_AT_P	CAGGGAGACTTGTAATTCAGC
sequenom	SEQ012	sf006073_01CT	sf006073_01CT_GA_F	ACGTTGGATGAGCCTGGAGTGTTTTTTCTG	sf006073_01CT_GA_R	ACGTTGGATGAGTGGCCGTCGTCAAGTTTG	sf006073_01CT_GA_Pa	TCTGTTTGCTGCTAGTG
sequenom	SEQ013	sf000002_01AG	sf000002_01AG_AG_F	ACGTTGGATGGCACAAGAGAGCTATATTAC	sf000002_01AG_AG_R	ACGTTGGATGGAGAGCCCTTCACAGTTTAG	sf000002_01AG_AG_P	CCTCCCTTTGCTACGTGGATA
sequenom	SEQ013	sf000017_01CT	sf000017_01CT_GC_F	ACGTTGGATGCACAATGCAGATCCCAGATG	sf000017_01CT_GC_R	ACGTTGGATGGGCCTCCAGCTTTAATTGTG	sf000017_01CT_GC02_Pa	ATAAGTACTCTCTGAATGGGAT
sequenom	SEQ013	sf000021_01AG	sf000021_01AG_CA_F	ACGTTGGATGTAATGACCTCTAGCCACCTC	sf000021_01AG_CA_R	ACGTTGGATGATAAACAGCTGTGTGGGACG	sf000021_01AG_CA_Pa	GGGATGGGTCTGCTGGAT
sequenom	SEQ013	sf000086_01CT	sf000086_01CT_CT_F	ACGTTGGATGTATCCATAGGGCAAGCGTAG	sf000086_01CT_CT_R	ACGTTGGATGGCGAAGTCGTGACACAAAAG	sf000086_01CT_CT_P	AAACGTGCTTATAGACAAC
sequenom	SEQ013	sf000088_01AC	sf000088_01AC_GA_F	ACGTTGGATGGGTTTGCGAGAACACAAAGC	sf000088_01AC_GA_R	ACGTTGGATGTCCACTGGTCTGGTGTATTC	sf000088_01AC_GA_Pa	CAACTATATTGTATTTAATAAACTTGGGAC
sequenom	SEQ013	sf000119_01AG	sf000119_01AG_AG_F	ACGTTGGATGCAGCAGCTATTGTTGCTATC	sf000119_01AG_AG_R	ACGTTGGATGACAAACAGTGTCCTAGGGAG	sf000119_01AG_AG_P	GAATTACTTACAGCAGACTGAATACAGT
sequenom	SEQ013	sf000139_01CG	sf000139_01CG_GT_F	ACGTTGGATGCCTAATGTTACGAAATACCC	sf000139_01CG_GT_R	ACGTTGGATGGCGGTCCTTTTGACTGCAAC	sf000139_01CG_GT_Pa	CAAGTATAAGCCTAATAGACACATTT
sequenom	SEQ013	sf000157_01AT	sf000157_01AT_CA_F	ACGTTGGATGAGATCAGCAGAGCAGACAAG	sf000157_01AT_CA_R	ACGTTGGATGCGATATTTTCCCATGGCCTG	sf000157_01AT_CA_P	TGGAGTTGTGTAGTCTAA
sequenom	SEQ013	sf000175_01CT	sf000175_01CT_GT_F	ACGTTGGATGTTGTCCCAACAGAAACACAG	sf000175_01CT_GT_R	ACGTTGGATGAGACGATTGGGCCAATTTCC	sf000175_01CT_GT02_P	CGGCCAATTTCCCAAAGCA
sequenom	SEQ013	sf000392_01AC	sf000392_01AC_CT_F	ACGTTGGATGAGCCGTTTTGAAGCTGGTCC	sf000392_01AC_CT_R	ACGTTGGATGGTGATGCACCTCATTTGAGC	sf000392_01AC_CT_P	TTGGAATGCAGTTGGT
sequenom	SEQ013	sf000583_02CT	sf000583_02CT_TC_F	ACGTTGGATGTTAGTGCTACCACTAGAGGG	sf000583_02CT_TC_R	ACGTTGGATGTTAGACTGGGCTTCTCTTCC	sf000583_02CT_TC_Pa	TCCACAGTTAGCTGCAGGCGTGC
sequenom	SEQ013	sf000717_02AG	sf000717_02AG_TC_F	ACGTTGGATGGTTTCTGTTTCTCATCTCAC	sf000717_02AG_TC_R	ACGTTGGATGGTCTTGTAGGAAGTCAGATG	sf000717_02AG_TC02_Pa	CTGTGGCTTTGAAATGTATCC
sequenom	SEQ013	sf000747_02CT	sf000747_02CT_CT_F	ACGTTGGATGATGTTGTATTCTGACACTGG	sf000747_02CT_CT_R	ACGTTGGATGAAAGTCACTCTTAAGGCCCC	sf000747_02CT_CT_P	AGTAAGGCCCCATGTAGGTT
sequenom	SEQ013	sf000978_02CT	sf000978_02CT_GA_F	ACGTTGGATGGCGAACCGTCTTGCATTAAC	sf000978_02CT_GA_R	ACGTTGGATGGAAACGTCTCTAGTTGCCAG	sf000978_02CT_GA_Pa	TTGCTAGAACATTGCCA
sequenom	SEQ013	sf001164_02GT	sf001164_02GT_TC_F	ACGTTGGATGAACTAGCTGGAAATGATCAC	sf001164_02GT_TC_R	ACGTTGGATGAAACCTCTGACGTCAGTAGC	sf001164_02GT_TC_Pa	GGAGATCACTGGACAAGTTTAAGG
sequenom	SEQ013	sf001692_03CG	sf001692_03CG_CT_F	ACGTTGGATGTTCCTGTGGCCTTCTGTTTC	sf001692_03CG_CT_R	ACGTTGGATGGTAGGAAGAACAAAGGACGC	sf001692_03CG_CT_P	CAAAGTACGCTAACAAAGAGAAACGCTGAT
sequenom	SEQ013	sf002333_01AC	sf002333_01AC_AG_F	ACGTTGGATGTGCCTTATCTGTATACGCTC	sf002333_01AC_AG_R	ACGTTGGATGTGCATCACACTACTGTACAC	sf002333_01AC_AG_P	GGGGAGGAACTGGAAATTGAAGTGC
sequenom	SEQ013	sf003018_09CT	sf003018_09CT_AG_F	ACGTTGGATGGTTCAGCACTGCCTCATTTC	sf003018_09CT_AG_R	ACGTTGGATGGGTATGACGTTCACTCTGAC	sf003018_09CT_AG_P	CCCTATCCACCAGGTGGCT
sequenom	SEQ013	sf003105_01CT	sf003105_01CT_TC_F	ACGTTGGATGCTAACTTTGCATCCGCACTG	sf003105_01CT_TC_R	ACGTTGGATGACAACTCCATGCACAAGGAC	sf003105_01CT_TC_Pa	GTCACGGAACATTTTACCAG
sequenom	SEQ013	sf003208_01CT	sf003208_01CT_TC_F	ACGTTGGATGTACACAAACCTTAGCTCCCC	sf003208_01CT_TC_R	ACGTTGGATGTGTACAAAGAGAGGATGCGG	sf003208_01CT_TC_Pa	CTCACAGGCGGCCATCTTAATTT
sequenom	SEQ013	sf003442_01CG	sf003442_01CG_AG_F	ACGTTGGATGAGCACAGCCAACCACTTTAC	sf003442_01CG_AG_R	ACGTTGGATGAAGCTGTCTCCTGGGCTTAA	sf003442_01CG_AG_P	GGTTACATTATGCAGGGCATC
sequenom	SEQ013	sf003538_01AG	sf003538_01AG_GT_F	ACGTTGGATGCAAATCAAGCACTTCAAATG	sf003538_01AG_GT_R	ACGTTGGATGTGGTGCTACTTCACATGGAG	sf003538_01AG_GT_Pa	CCTCCTAGAGGCTACATATGCAAGACAA
sequenom	SEQ013	sf003697_01CT	sf003697_01CT_CA_F	ACGTTGGATGCAAAAATGTGTGTCGTTGGG	sf003697_01CT_CA_R	ACGTTGGATGATGGGCAAAGAGGGCTATTC	sf003697_01CT_CA_Pa	CTGCAAAGAGGGCTATTCTAAAAT

sequenom	SEQ013	sf003715_01AT	sf003715_01AT_CT_F	ACGTTGGATGTAGATGTTCAGGCCAAGACC	sf003715_01AT_CT_R	ACGTTGGATGCTTACAAGTCTGGAAATGTG	sf003715_01AT_CT02_P	TGTGATAATGCATGGAATGC
sequenom	SEQ013	sf003726_01CT	sf003726_01CT_GT_F	ACGTTGGATGCCATCATTCAACACTCCTCC	sf003726_01CT_GT_R	ACGTTGGATGTGCTCATCTAGCAACTCAGG	sf003726_01CT_GT02_P	CCAAACTCAGGTGCTTTCATTA
sequenom	SEQ013	sf004016_01AT	sf004016_01AT_AG_F	ACGTTGGATGCCAGTCTGTCATAACCCATC	sf004016_01AT_AG_R	ACGTTGGATGGAACACAGAGACAATGGAGG	sf004016_01AT_AG_P	GGGGAATTTTGAGGTGTGTAATTACAACA
sequenom	SEQ013	sf004099_01CG	sf004099_01CG_TA_F	ACGTTGGATGGGAAACACATATCTGGTGAG	sf004099_01CG_TA_R	ACGTTGGATGGTGGAGAGTTCATCATTAGC	sf004099_01CG_TA_Pa	GGGCAGATCTGTTGCCAATTGG
sequenom	SEQ013	sf004299_15GT	sf004299_15GT_CT_F	ACGTTGGATGGGGACTCCACACATTACAAA	sf004299_15GT_CT_R	ACGTTGGATGAACTAACAGTTAGCATGGTG	sf004299_15GT_CT_P	GAAAAGCTGAACAAAGTAATTTTCTA
sequenom	SEQ013	sf005138_03AG	sf005138_03AG_CA_F	ACGTTGGATGGGAAGGCATCCTCAAAATTC	sf005138_03AG_CA_R	ACGTTGGATGCTCATCACTGTATCGTGTGC	sf005138_03AG_CA_Pa	GGGGTAGTGTGCATTTCTCCAGGGTCGC
sequenom	SEQ014	sf004149_01AG	sf004149_01AG_GC_F	ACGTTGGATGGGACTATGTACAACAAGTGG	sf004149_01AG_GC_R	ACGTTGGATGATGATGGCATGACTATGAGG	sf004149_01AG_GC_Pa	CCCTGTATTTTCATCCATATCGGAT
sequenom	SEQ014	sf004155_01AG	sf004155_01AG_GT_F	ACGTTGGATGGCTTTTGCTTTTACTACGGC	sf004155_01AG_GT_R	ACGTTGGATGGATGCTGGTAGCCTAGAATA	sf004155_01AG_GT_P	CAGCCTAGAATAAATGTTATGGAAG
sequenom	SEQ014	sf004209_01GT	sf004209_01GT_AT_F	ACGTTGGATGCCTCACGCTAACAATTACCC	sf004209_01GT_AT_R	ACGTTGGATGATCCCCAACACTCCTTTGTC	sf004209_01GT_AT_P	CACGCCTTTGTCGTACTATAGGC
sequenom	SEQ014	sf004240_01AG	sf004240_01AG_GA_F	ACGTTGGATGTAAACTCCCCCCTCATACTC	sf004240_01AG_GA_R	ACGTTGGATGTATGACACCTCCAATGTGGG	sf004240_01AG_GA_Pa	CAGACAAAAAACTGCTCGA
sequenom	SEQ014	sf004252_02GT	sf004252_02GT_GT_F	ACGTTGGATGGTTCATGTCTCAGACACTGG	sf004252_02GT_GT_R	ACGTTGGATGTGAGGAGGTATTAAGTCCAG	sf004252_02GT_GT_P	TGAAGGCTGCTGTGGCTCTGCT
sequenom	SEQ014	sf004257_02CG	sf004257_02CG_GA_F	ACGTTGGATGTTATGTTCCACAAGTGGCCC	sf004257_02CG_GA_R	ACGTTGGATGTTAACATGGCAAGACAGAAC	sf004257_02CG_GA_Pa	AGACAGAACAAATTCTTATTTACAA
sequenom	SEQ014	sf004260_01AT	sf004260_01AT_TC_F	ACGTTGGATGCAGAACTGGCAGATGGAAAG	sf004260_01AT_TC_R	ACGTTGGATGATTTGTCTTCTGCCGTTCCC	sf004260_01AT_TC_Pa	TCTTTCCCAATGCCCACAA
sequenom	SEQ014	sf004387_01AG	sf004387_01AG_TC_F	ACGTTGGATGTCATTAGTGCTGCATCTGCG	sf004387_01AG_TC_R	ACGTTGGATGCCTACGTGTTAGATACAGGC	sf004387_01AG_TC_Pa	CTGCAACATTCCCAGTACAC
sequenom	SEQ014	sf004426_01AG	sf004426_01AG_CT_F	ACGTTGGATGGAAGCACTGCTTTGTTACGG	sf004426_01AG_CT_R	ACGTTGGATGAGCAAAGCCCTACATCTCTG	sf004426_01AG_CT_P	CTGTTCCTGAATACAGGCAGCCTACA
sequenom	SEQ014	sf004438_02CT	sf004438_02CT_CT_F	ACGTTGGATGACTGGACCTGGAAAGATTGG	sf004438_02CT_CT_R	ACGTTGGATGGTTAGTAGCCTAATTCACAG	sf004438_02CT_CT_P	GGTAACAGTAAACAATAGAGCTAGA
sequenom	SEQ014	sf004529_01CT	sf004529_01CT_AT_F	ACGTTGGATGGATCTCATTTTAGCAGCGAC	sf004529_01CT_AT_R	ACGTTGGATGGGCCAGATATTGCAATTGCG	sf004529_01CT_AT_P	CTCCATCAGACAAGTGAC
sequenom	SEQ014	sf004545_01AC	sf004545_01AC_CA_F	ACGTTGGATGCCTTATATTAAACAAACCAG	sf004545_01AC_CA_R	ACGTTGGATGTGTCTTAGTGTTGGTGTGCC	sf004545_01AC_CA_Pa	TTTTATTGTAATTTATTTTGTTTACAATT
sequenom	SEQ014	sf004562_01AG	sf004562_01AG_CA_F	ACGTTGGATGTCAAAGTGCACAGTGAGGAG	sf004562_01AG_CA_R	ACGTTGGATGTAACCTATTGGCTAGCCCAG	sf004562_01AG_CA_Pa	GTTAGGAATGGTCAACACAG
sequenom	SEQ014	sf004632_01AG	sf004632_01AG_CT_F	ACGTTGGATGGAAAGACAAATGTTTCTTTCG	sf004632_01AG_CT_R	ACGTTGGATGAGAGTAGGTGTATAACGTGG	sf004632_01AG_CT_P	CAACGATAACTAAGAAGTAGAA
sequenom	SEQ014	sf004642_07AG	sf004642_07AG_AG_F	ACGTTGGATGCGTCCATAATGTCAACAGGG	sf004642_07AG_AG_R	ACGTTGGATGACCTCTGGTTTGGAGTTGTG	sf004642_07AG_AG_P	GGGAGGGGTGATTGAACTGCAC
sequenom	SEQ014	sf004684_01AG	sf004684_01AG_AT_F	ACGTTGGATGGTAGGTTGGTAAGACTGGAG	sf004684_01AG_AT_R	ACGTTGGATGAACTGACATGCCAAGGATCG	sf004684_01AG_AT_P	ACTCATATCGACCATATATTGTGTTCCC
sequenom	SEQ014	sf004732_01CG	sf004732_01CG_CT_F	ACGTTGGATGTTCAACAGACTTTTTCCCGC	sf004732_01CG_CT_R	ACGTTGGATGAGATTGCCTCTGACTGTTCC	sf004732_01CG_CT_P	TTCCCCACGTTCTTTG
sequenom	SEQ014	sf004762_01AT	sf004762_01AT_CT_F	ACGTTGGATGTCGCTTGCAGCAATGTTGAG	sf004762_01AT_CT_R	ACGTTGGATGATAACAGCAGCAGAGGAGTC	sf004762_01AT_CT_P	CCCCTCTCATCGGCAACTAATA
sequenom	SEQ014	sf004765_01CG	sf004765_01CG_CT_F	ACGTTGGATGGCACCTGTAATGAGTGAATG	sf004765_01CG_CT_R	ACGTTGGATGGTTGTGTGACTCACATGCTG	sf004765_01CG_CT_P	TTACCACAAGGGATGACAA
sequenom	SEQ014	sf004817_02GT	sf004817_02GT_CG_F	ACGTTGGATGTGGGTGTCATAAAGAAAGGG	sf004817_02GT_CG_R	ACGTTGGATGACTGTCCCTATTCCTGTTAG	sf004817_02GT_CG_P	GACATGACTTTACCATGTTG
sequenom	SEQ014	sf004818_06CT	sf004818_06CT_CA_F	ACGTTGGATGGGGATAGCCAATCCAGATTC	sf004818_06CT_CA_R	ACGTTGGATGCATTAAGGCCACATAGCACC	sf004818_06CT_CA_Pa	CGGAGTTGGTCATTTTGG
sequenom	SEQ014	sf004848_01CT	sf004848_01CT_AT_F	ACGTTGGATGCAAGACATGGCATAAGAGGG	sf004848_01CT_AT_R	ACGTTGGATGTAACGACGAACGATTGCTTG	sf004848_01CT_AT_P	TAAAGTATCGTCCAACCC

sequenom	SEQ014	sf004870_01CG	sf004870_01CG_GA_F	ACGTTGGATGCACGCCTTACAACAGAATAG	sf004870_01CG_GA_R	ACGTTGGATGGAAGTCTGCCCAGTCAAAAG	sf004870_01CG_GA_Pa	GAGGATTAATAATTATACTGTACAAG
sequenom	SEQ014	sf004904_09CT	sf004904_09CT_CG_F	ACGTTGGATGGGCATGCAGAGTTCAATTAG	sf004904_09CT_CG_R	ACGTTGGATGAAGACCGGGCATTCAACTTG	sf004904_09CT_CG_P	TTGAAGTTGATACTGTTTCCATGGTAA
sequenom	SEQ014	sf004909_01GT	sf004909_01GT_TA_F	ACGTTGGATGGCCCTGTTAACTCCTTAGTG	sf004909_01GT_TA_R	ACGTTGGATGATGGGACAATGATCACTGAG	sf004909_01GT_TA_Pa	GAGCACTGAGATCATATGCAAAT
sequenom	SEQ014	sf004913_01GT	sf004913_01GT_CA_F	ACGTTGGATGGAAGCCAGATGTTAAGATGAC	sf004913_01GT_CA_R	ACGTTGGATGCCCCAGATTTGAGTCTGTAG	sf004913_01GT_CA_P	TTTCAAATGACAAGGCAATCTCACAA
sequenom	SEQ014	sf004975_01CG	sf004975_01CG_TC_F	ACGTTGGATGGGAAGTTTGCTGCAAATGAG	sf004975_01CG_TC_R	ACGTTGGATGAACGGCCTCGTACTCTATTC	sf004975_01CG_TC_Pa	CAGCCTCGTACTCTATTCTAGATC
sequenom	SEQ014	sf004977_01AT	sf004977_01AT_CT_F	ACGTTGGATGGCCTTGATGTTCATCAGTCC	sf004977_01AT_CT_R	ACGTTGGATGAGTCATCTTTCCCTTCCAGC	sf004977_01AT_CT_P	CCTCTCCATTTATAGACAATTTGTC
sequenom	SEQ014	sf005000_09CT	sf005000_09CT_TC_F	ACGTTGGATGATGCCGTTCTGTACCATCAC	sf005000_09CT_TC_R	ACGTTGGATGCCTAACAATTCCACAACTAC	sf005000_09CT_TC_Pa	GTCCACAACTACTACCTTATACAC
sequenom	SEQ014	sf005054_02AC	sf005054_02AC_GA_F	ACGTTGGATGCCACCCAACTACTCAATTCC	sf005054_02AC_GA_R	ACGTTGGATGTGTTCTCAATTGAGGCGCAG	sf005054_02AC_GA_Pa	GAGGCGCAGATAAGCG
sequenom	SEQ014	sf005079_01AG	sf005079_01AG_CA_F	ACGTTGGATGACCCACCATCACAACTCGAC	sf005079_01AG_CA_R	ACGTTGGATGGAACATCTGAAATATTGTCTG	sf005079_01AG_CA_P	ATTCCGATCTGTGTCTTTCCATTGCTGTG
sequenom	SEQ014	sf005248_01AG	sf005248_01AG_CT_F	ACGTTGGATGTTGCTGGGATTTACCACCAC	sf005248_01AG_CT_R	ACGTTGGATGGGGTAGCCAATCCAGATTTC	sf005248_01AG_CT_P	TTACACACTCAGGTATAGCAC
sequenom	SEQ014	sf005272_01CG	sf005272_01CG_GC_F	ACGTTGGATGAGTATGAGGGAGTGTGAGAG	sf005272_01CG_GC_R	ACGTTGGATGTTAGCCATTTTTCCTCCCCG	sf005272_01CG_GC_Pa	CCGGTGTCATGTGACT
sequenom	SEQ014	sf005409_04AT	sf005409_04AT_CA_F	ACGTTGGATGAGATGTATTGGAGTAGTGCC	sf005409_04AT_CA_R	ACGTTGGATGTCAGGCATTTACAGCCTCAC	sf005409_04AT_CA_P	TGTGGTATGGCATCAGACAGAAAT
sequenom	SEQ014	sf005410_01AG	sf005410_01AG_CA_F	ACGTTGGATGGGATGAGCAATGTCAGAGCG	sf005410_01AG_CA_R	ACGTTGGATGTATCCATGCCCAATGGGAAC	sf005410_01AG_CA_Pa	ACTCCATTCCGGCTCCA
sequenom	SEQ014	sf005420_01CG	sf005420_01CG_GA_F	ACGTTGGATGTTGAAGATGGTGATGGTCTC	sf005420_01CG_GA_R	ACGTTGGATGGATGCCATCAAGGCCAAAAC	sf005420_01CG_GA_Pa	ACACTTTAGTGTTTTAAAGCA
sequenom	SEQ015	sf000020_CT	sf000020_CT_CT_F	ACGTTGGATGGGCGATCTCTGTTCCTAATG	sf000020_CT_CT_R	ACGTTGGATGGGTAAAGTGGTAGAGGTAGC	sf000020_CT_CT_P	AGATCAGATTATTCTTTGTGAGAG
sequenom	SEQ015	sf000382_AG	sf000382_AG_GA_F	ACGTTGGATGTGGCAAAATCAAAGCCAGAC	sf000382_AG_GA_R	ACGTTGGATGGTTTGTAGACTGGCTACATC	sf000382_AG_GA_Pa	GGCTACATCTTATATCATCATCT
sequenom	SEQ015	sf000508_CT	sf000508_CT_TC_F	ACGTTGGATGGTGTGTGACATATTCTCCCC	sf000508_CT_TC_R	ACGTTGGATGTTGCTCAGAGAGCCAGAGAG	sf000508_CT_TC_Pa	GAGTTACACAGAGACGTGTCATC
sequenom	SEQ015	sf000559_AG	sf000559_AG_GA_F	ACGTTGGATGGGTGATAGTGGGACTGATAG	sf000559_AG_GA_R	ACGTTGGATGAAGAAGAGCAGGTTGCCCAG	sf000559_AG_GA_Pa	GGGTGTCCTTCTCCTTCATAGGAC
sequenom	SEQ015	sf000681_AT	sf000681_AT_AT_F	ACGTTGGATGGATACTCCCACACAAAACCC	sf000681_AT_AT_R	ACGTTGGATGGAGGTATGAGGTATTGGAGG	sf000681_AT_AT_P	GCAAGGTAAAGTATGTAGTAT
sequenom	SEQ015	sf000684_CT	sf000684_CT_TC_F	ACGTTGGATGTGTATAATGAGTAGGCCCCC	sf000684_CT_TC_R	ACGTTGGATGGCATGCTCCACTTTTGAACC	sf000684_CT_TC_Pa	CCCCTCTTTGTATAACCTATGCATACAC
sequenom	SEQ015	sf000744_AT	sf000744_AT_AT_F	ACGTTGGATGCAGTGAAGGGAACTGACCAC	sf000744_AT_AT_R	ACGTTGGATGCACTCGGTATGTTCTTCCTC	sf000744_AT_AT_P	CTATGGTCTTCCTCTGTATCTTGAGAGCTG
sequenom	SEQ015	sf001105_AG	sf001105_AG_AG_F	ACGTTGGATGTGTCTCTGAAAGCACCAATC	sf001105_AG_AG_R	ACGTTGGATGTACCACACATGGCACATGAG	sf001105_AG_AG_P	CCTCCTTATATTAATAACTCCCCTGTAGT
sequenom	SEQ015	sf001667_AG	sf001667_AG_GA_F	ACGTTGGATGGCCTCTCACACAAACTGTAG	sf001667_AG_GA_R	ACGTTGGATGAGTCACTGCAATTCACAGCC	sf001667_AG_GA_Pa	TGGGCCGGGTTTAGATT
sequenom	SEQ015	sf001831_AC	sf001831_AC_CA_F	ACGTTGGATGCCAGTACATTTCCAGCTGTG	sf001831_AC_CA_R	ACGTTGGATGTCATTGGTATCAACTGATGC	sf001831_AC_CA_Pa	CTCCTCAACTGATGCCCTACT
sequenom	SEQ015	sf002145_CG	sf002145_CG_CG_F	ACGTTGGATGTCTGACAGGTACACACACAG	sf002145_CG_CG_R	ACGTTGGATGGTCATTCTGGACAGGAGTTC	sf002145_CG_CG_P	GCAGTCTGGACAGGAGTTCCTACAGTA
sequenom	SEQ015	sf003455_AG	sf003455_AG_AG_F	ACGTTGGATGATAACCAGTAGGGCAACGTC	sf003455_AG_AG_R	ACGTTGGATGTATACGAAGTCGCTGACAAC	sf003455_AG_AG_P	GGTCGTTGATATAAATGTTAATGAATTAGC
sequenom	SEQ015	sf003520_AG	sf003520_AG_GA_F	ACGTTGGATGGATGCAGGGAGACAGTATTG	sf003520_AG_GA_R	ACGTTGGATGATGAGAGAGGATAGAAAGGG	sf003520_AG_GA_Pa	GGCAGAATGAATATCATGTCAATTA
sequenom	SEQ015	sf003994_GT	sf003994_GT_GT_F	ACGTTGGATGCCCCATGTATGTTTCTCAAG	sf003994_GT_GT_R	ACGTTGGATGTCAAGCCCCAACAATATAGG	sf003994_GT_GT_P	ACCTCTTTAAGAGCTACACATTTGT

sequenom	SEQ015	sf004013_AC	sf004013_AC_CA_F	ACGTTGGATGTGTGTTGTGCGTGCATG	sf004013_AC_CA_R	ACGTTGGATGGGATTTGGGAAGTGAACTGC	sf004013_AC_CA_P	CCTCACACTGACACCA
sequenom	SEQ015	sf004038_AG	sf004038_AG_AG_F	ACGTTGGATGAACTAGAGCGGATAACCATC	sf004038_AG_AG_R	ACGTTGGATGCCGGGTGTAAGATAACAATG	sf004038_AG_AG_P	CCCCAGGGTTAAACCGTTCC
sequenom	SEQ015	sf004090_CG	sf004090_CG_GC_F	ACGTTGGATGAATAAACCAGGCCAGAGATG	sf004090_CG_GC_R	ACGTTGGATGCGGCCGTTTTATGGTTTTGC	sf004090_CG_GC_Pa	CCGCCTGTCAACCTCTATT
sequenom	SEQ015	sf004131_AG	sf004131_AG_AG_F	ACGTTGGATGGAAAACCCCAAACCAGGAAC	sf004131_AG_AG_R	ACGTTGGATGAGATTCTGCGTGTAAACGCC	sf004131_AG_AG_P	GGGGTACGTAGAATGATGTGATTAC
sequenom	SEQ015	sf004357_CT	sf004357_CT_CT_F	ACGTTGGATGAACTGCAAACATGGCAACCC	sf004357_CT_CT_R	ACGTTGGATGATGTCTAATTCCTCCGCTCC	sf004357_CT_CT_P	TGTTGCAGCAATGATAATGTGGA
sequenom	SEQ015	sf004379_CT	sf004379_CT_TC_F	ACGTTGGATGGTGAGGTCAACTCAGAGTTC	sf004379_CT_TC_R	ACGTTGGATGGTTTCCTAGGTGACCTGGAG	sf004379_CT_TC_Pa	GACCTGGAGGTGAGCC
sequenom	SEQ015	sf004475_CT	sf004475_CT_TC_F	ACGTTGGATGCCGATGTTGTACCTTTGAGC	sf004475_CT_TC_R	ACGTTGGATGTTTGTGTCCCAAATGGCACC	sf004475_CT_TC_Pa	ATGGCACCCTATGTATAATACACTATATA
sequenom	SEQ015	sf004521_CG	sf004521_CG_CG_F	ACGTTGGATGGATCCATGTGATTTCGTGCC	sf004521_CG_CG_R	ACGTTGGATGGTGTCAGGAGAAACGTTAC	sf004521_CG_CG_P	ACATATAGTGATATAAATGATTTTGTATA
sequenom	SEQ015	sf004541_AC	sf004541_AC_CA_F	ACGTTGGATGCCCCTCTTTTCTCATTCCTC	sf004541_AC_CA_R	ACGTTGGATGCGCATGACACCACAATTGAG	sf004541_AC_CA_Pa	CCCATCTAACTAAATCCCAGACGACA
sequenom	SEQ015	sf004614_AG	sf004614_AG_AG_F	ACGTTGGATGGGAACTAGCGCTATCAATGG	sf004614_AG_AG_R	ACGTTGGATGCAGGCCTGATTTCCCACATA	sf004614_AG_AG_P	CCCTTTCCCACATAATTCCCTTCACTC
sequenom	SEQ015	sf004897_AT	sf004897_AT_TA_F	ACGTTGGATGGTGGCTATGACGGTGATTTC	sf004897_AT_TA_R	ACGTTGGATGTTTTTGCGTCTGTTTCCCCC	sf004897_AT_TA_Pa	GGCATAGCTGCCTATTAAAG
sequenom	SEQ015	sf005186_CT	sf005186_CT_TC_F	ACGTTGGATGCCTTTCTTCCAACCTCCATC	sf005186_CT_TC_R	ACGTTGGATGGTTGACCAGGGCTTTGTTAG	sf005186_CT_TC_Pa	CTTGTTAGTAACACTCCTAGA
sequenom	SEQ015	sf005258_AC	sf005258_AC_CA_F	ACGTTGGATGTTGGCTTCCTGGCTTGTAAC	sf005258_AC_CA_R	ACGTTGGATGAGAGCTCCCCCATCTGATAG	sf005258_AC_CA_Pa	CGATAGCAGAGTCGAAGTA
sequenom	SEQ015	sf005286_CT	sf005286_CT_CT_F	ACGTTGGATGAAAAAGTTGACGCGCGTCTG	sf005286_CT_CT_R	ACGTTGGATGCCACTCTTTCCCGATTTACC	sf005286_CT_CT_P	ACTTGCACGATCTACAC
sequenom	SEQ015	sf005443_CG	sf005443_CG_GC_F	ACGTTGGATGGCAATCACAACCAATTGAGC	sf005443_CG_GC_R	ACGTTGGATGTCCATCTTGGCCATCCATTC	sf005443_CG_GC_Pa	TCAAGCGTTGAAGTCAATATCT
sequenom	SEQ015	sf005501_AG	sf005501_AG_GA_F	ACGTTGGATGAGACACATCCTGCCATTTGC	sf005501_AG_GA_R	ACGTTGGATGTTTTCCAAGTACCAAAGCTG	sf005501_AG_GA_Pa	ATGTTCCTTACTCTGTGG
sequenom	SEQ015	sf005540_CT	sf005540_CT_TC_F	ACGTTGGATGCAGAGCATTTATCCCACAAC	sf005540_CT_TC_R	ACGTTGGATGTGGACATCTCAATTTCTCCC	sf005540_CT_TC_Pa	ACTTATGCAAACACTTGCAACATGACCT
sequenom	SEQ015	sf005590_CT	sf005590_CT_TC_F	ACGTTGGATGTGCAGTGTGTGACTGAAAGC	sf005590_CT_TC_R	ACGTTGGATGATGTGACATGTGACGGTGAG	sf005590_CT_TC_Pa	AGGGGAGACAGTGATAGTGGTAT
sequenom	SEQ015	sf005652_AG	sf005652_AG_GA_F	ACGTTGGATGGAGCGAGCATGAATATGA	sf005652_AG_GA_R	ACGTTGGATGTGCATTTTAGGCTGTCGCTG	sf005652_AG_GA_Pa	GACACCATTTCACTGTTGT
sequenom	SEQ015	sf005654_CT	sf005654_CT_CT_F	ACGTTGGATGATCTACCGGCACAATCTCAC	sf005654_CT_CT_R	ACGTTGGATGTAAATGACGTTTCTGGCGGG	sf005654_CT_CT_P	CTCACTATAGCCTTTGCATCACACTGTTGT
sequenom	SEQ015	sf005769_CTb	sf005769_CTb_CT_F	ACGTTGGATGGCCGCTGAAAAGAATCCTTG	sf005769_CTb_CT_R	ACGTTGGATGTGTGTATCTGGACCACACTG	sf005769_CTb_CT_P	TTTCCTCCTGAGAGCA
sequenom	SEQ015	sf006000_AG	sf006000_AG_GA_F	ACGTTGGATGATTGACACACCATAAGTAGC	sf006000_AG_GA_R	ACGTTGGATGACGTTGGCAGATGCAGTGGA	sf006000_AG_GA_Pa	GATGCAGTGGATTGAGA
sequenom	SEQ015	sf006410_AG	sf006410_AG_GA_F	ACGTTGGATGCTGTTGAAAAGGCAGTCATC	sf006410_AG_GA_R	ACGTTGGATGACTCAAGACCCTCACATAAG	sf006410_AG_GA_Pa	AACCATGTTCTATGATTTCTAATATTT
sequenom	SEQ016	sf000518_GT	sf000518_GT_GT_F	ACGTTGGATGCCACTTCACTGTTATGCTGG	sf000518_GT_GT_R	ACGTTGGATGGGCTAGTGGAAGAAGACATC	sf000518_GT_GT_P	GCTGAGAAACCATTGAGCTCAGAAATAT
sequenom	SEQ016	sf000554_CT	sf000554_CT_TC_F	ACGTTGGATGCAGGTTGACTGTGTCTCTAC	sf000554_CT_TC_R	ACGTTGGATGCTAGCCAGGTCTCTAGACAG	sf000554_CT_TC_Pa	GAGGGGAGAGACAGCATGCATGTA
sequenom	SEQ016	sf000656_AG	sf000656_AG_GA_F	ACGTTGGATGCCGACTGCATATGAATAGAG	sf000656_AG_GA_R	ACGTTGGATGTACGCGTGATTGGATTCCTG	sf000656_AG_GA_Pa	ATCCCAATAAATTATTTCTCCCAATCA
sequenom	SEQ016	sf001196_AG	sf001196_AG_GA_F	ACGTTGGATGAATGGGATACGCTGTCTGAG	sf001196_AG_GA_R	ACGTTGGATGTACAGTCACCTTTGCCATCC	sf001196_AG_GA_Pa	GCTTGCGTTAATGACCT
sequenom	SEQ016	sf001288_CT	sf001288_CT_CT_F	ACGTTGGATGAGACCAGACACAAACATGAG	sf001288_CT_CT_R	ACGTTGGATGTCCAGTCACCAGATGGATTG	sf001288_CT_CT_P	AGCTTATTCATCTGCCACA

sequenom	SEQ016	sf001359_AG	sf001359_AG_GA_F	ACGTTGGATGCCGGAGTGCTTATAGTACAG	sf001359_AG_GA_R	ACGTTGGATGCATCCCTTTCCTTTGAATAC	sf001359_AG_GA_Pa	GGCGTTGAATACACATTGTCATTTATATCA
sequenom	SEQ016	sf001812_AG	sf001812_AG_AG_F	ACGTTGGATGTGTGCAACTGCAGCTTTACG	sf001812_AG_AG_R	ACGTTGGATGCCCATCTGCTGGAACCAAC	sf001812_AG_AG_P	TGGAACCAACCCTTTC
sequenom	SEQ016	sf001916_AG	sf001916_AG_GA_F	ACGTTGGATGAAGAACAGTGTACCGGAAAG	sf001916_AG_GA_R	ACGTTGGATGCGGCAAGTTAGCTAATGTTC	sf001916_AG_GA_Pa	GTTCCGAATATGAGGCAATC
sequenom	SEQ016	sf002018_AC	sf002018_AC_CA_F	ACGTTGGATGGATTGAGGTGGAGCTGATTG	sf002018_AC_CA_R	ACGTTGGATGCCCAGGAAGGTTTTATAACTG	sf002018_AC_CA_P	ACAGGAAGGTTTTATAACTGTAATAA
sequenom	SEQ016	sf002131_AG	sf002131_AG_GA_F	ACGTTGGATGGTCATCAGTATCACATTGGC	sf002131_AG_GA_R	ACGTTGGATGCTGACACTGCTATCTTTGCC	sf002131_AG_GA_Pa	GGGGAGTCTATTAAACATGGAGTCATGTA
sequenom	SEQ016	sf002439_CT	sf002439_CT_CT_F	ACGTTGGATGTTCCACGGCCTGTAAGTAAC	sf002439_CT_CT_R	ACGTTGGATGGATAACACGCTGAGTCAACC	sf002439_CT_CT_P	CGCTGAGTCAACCTAAAGAT
sequenom	SEQ016	sf002640_AG	sf002640_AG_AG_F	ACGTTGGATGCCCTTACACAATCAGTTTGC	sf002640_AG_AG_R	ACGTTGGATGCTTGAGATGATTCCCACAGG	sf002640_AG_AG_P	TGTCAACTCTCCCTCC
sequenom	SEQ016	sf003633_AG	sf003633_AG_GA_F	ACGTTGGATGGAAGGTATTGTGAGAGACGC	sf003633_AG_GA_R	ACGTTGGATGTACACACCTCCACATGGAAC	sf003633_AG_GA_Pa	CACATGGAACATTAAAGAACAAG
sequenom	SEQ016	sf003647_CG	sf003647_CG_GC_F	ACGTTGGATGGCAATCACAACCAATTGAGC	sf003647_CG_GC_R	ACGTTGGATGTCCATCTTGGCCATCCATTC	sf003647_CG_GC_Pa	CTCTGTCAAGCGTTGAAGTCAATATCT
sequenom	SEQ016	sf003788_GT	sf003788_GT_GT_F	ACGTTGGATGTTATTGCAGTCCATTTGTG	sf003788_GT_GT_R	ACGTTGGATGCTCCATCAAGAAGTCATGGG	sf003788_GT_GT_Pa	GAATCTTGAGCTATCAGTTATCCTGTAT
sequenom	SEQ016	sf004056_CT	sf004056_CT_TC_F	ACGTTGGATGGAAGGATGTCATTGGACCAG	sf004056_CT_TC_R	ACGTTGGATGAACCTCTGGATTGTCAAGGG	sf004056_CT_TC_Pa	CTTAATCAAATCTCCCCTTGAATCTATC
sequenom	SEQ016	sf004192_AG	sf004192_AG_GA_F	ACGTTGGATGGTGTGAGAATAGCCTAGATG	sf004192_AG_GA_R	ACGTTGGATGTGGGAGTCTGCTGAATAGTC	sf004192_AG_GA_Pa	AGAAAATCTTAGGATTTTGATACCATTATA
sequenom	SEQ016	sf004214_AG	sf004214_AG_GA_F	ACGTTGGATGAACTACCTGTACCCTTTGTC	sf004214_AG_GA_R	ACGTTGGATGGGGTGTGATTTATCAGTATG	sf004214_AG_GA_Pa	CCCTTTACTCTTTCAAATTCTACTGTATGA
sequenom	SEQ016	sf004288_AG	sf004288_AG_GA_F	ACGTTGGATGGAAGCATAGTGAATTAAAC	sf004288_AG_GA_R	ACGTTGGATGCCTAACCCACTCACAATATG	sf004288_AG_GA_Pa	TTCACCCAAAAGGCATT
sequenom	SEQ016	sf004416_AC	sf004416_AC_CA_F	ACGTTGGATGTCCCGTTGTTTGATGCTTGG	sf004416_AC_CA_R	ACGTTGGATGTCCTCCATATGTATCAGCAG	sf004416_AC_CA_P	GGGGAGCAGAAATAAGACCGAT
sequenom	SEQ016	sf004423_AG	sf004423_AG_AG_F	ACGTTGGATGACACAGCAGTGAATCTTTCC	sf004423_AG_AG_R	ACGTTGGATGGGCCACCTAATCAACATTTC	sf004423_AG_AG_P	TTCCACAAGGTCTTTCCCTG
sequenom	SEQ016	sf004714_CT	sf004714_CT_CT_F	ACGTTGGATGCAGATGCAGACTCAGATTTT	sf004714_CT_CT_R	ACGTTGGATGACACATTGGCTGTGTACAGG	sf004714_CT_CT_P	ATACTGGTGCATAGAGTTGTA
sequenom	SEQ016	sf004795_CT	sf004795_CT_CT_F	ACGTTGGATGGGGATCTTTCAAGAAGCTTAG	sf004795_CT_CT_R	ACGTTGGATGCCAGCAGTTATGTCATGCAG	sf004795_CT_CT_P	AGAAATAAGGCATAACTGTTAAGAA
sequenom	SEQ016	sf004811_AT	sf004811_AT_TA_F	ACGTTGGATGCATGAAGAAAATCAAGGTCTC	sf004811_AT_TA_R	ACGTTGGATGCTCTTCAAAACAACTGTGTC	sf004811_AT_TA_Pa	AATACCCGTTATAGCATAATCGTTA
sequenom	SEQ016	sf004890_AC	sf004890_AC_CA_F	ACGTTGGATGGAACAGAGCAATACTCAGTC	sf004890_AC_CA_R	ACGTTGGATGAGGTTTCTACCCTACATTCC	sf004890_AC_CA_P	TCTATAGAATTTCTTAATATTATGTAGTT
sequenom	SEQ016	sf004917_CT	sf004917_CT_TC_F	ACGTTGGATGAGCAATAAGATATGTTGGGG	sf004917_CT_TC_R	ACGTTGGATGAATCTGTTTCCTGCCGTCTG	sf004917_CT_TC_Pa	AGGGGTAGGGGACTTGCCTGAAC
sequenom	SEQ016	sf004984_AG	sf004984_AG_GA_F	ACGTTGGATGGGGATGTGCAGACATTATTC	sf004984_AG_GA_R	ACGTTGGATGTGCACCCATAGTTATGTCAC	sf004984_AG_GA_Pa	CATAGTTATGTCACATGCTTTC
sequenom	SEQ016	sf005052_AG	sf005052_AG_GA_F	ACGTTGGATGTAGAACCGATCTGGGACTC	sf005052_AG_GA_R	ACGTTGGATGATTACACATACCCGAGACCC	sf005052_AG_GA_Pa	TACCCGTGACAATCAGATCAGA
sequenom	SEQ016	sf005168_AC	sf005168_AC_CA_F	ACGTTGGATGGGCCCAGTGGAAATGAATAG	sf005168_AC_CA_R	ACGTTGGATGTGAGTGTTACTTCTCCCGTG	sf005168_AC_CA_Pa	AATGTGGTGGATTATTGAAG
sequenom	SEQ016	sf005344_AT	sf005344_AT_TA_F	ACGTTGGATGCACAAGCGTCACATGTCTTC	sf005344_AT_TA_R	ACGTTGGATGATTAACCCCTTTTTGGGTAG	sf005344_AT_TA_Pa	TTTGGGTAGGCACAAA
sequenom	SEQ016	sf005440_CT	sf005440_CT_TC_F	ACGTTGGATGGATACAAGGAAAAAGATTGG	sf005440_CT_TC_R	ACGTTGGATGTCCACAACATGAACAGACAC	sf005440_CT_TC_Pa	CCAAATGAACAGACACAATATCAC
sequenom	SEQ016	sf005459_AC	sf005459_AC_CA_F	ACGTTGGATGAAGCATAATGGAGTCCCCTG	sf005459_AC_CA_R	ACGTTGGATGGTCTGATTTACCTCATGCAG	sf005459_AC_CA_Pa	TAACAGACACATGTTTAAAAAATG
sequenom	SEQ016	sf005663_CT	sf005663_CT_TC_F	ACGTTGGATGACAACCTGTTGATCGAGGAC	sf005663_CT_TC_R	ACGTTGGATGTCACAGAGCTGACTGATCAC	sf005663_CT_TC_Pa	TGGGTGTATGTGTGCGCGA

sequenom	SEQ016	sf005769_Cta	sf005769_CTa_CT_F	ACGTTGGATGGAGAGCTATCATCCAGATCG	sf005769_CTa_CT_R	ACGTTGGATGGCAAAACGTCTTCTAGTTGG	sf005769_CTa_CT_P	AAAGTTGGGCTTCCATAG
sequenom	SEQ016	sf006000_CT	sf006000_CT_CT_F	ACGTTGGATGTTGGCAGATGCAGTGGATTG	sf006000_CT_CT_R	ACGTTGGATGATTGACACACCATAAGTAGC	sf006000_CT_CT_P	AGAGATTTTTGCATGGGC
sequenom	SEQ017	sf000132_AG	sf000132_AG_GA_F	ACGTTGGATGAGTAACACAGATCCGCCTTG	sf000132_AG_GA_R	ACGTTGGATGATGCTTTATACACCCCGTGC	sf000132_AG_GA_Pa	GGGAAATTGGGGATTGTGTTTACTTTG
sequenom	SEQ017	sf000364_AG	sf000364_AG_GA_F	ACGTTGGATGCTTGCTCTTGTGATGAGATG	sf000364_AG_GA_R	ACGTTGGATGTCTGCTTCTAGCTCCTATGC	sf000364_AG_GA_Pa	CCATTACTAGCTCTTTCCTA
sequenom	SEQ017	sf000505_CT	sf000505_CT_CT_F	ACGTTGGATGAACCGGACGATTCCATTCAG	sf000505_CT_CT_R	ACGTTGGATGTGTTTCACTCCACGCTGTTG	sf000505_CT_CT_P	CTTTCAGGATTGTCGTCTA
sequenom	SEQ017	sf000612_AT	sf000612_AT_AT_F	ACGTTGGATGCCGACAGAGACTTCTCAAAC	sf000612_AT_AT_R	ACGTTGGATGGCAAAATGTCTAGTTGTAATG	sf000612_AT_AT_P	AAAATGTCTAGTTGTAATGACGTCA
sequenom	SEQ017	sf000754_AC	sf000754_AC_CA_F	ACGTTGGATGCAGGTTTGTGTGTTTCACCG	sf000754_AC_CA_R	ACGTTGGATGGAGGGAATAACTCTCATTCTG	sf000754_AC_CA_Pa	AACTCTCATTCTGACATTTTAC
sequenom	SEQ017	sf000899_CT	sf000899_CT_CT_F	ACGTTGGATGACCCTGATGTGCCTTATTGC	sf000899_CT_CT_R	ACGTTGGATGTGGCTGTGTCTTGAATGCTG	sf000899_CT_CT_P	ACATGATATACCACGGGTA
sequenom	SEQ017	sf000991_AT	sf000991_AT_TA_F	ACGTTGGATGGGGAATAGTCTCATAATAGGC	sf000991_AT_TA_R	ACGTTGGATGGACTCTTACACGAATAGAAC	sf000991_AT_TA_Pa	TGGATATTACATGACTATTACATGACTATT
sequenom	SEQ017	sf001353_GT	sf001353_GT_GT_F	ACGTTGGATGGAGAGTCTGAGGAAGTGTTG	sf001353_GT_GT_R	ACGTTGGATGATGGACCATAGAGCATGGAG	sf001353_GT_GT_Pa	GGCATGGAGACTCTCCTC
sequenom	SEQ017	sf003142_AG	sf003142_AG_GA_F	ACGTTGGATGTCTCTGATCAAATCTTCTC	sf003142_AG_GA_R	ACGTTGGATGCGAACTTCCATTTAGCATAG	sf003142_AG_GA_Pa	CAATTTTATATGTACATACTATCTGAT
sequenom	SEQ017	sf003899_CT	sf003899_CT_TC_F	ACGTTGGATGGCACAATGTCCAACAATTCC	sf003899_CT_TC_R	ACGTTGGATGATGTCACCCTATGACTTTGG	sf003899_CT_TC_Pa	ACCCTATGACTTTGGAAACAT
sequenom	SEQ017	sf004211_GT	sf004211_GT_GT_F	ACGTTGGATGGCCACTAGCACCAATTATAC	sf004211_GT_GT_R	ACGTTGGATGCTGGTTCAGAATGCTAAGCC	sf004211_GT_GT_Pa	CCTAAAATGCTAATATACGCTAACA
sequenom	SEQ017	sf004254_CT	sf004254_CT_CT_F	ACGTTGGATGCGTTTCTCACTTGGAAACAG	sf004254_CT_CT_R	ACGTTGGATGAGCTCAAGGTTGCTCAACTG	sf004254_CT_CT_P	TAGGGAAGAGAGATGTACT
sequenom	SEQ017	sf004319_GT	sf004319_GT_GT_F	ACGTTGGATGTATGGGACAGGAAGTCACAG	sf004319_GT_GT_R	ACGTTGGATGGGCTTGCAAATCCTAAGTGG	sf004319_GT_GT_Pa	AAGTTTGGTTAAATGGAAATAAACAT
sequenom	SEQ017	sf004353_AG	sf004353_AG_GA_F	ACGTTGGATGGGCTCAATAGCAACACACAG	sf004353_AG_GA_R	ACGTTGGATGCCTGATTAATGCCAGATCTT	sf004353_AG_GA_Pa	ACAAAACATGAGCAGC
sequenom	SEQ017	sf004373_AG	sf004373_AG_GA_F	ACGTTGGATGACGCTTAGATAAGTCCCCTG	sf004373_AG_GA_R	ACGTTGGATGTGAGGTGACAAGGGATACAG	sf004373_AG_GA_Pa	CCTATTGATTTTTATGGGTGACCTAAC
sequenom	SEQ017	sf004560_GT	sf004560_GT_GT_F	ACGTTGGATGGTGCTGAATTCCTGAAGAAC	sf004560_GT_GT_R	ACGTTGGATGAGATGGGTGAGGGATATTTG	sf004560_GT_GT_P	GAATTGAGCATGGCAGC
sequenom	SEQ017	sf004583_AC	sf004583_AC_CA_F	ACGTTGGATGAGCAGTGGGCTAATCCAAAG	sf004583_AC_CA_R	ACGTTGGATGGAGTAAGGAAACCCATAGCC	sf004583_AC_CA_Pa	GCTAACGTTACTTACTTTTAAAT
sequenom	SEQ017	sf004624_CT	sf004624_CT_CT_F	ACGTTGGATGGAACCAGTTTGCTACAGCAC	sf004624_CT_CT_R	ACGTTGGATGCACTAAGGTCACTTGCAAAC	sf004624_CT_CT_P	TCCTGTTGTTGCATGAT
sequenom	SEQ017	sf004651_AG	sf004651_AG_AG_F	ACGTTGGATGAAAGGGTCTCTGTGATGGTG	sf004651_AG_AG_R	ACGTTGGATGCGAGAGAGAGAAACAACACG	sf004651_AG_AG_P	CGTTATACAAGAAAACAACAGATC
sequenom	SEQ017	sf004685_AGa	sf004685_AGa_AG_F	ACGTTGGATGCTCAGACAGATCCACTGAAC	sf004685_AGa_AG_R	ACGTTGGATGCACTAACAAAGCCAGTGTGC	sf004685_AGa_AG_P	GTGTGCAGTAGGGCGG
sequenom	SEQ017	sf004685_CG	sf004685_CG_GC_F	ACGTTGGATGCACTAACAAAGCCAGTGTGC	sf004685_CG_GC_R	ACGTTGGATGCTCAGACAGATCCACTGAAC	sf004685_CG_GC_Pa	GGGGTACTAGGCGATGATGCAT
sequenom	SEQ017	sf004839_CT	sf004839_CT_CT_F	ACGTTGGATGCGTGCTTTTTCTCCAAACCC	sf004839_CT_CT_R	ACGTTGGATGTGTCCAAAAGTGCTTGGCAG	sf004839_CT_CT_P	GGCTTGGCAGGTCATATTTGATAA
sequenom	SEQ017	sf004938_CT	sf004938_CT_TC_F	ACGTTGGATGCCAATTATGCTTTGCTTTTG	sf004938_CT_TC_R	ACGTTGGATGCTCAACTGTGAATGGTCCTG	sf004938_CT_TC_Pa	AGTTTGGATTTGGCTTCACGTTAATCGT
sequenom	SEQ017	sf005311_CG	sf005311_CG_CG_F	ACGTTGGATGGTTACTTGTGTAGTGGAGTC	sf005311_CG_CG_R	ACGTTGGATGTGGTTAGCTTCACCTACCTG	sf005311_CG_CG_P	GCATGGTAGTAACATGAGTTG
sequenom	SEQ017	sf005440_AG	sf005440_AG_GA_F	ACGTTGGATGTGTGTCTGTTCATGTTGTGG	sf005440_AG_GA_R	ACGTTGGATGAGCTGTAACTTCTCAGCTAC	sf005440_AG_GA_Pa	TCTGTAACTTCTCAGCTACATATTG
sequenom	SEQ018	sf000020_CT	sf000020_CT_TC_F	ACGTTGGATGGAGAAGGCCCTAATGCAATG	sf000020_CT_TC_R	ACGTTGGATGGCGATCTCTGTTCCTAATGC	sf000020_CT_TC_Pa	AGGATGCAACTGACCTGGC

sequenom	SEQ018	sf000062_CG	sf000062_CG_CG_F	ACGTTGGATGGGGTAGTAGTCTGGGTAAAG	sf000062_CG_CG_R	ACGTTGGATGCCTTGGAACAAGGATGGAAC	sf000062_CG_CG_P	ACGCCACTGAAACACACA
sequenom	SEQ018	sf000151_AT	sf000151_AT_TA_F	ACGTTGGATGTTATCATGTGAGGCTCAGGG	sf000151_AT_TA_R	ACGTTGGATGGACCTAATAGTCAACGGGAG	sf000151_AT_TA_Pa	GACGGGAGTACTCATGATGGCACA
sequenom	SEQ018	sf000310_AC	sf000310_AC_CA_F	ACGTTGGATGCTGTGGCTAAACATGTTAAGT	sf000310_AC_CA_R	ACGTTGGATGGGGAAGAGTACAAACAGAGC	sf000310_AC_CA_P	CCTGAAGAGTACAAACAGAGCTGGGAC
sequenom	SEQ018	sf000364_GT	sf000364_GT_GT_F	ACGTTGGATGTTGTAAAATGGCGTCGACAG	sf000364_GT_GT_R	ACGTTGGATGGGAAAGATCTGTTCTTGCTC	sf000364_GT_GT_P	AGGAAAGAGCTAGTAATGG
sequenom	SEQ018	sf001287_CT	sf001287_CT_TC_F	ACGTTGGATGCTGTCTATATATCGACCAGG	sf001287_CT_TC_R	ACGTTGGATGGAGACTTTAAAATCTCAAG	sf001287_CT_TC_Pa	GCTCGATATTCAATTAAGAAGCG
sequenom	SEQ018	sf001394_AT	sf001394_AT_TA_F	ACGTTGGATGAATGGGATGGGCTCTTAGTG	sf001394_AT_TA_R	ACGTTGGATGGCTATGACCTTCATGCAATG	sf001394_AT_TA_Pa	AGAGTAAATGAGGTGAGAGATTA
sequenom	SEQ018	sf001546_AC	sf001546_AC_CA_F	ACGTTGGATGTACGAGGTGTAGCTGTGATG	sf001546_AC_CA_R	ACGTTGGATGGGCTGTCAACAGACAAGTAG	sf001546_AC_CA_P	AAGTAGTCACAAACGCAACCACACACA
sequenom	SEQ018	sf001846_AG	sf001846_AG_AG_F	ACGTTGGATGCACACTAGATATGGATCACG	sf001846_AG_AG_R	ACGTTGGATGATGCTTATCGTTCCACCACC	sf001846_AG_AG_P	CCGTCGGCTTTAATATTTGACTT
sequenom	SEQ018	sf002055_AG	sf002055_AG_GA_F	ACGTTGGATGGACTGATGCATTGTCACCTG	sf002055_AG_GA_R	ACGTTGGATGTAAACCCCAGTTTGCTGTCG	sf002055_AG_GA_Pa	CCACCACTGAATCAGACAGTG
sequenom	SEQ018	sf002721_GT	sf002721_GT_GT_F	ACGTTGGATGATTAGTGATGATGGGACTGC	sf002721_GT_GT_R	ACGTTGGATGGCCTCTGGAAAAACACACTG	sf002721_GT_GT_Pa	ACTCTGGAAAAACACACTGACTATATGAA
sequenom	SEQ018	sf003016_AG	sf003016_AG_GA_F	ACGTTGGATGCCTCTATTTACAGAGGGAAG	sf003016_AG_GA_R	ACGTTGGATGGACCAAAGTCCCACAAACAC	sf003016_AG_GA_Pa	CCCCCAGCACATAACTACAGATCTTTGTC
sequenom	SEQ018	sf003382_AG	sf003382_AG_GA_F	ACGTTGGATGGCACTACTTTTGACCAGAGC	sf003382_AG_GA_R	ACGTTGGATGTCTAGCACAACGTCATAAGC	sf003382_AG_GA_Pa	AACCCTAATCCCTGTATAGTG
sequenom	SEQ018	sf003611_AC	sf003611_AC_CA_F	ACGTTGGATGGACATAGCTCAACATGTCTG	sf003611_AC_CA_R	ACGTTGGATGGCTGCAGGAGAATCTAAATG	sf003611_AC_CA_P	TGTCGATGTACCTTTATCCAGATAA
sequenom	SEQ018	sf003639_CT	sf003639_CT_CT_F	ACGTTGGATGCCGTTGTTATTTCGAGCGTG	sf003639_CT_CT_R	ACGTTGGATGAACAAACCTTTAATCCACC	sf003639_CT_CT_P	ACAAACCTTTAATCCACCATGTAACC
sequenom	SEQ018	sf003755_CT	sf003755_CT_TC_F	ACGTTGGATGGCAGGAGTACTTTAAATTGTG	sf003755_CT_TC_R	ACGTTGGATGCTGCAGCAACAGAACATGTG	sf003755_CT_TC_Pa	TGTGAATTAAGTGGATTATAATTAAATAAC
sequenom	SEQ018	sf003922_GT	sf003922_GT_GT_F	ACGTTGGATGCCACAATGTAAGACATGCAC	sf003922_GT_GT_R	ACGTTGGATGCCTTTCCTGGGTAGCTTATC	sf003922_GT_GT_P	CAAGGATTTGTGATCACATTCTCA
sequenom	SEQ018	sf003974_GT	sf003974_GT_GT_F	ACGTTGGATGTCTTTGTTCCCTACCCTTGC	sf003974_GT_GT_R	ACGTTGGATGGGTAGTCACTCTGCTCTTTC	sf003974_GT_GT_Pa	ATCTTTCCTGAAGTCCATGATC
sequenom	SEQ018	sf004008_AG	sf004008_AG_AG_F	ACGTTGGATGTCTTCAGTTCCCAGTTTAGC	sf004008_AG_AG_R	ACGTTGGATGCTAGCTGGATAAAACATGGC	sf004008_AG_AG_P	AGGATTGAATTCCATGAGACTTCTT
sequenom	SEQ018	sf004045_CT	sf004045_CT_CT_F	ACGTTGGATGTGTCTCTGTGTGATGCTGTG	sf004045_CT_CT_R	ACGTTGGATGCGGATGACATTTTCCACCTG	sf004045_CT_CT_P	GAGGAAAAACACCAACAGAAAAACAC
sequenom	SEQ018	sf004434_AT	sf004434_AT_CG_F	ACGTTGGATGTGCAAGATATGGGCTACTAC	sf004434_AT_CG_R	ACGTTGGATGCGAGTGGAAATCCAATAGCC	sf004434_AT_CG_P	GGTCTTTGCTTTTTAGACC
sequenom	SEQ018	sf004471_AG	sf004471_AG_GA_F	ACGTTGGATGGATTGCCCAGTGACATTGTG	sf004471_AG_GA_R	ACGTTGGATGGACTCCATCTTCTGGTTGGC	sf004471_AG_GA_Pa	TGGTTGGCTCTCTCCGC
sequenom	SEQ018	sf004521_CG	sf004521_CG_CG02_F	ACGTTGGATGTCACTTAATGGACTCCAGGC	sf004521_CG_CG02_R	ACGTTGGATGGTGTCAGGAGAAACGTTAC	sf004521_CG_CG02_P	ACATATAGTGATATAAATGATTTTGTATA
sequenom	SEQ018	sf004656_AC	sf004656_AC_CA_F	ACGTTGGATGATGTTTCGAAAGCAGGCTCC	sf004656_AC_CA_R	ACGTTGGATGTATCTACTGCCCGAGAAGTG	sf004656_AC_CA_Pa	GGGTTTGCTTATTAAAAGAGACATAAA
sequenom	SEQ018	sf004668_AC	sf004668_AC_CA_F	ACGTTGGATGTCACTGAGATAGCCAAGGAG	sf004668_AC_CA_R	ACGTTGGATGTCCCAGAAGTGGTTAGATTC	sf004668_AC_CA_P	AGTTAGCTGGTTTCTGACGT
sequenom	SEQ018	sf004712_CT	sf004712_CT_CT_F	ACGTTGGATGTCCACCTTGGCTTATTGCAC	sf004712_CT_CT_R	ACGTTGGATGTGCAGTTATCTTAGCCAGCC	sf004712_CT_CT_P	TTGCAGCTAACAATTAGCTAAATGACAT
sequenom	SEQ018	sf004715_AC	sf004715_AC_CA_F	ACGTTGGATGACTGTACTAGTGCACGTGAC	sf004715_AC_CA_R	ACGTTGGATGGGGTTTGGTAATGTCCCAC	sf004715_AC_CA_Pa	TGTCCCACACCCAACC
sequenom	SEQ018	sf004741_CG	sf004741_CG_CG_F	ACGTTGGATGAGATGACAGGAAATGGAGCG	sf004741_CG_CG_R	ACGTTGGATGCAGTCAATTTGCAGAGCAGG	sf004741_CG_CG_P	GTTGTTCTAGATGAAGGC
sequenom	SEQ018	sf004774_AG	sf004774_AG_AG_F	ACGTTGGATGAACCTGCAGCCTGATCAAAG	sf004774_AG_AG_R	ACGTTGGATGTGGCAATGGTATGGCACATC	sf004774_AG_AG_P	TTGGGTACGTAGCACAAGCATCTAGA

sequenom	SEQ018	sf005157_CT	sf005157_CT_CT_F	ACGTTGGATGGAACATCATTGTTGGAGCAG	sf005157_CT_CT_R	ACGTTGGATGCTTTTAAAATGGGCCTTTGC	sf005157_CT_CT_P	GATTAGAGAAACTCATGAAACA
sequenom	SEQ018	sf005160_AC	sf005160_AC_CA_F	ACGTTGGATGCTTGAAGGCTCTTACGTTGG	sf005160_AC_CA_R	ACGTTGGATGGTTGAGGTCAGAAGTAGAAG	sf005160_AC_CA_Pa	ATCAGATGGAGTTGAAGACGA
sequenom	SEQ018	sf006410_AG	sf006410_AG_GA02_F	ACGTTGGATGGCTTCAATTGGCTCCATATC	sf006410_AG_GA02_R	ACGTTGGATGTGTAAACTCAAGACCCTCAC	sf006410_AG_GA02_Pa	AAACCATGTTCTATGATTTCTAATATTT
