# Supplementary material for: Coding Gene Single Nucleotide Polymorphism Mapping and Quantitative Trait Loci Detection for Physiological Reproductive Traits in Brook Charr, Salvelinus fontinalis
Source: G3 (Bethesda). 2012 Mar 1;2(3):379–92. doi: 10.1534/g3.111.001867 (PMC3291508; doi:10.1534/g3.111.001867)
Supplement: Supporting Information [file supp_2.3.379_TableS3.pdf]

Table S3 Description and annotation of the 281 SNP (single nucleotide-polymorphism) markers used to build the linkage map in the Brook charr, *Salvelinus fontinalis*.

Ts: transition; Tv: transversion; C: coding region; NC: non coding region; S: synonymous; NS: non synonymous; LG: linkage group.

	SNP Name	Accession number	GenInfo Identifiers (GI)	Variation	Ts/Tv	C/NC	S/NS	LG
1	sf000002	BT050259	GI:209738381	A/G	Ts	NC	-	22
2	sf000017	XP_691985.3	GI:189515371	C/G	Tv	C	-	31
3	sf000020			C/T	Ts	C	NS	35
4	sf000021	BT044685.1	GI:209148638	A/C	Tv	C	-	14
5	sf000023			C/T	Ts	NC	-	-
6	sf000032			A/G	Ts	C	-	15
7	sf000062			C/G	Tv	NC	-	-
8	sf000071			A/C	Tv	NC	-	3
9	sf000078			A/T	Tv	NC	-	27
10	sf000086	XM_001921499.1	GI:189516301	C/T	Ts	NC	-	-
11	sf000088	XP_002609992.1	GI:260830085	A/G	Ts	NC	-	-
12	sf000119			A/G	Ts	NC	-	12
13	sf000124			A/G	Ts	C	-	17
14	sf000132			A/G	Ts	NC	-	13
15	sf000137	NM_200955	GI:41055673	A/T	Tv	C	NS	-
16	sf000139			G/T	Tv	NC	-	4
17	sf000151			A/T	Tv	NC	-	15
18	sf000157	NP_775368	GI:27545272	A/C	Tv	C	NS	25
19	sf000175	XP_001054847	GI:109498064	G/T	Tv	NC	-	-
20	sf000178			A/G	Ts	C	-	19
21	sf000179			C/T	Ts	NC	-	3
22	sf000191			C/G	Tv	NC	-	-

23	sf000209			C/T	Ts	NC	-	-
24	sf000228			A/T	Tv	NC	-	-
25	sf000310			A/T	Tv	NC	-	10
26	sf000324			A/C	Tv	NC	-	8
27	sf000327			A/G	Ts	NC	-	-
28	sf000364			A/G	Ts	C	NS	31
29	sf000364			G/T	Tv	C	NS	31
30	sf000382			A/G	Ts	NC	-	-
31	sf000390	BT057320	GI:221221062	C/T	Ts	NC	-	-
32	sf000392	CAAE01015004.1	GI:47226310	C/T	Ts	C	-	23
33	sf000397			C/G	Tv	NC	-	20
34	sf000428			A/T	Tv	NC	-	3
35	sf000505			C/T	Ts	C	-	1
36	sf000508			C/T	Ts	NC	-	4
37	sf000518			G/T	Tv	NC	-	-
38	sf000554	BT059059.1	GI:223648027	C/T	Ts	C	NS	2
39	sf000559	XM_001502487	GI:149728067	A/G	Ts	NC	-	-
40	sf000583	EF467299.1	GI:154795566	C/T	Ts	NC	-	8
41	sf000584	EU025714	GI:158702319	A/G	Ts	C	-	10
42	sf000612	NM_001020538	GI:66472671	A/T	Tv	NC	-	37
43	sf000633			G/T	Tv	C	-	-
44	sf000656			A/G	Ts	NC	-	20
45	sf000657			C/T	Ts	NC	-	2
46	sf000679	BT060419	GI:223673194	C/T	Ts	NC	-	-
47	sf000684	GU129140	GI:213512874	C/T	Ts	C	-	34
48	sf000706	NP_001117688	GI:185135493	A/G	Ts	NC	-	6
49	sf000717			C/T	Ts	NC	-	18
50	sf000719			A/T	Tv	NC	-	16

51	sf000744			A/T	Tv	C	NS	5
52	sf000747			C/T	Ts	NC	-	-
53	sf000754			A/C	Tv	NC	-	8
54	sf000810	BT045174	GI:209154407	C/T	Ts	NC	-	2
55	sf000854			G/T	Tv	NC	-	-
56	sf000878			C/T	Ts	NC	-	-
57	sf000891			A/C	Tv	C	NS	6
58	sf000899	EU025705	GI:158702261	C/T	Ts	NC	-	12
59	sf000905			A/T	Tv	C	-	31
60	sf000961			C/T	Ts	NC	-	5
61	sf000966			A/G	Ts	NC	-	13
62	sf000970			A/G	Ts	C	-	9
63	sf000978			A/G	Ts	NC	-	1
64	sf000991			A/T	Tv	C	-	-
65	sf001069			G/T	Tv	NC	-	-
66	sf001086	NM_001141083	GI:213513628	C/T	Ts	NC	-	-
67	sf001105			A/G	Ts	NC	-	17
68	sf001164			C/T	Ts	NC	-	-
69	sf001181			A/G	Ts	C	-	-
70	sf001184	CR391965	GI:167908776	C/T	Ts	NC	-	1
71	sf001196			A/G	Ts	NC	-	-
72	sf001284			G/T	Tv	NC	-	33
73	sf001287			C/T	Ts	C	-	-
74	sf001288			C/T	Ts	C	NS	2
75	sf001353			G/T	Tv	C	-	5
76	sf001359	GQ505860	GI:260182183	A/G	Ts	NC	-	-
77	sf001394			A/T	Tv	NC	-	6
78	sf001437			A/G	Ts	NC	-	-

79	sf001546			A/C	Tv	C	NS	28
80	sf001601			C/T	Ts	NC	-	8
81	sf001644			G/T	Tv	C	-	2
82	sf001661			G/T	Tv	C	-	-
83	sf001667			A/G	Ts	C	NS	17
84	sf001686			A/G	Ts	NC	-	-
85	sf001692			C/T	Ts	NC	-	12
86	sf001740			A/G	Ts	NC	-	7
87	sf001770			A/G	Ts	NC	-	21
88	sf001812	AC203446	GI:163955243	A/G	Ts	NC	-	9
89	sf001831			A/C	Tv	NC	-	40
90	sf001846			A/G	Ts	NC	-	30
91	sf001880	EF467300	GI:154795567	C/T	Ts	NC	-	-
92	sf001996	DQ246664	GI:80971723	A/G	Ts	NC	-	28
93	sf002018			A/C	Tv	NC	-	25
94	sf002024			A/G	Ts	NC	-	3
95	sf002033	AJ311906	GI:20873289	A/T	Tv	C	-	2
96	sf002055			A/G	Ts	C	NS	31
97	sf002131			A/G	Ts	C	-	1
98	sf002145	XM_683887	GI:292622326	C/G	Tv	C	NS	31
99	sf002155			A/G	Ts	NC	-	-
100	sf002175			C/T	Ts	NC	-	34
101	sf002203			A/G	Ts	C	-	7
102	sf002250	XM_001920907	GI:189520811	A/C	Tv	C	-	28
103	sf002333			A/G	Ts	NC	-	-
104	sf002353	NM_001141843	GI:213513502	C/T	Ts	NC	-	20
105	sf002439			C/T	Ts	NC	-	31
106	sf002633	BT072804	GI:224587929	A/C	Tv	C	-	-

107	sf002640			A/G	Ts	NC	-	21
109	sf002721			A/G	Ts	C	-	32
110	sf002948	CAAE01015039	GI:47222189	G/T	Tv	C	S	7
111	sf003018			A/G	Ts	NC	-	26
112	sf003063			A/C	Tv	NC	-	1
113	sf003076	EU025709	GI:158702304	A/G	Ts	NC	-	23
114	sf003084			A/G	Ts	C	-	4
115	sf003092			C/T	Ts	C	-	9
116	sf003105			C/T	Ts	NC	-	3
117	sf003118			A/G	Ts	NC	-	6
118	sf003141	BT056462	GI:221219345	A/T	Tv	NC	-	11
119	sf003142	CU062627	GI:150171078	A/G	Ts	NC	-	-
120	sf003197			A/G	Ts	NC	-	-
121	sf003208			C/T	Ts	NC	-	-
122	sf003334			A/G	Ts	NC	-	24
123	sf003382			A/G	Ts	C	NS	14
124	sf003407			C/T	Ts	NC	-	10
125	sf003442			A/G	Ts	C	-	24
126	sf003455			A/G	Ts	NC	-	11
127	sf003500	BT049606.1	GI:209737075	C/T	Ts	NC	-	1
128	sf003520			A/G	Ts	C	S	2
129	sf003538	AL840630	GI:41322894	G/T	Tv	NC	-	9
130	sf003560			C/T	Ts	C	S	-
131	sf003589			C/T	Ts	C	-	-
132	sf003595			A/T	Tv	NC	-	-
133	sf003601			C/T	Ts	NC	-	-
134	sf003610			A/T	Tv	C	-	1
135	sf003611			A/C	Tv	NC	-	-

136	sf003614	CAAE01014563	GI:47223303	G/T	Tv	NC	-	8
137	sf003621	BT059052	GI:223648013	C/T	Ts	NC	-	-
138	sf003633			A/G	Ts	C	S	21
139	sf003639	EF427381	GI:148362130	C/T	Ts	NC	-	30
140	sf003647			C/G	Tv	NC	-	40
141	sf003697			A/C	Tv	NC	-	37
142	sf003698			G/T	Tv	C	-	-
143	sf003700			C/T	Ts	NC	-	9
144	sf003713	XM_686639.3	GI:189530935	A/T	Tv	NC	-	25
145	sf003715	EU816603	GI:209962458	C/T	Ts	NC	-	11
146	sf003724			C/T	Ts	NC	-	-
147	sf003728			C/T	Ts	C	-	31
148	sf003755			G/T	Tv	NC	-	7
149	sf003788	XM_002660927	GI:292611089	G/T	Tv	NC	-	-
150	sf003813	EU621898	GI:187936476	A/G	Ts	NC	-	8
151	sf003836			A/T	Tv	NC	-	15
152	sf003897			A/G	Ts	NC	-	10
153	sf003899			C/T	Ts	NC	-	11
154	sf003919	GQ505860	GI:260182183	C/T	Ts	NC	-	1
155	sf003977	EU481821	GI:171474994	C/G	Tv	NC	-	-
156	sf003994			G/T	Tv	NC	-	-
157	sf004008			A/G	Ts	C	NS	-
158	sf004013	EU221180	GI:159033051	A/C	Tv	C	NS	38
159	sf004016			A/G	Ts	NC	-	2
160	sf004030	XM_689729	GI:189528958	A/T	Tv	NC	-	19
161	sf004038	NM_001165151	GI:259089170	A/G	Ts	NC	-	34
162	sf004055	XP_001921123	GI:189530039	A/T	Tv	C	NS	22
163	sf004056	EF467297	GI:154795564	C/T	Ts	C	NS	-

164	sf004064			A/C	Tv	NC	-	28
165	sf004070			A/G	Ts	NC	-	24
166	sf004090			C/G	Tv	C	NS	-
167	sf004099			A/T	Tv	NC	-	-
168	sf004112			A/G	Ts	NC	-	-
169	sf004131	NM_001173900	GI:291190431	A/G	Ts	NC	-	40
170	sf004149	FJ969490	GI:262189352	C/G	Tv	NC	-	6
171	sf004155	EU025709	GI:158702304	G/T	Tv	NC	-	-
172	sf004192			A/G	Ts	C	-	-
173	sf004209			A/T	Tv	NC	-	-
174	sf004211			G/T	Tv	NC	-	3
175	sf004214			A/G	Ts	NC	-	6
176	sf004240	EU621900	GI:187936480	A/G	Ts	NC	-	6
177	sf004252	NM_001140784	GI:213515209	G/T	Tv	NC	-	5
178	sf004254	BT071904	GI:224613219	C/T	Ts	C	-	13
179	sf004257	AB294121	GI:149773365	A/G	Ts	NC	-	3
180	sf004260			C/T	Ts	C	-	-
181	sf004288			A/G	Ts	C	-	-
182	sf004299	XM_001922165	GI:189531595	C/T	Ts	NC	-	10
183	sf004319	BT058994	GI:223647897	G/T	Tv	C	-	23
184	sf004353	NM_001124574	GI:185135642	A/G	Ts	C	S	40
185	sf004357	EU025708	GI:158702285	C/T	Ts	C	NS	4
186	sf004373			A/G	Ts	C	-	5
187	sf004379			C/T	Ts	NC	-	15
188	sf004387			C/T	Ts	C	-	-
189	sf004416	GU129140	GI:290760588	A/C	Tv	NC	-	31
190	sf004423			A/G	Ts	C	-	-
191	sf004426			C/T	Ts	NC	-	24

192	sf004438	BT045405	GI:209154869	C/T	Ts	C	NS	31
193	sf004475	EU621899	GI:187936478	C/T	Ts	C	S	25
194	sf004521			C/G	Tv	NC	-	1
195	sf004529			A/T	Tv	NC	-	38
196	sf004541			A/C	Tv	NC	-	2
197	sf004545	EF427379	GI:148362119	A/C	Tv	NC	-	-
198	sf004560			G/T	Tv	NC	-	3
199	sf004562			A/C	Tv	NC	-	7
200	sf004583	NM_001013274	GI:61651703	A/C	Tv	NC	-	-
201	sf004602	XM_001922165	GI:189531595	A/G	Ts	NC	-	-
202	sf004614			A/G	Ts	C	-	13
203	sf004624	EU816603	GI:209962458	C/T	Ts	NC	-	10
204	sf004632	EU025707	GI:158702273	C/T	Ts	NC	-	-
205	sf004642			A/G	Ts	NC	-	6
206	sf004651			A/G	Ts	C	NS	6
207	sf004656			A/C	Tv	NC	-	21
208	sf004668	FJ969489	GI:262189345	A/C	Tv	C	-	38
209	sf004684			A/T	Tv	NC	-	-
210	sf004685			A/G	Ts	NC	-	39
211	sf004685			A/G	Ts	C	NS	39
212	sf004685			C/G	Tv	C	NS	-
213	sf004714	EU025714	GI:158702319	C/T	Ts	C	-	27
214	sf004715			C/T	Ts	C	-	10
215	sf004732	AB258536	GI:118596560	C/T	Ts	NC	-	29
216	sf004741			C/G	Tv	NC	-	31
217	sf004762			C/T	Ts	NC	-	18
218	sf004765			C/T	Ts	C	NS	4
219	sf004774			A/G	Ts	C	-	3

220	sf004795	BT057087	GI:221220595	C/T	Ts	NC	-	10
221	sf004811	EU025709	GI:158702304	A/T	Tv	C	NS	15
222	sf004817	EU025714	GI:158702319	C/G	Tv	NC	-	-
223	sf004818	EU481821	GI:171474994	A/C	Tv	NC	-	2
224	sf004828	AJ716203	GI:52673235	A/T	Tv	NC	-	-
225	sf004839			C/T	Ts	C	-	39
226	sf004848	BT058155.1	GI:223646219	A/T	Tv	NC	-	17
227	sf004870			A/G	Ts	NC	-	7
228	sf004890	FJ969489	GI:262189345	A/C	Tv	NC	-	-
229	sf004897			A/T	Tv	NC	-	-
230	sf004904			C/G	Tv	NC	-	-
231	sf004909	EU481821	GI:171474994	A/T	Tv	C	NS	13
232	sf004913	BT059404	GI:223648717	A/C	Tv	NC	-	4
233	sf004917	BT072749	GI:224587877	C/T	Ts	NC	-	12
234	sf004938	EU025708	GI:158702285	C/T	Ts	NC	-	9
235	sf004975	EU221176	GI:159147122	C/T	Ts	NC	-	31
236	sf004977	EU025707	GI:158702273	C/T	Ts	NC	-	-
237	sf004984	NM_001140391	GI:213511551	A/G	Ts	C	NS	5
238	sf005000	DQ246664	GI:80971723	C/T	Ts	C	NS	-
239	sf005052	BT058930	GI:223647769	A/G	Ts	C	-	-
240	sf005054	BT059612	GI:223649133	A/G	Ts	NC	-	3
241	sf005079			A/C	Tv	NC	-	-
242	sf005104	EU025714	GI:158702319	A/C	Tv	NC	-	-
243	sf005138	NM_001145584	GI:224496041	A/C	Tv	NC	-	-
244	sf005160			A/C	Tv	C	-	31
245	sf005168	BX537340	GI:51592271	A/C	Tv	C	-	2
246	sf005182	SSSAT48S1	GI:501083	A/G	Ts	NC	-	-
247	sf005182	SSSAT48S1	GI:501083	A/G	Ts	NC	-	-

248	sf005186			C/T	Ts	NC	-	39
249	sf005248	EU481821	GI:171474994	C/T	Ts	NC	-	9
250	sf005258	EU221180	GI:159033051	A/C	Tv	NC	-	-
251	sf005272	EF467298	GI:154795565	C/G	Tv	NC	-	-
252	sf005286			C/T	Ts	NC	-	-
253	sf005311	GQ505859	GI:260182171	C/G	Tv	NC	-	-
254	sf005344	GU129140	GI:290760588	A/T	Tv	C	NS	32
255	sf005399	AM748538	GI:242098051	A/T	Tv	C	NS	-
256	sf005409	CR847272	GI:55058237	A/C	Tv	C	NS	8
257	sf005410	FJ969489	GI:262189345	A/C	Tv	NC	-	-
258	sf005420			A/G	Ts	NC	-	38
259	sf005440			C/T	Ts	NC	-	21
260	sf005440			A/G	Ts	C	S	-
261	sf005443			C/G	Tv	C	-	37
262	sf005459			A/C	Tv	NC	-	1
263	sf005501	BT072704	GI:224587835	A/G	Ts	NC	-	9
264	sf005540	EU816603	GI:209962458	C/T	Ts	C	-	19
265	sf005590			C/T	Ts	C	NS	35
266	sf005652			A/G	Ts	NC	-	8
267	sf005654	NM_001165392	GI:259155245	C/T	Ts	C	-	-
268	sf005663			A/G	Ts	NC	-	1
269	sf005695			C/T	Ts	C	-	-
270	sf005769	NM_001140223	GI:213511597	A/G	Ts	NC	-	14
271	sf005769	NM_001140223	GI:213511597	C/T	Ts	NC	-	14
272	sf005917	EU221180	GI:159033051	C/T	Ts	NC	-	-
273	sf006000	EU621898	GI:187936476	C/T	Ts	C	-	35
274	sf006000	EU621898	GI:187936476	A/G	Ts	NC	-	-
275	sf006057	XM_001919059	GI:189519277	A/G	Ts	C	S	-

276	sf006073	BX927363	GI:112183280	A/G	Ts	C	NS	39
277	sf006078	BT059612	GI:223649133	A/T	Tv	NC	-	-
278	sf006081	FJ969488	GI:262189341	G/T	Tv	NC	-	33
279	sf006102	GU129139	GI:290760560	A/C	Tv	C	NS	-
280	sf006197	NM_001140134	GI:213511349	A/T	Tv	NC	-	-
281	sf006410	XM_002663717	GI:292618742	A/G	Ts	NC	-	9
