# Supplementary material for: Coding Gene Single Nucleotide Polymorphism Mapping and Quantitative Trait Loci Detection for Physiological Reproductive Traits in Brook Charr, Salvelinus fontinalis
Source: G3 (Bethesda). 2012 Mar 1;2(3):379–92. doi: 10.1534/g3.111.001867 (PMC3291508; doi:10.1534/g3.111.001867)
Supplement: Supporting Information [file supp_2.3.379_TableS5.pdf]

Table S5 Description of the consenssus and sex-specific linkage maps build using Crimap in brook charr, *Salvelinus fontinalis.* SNP: single nucleotide polymorphism; Msats: microsatellites

Map	Consensus			Female			Male		
Linkage group	Number of markers (SNP/Msats)	Total length (cM)	Average marker spacing (cM)	Number of markers (SNP/Msats)	Total length (cM)	Average marker spacing (cM)	Number of markers (SNP/Msats)	Total length (cM)	Average marker spacing (cM)
1	16 (11/5)	132.2	8.26	16 (11/5)	187	11.6	16 (11/5)	77.8	4.23
2	16 (11/5)	130.2	8.14	16 (11/5)	169.3	10.5	16 (11/5)	65.4	4.08
3	15 (10/5)	95.7	6.38	15 (10/5)	101.1	6.74	15 (10/5)	33.3	2.22
4	8 (6/2)	73.2	9.15	8 (6/2)	65.4	8.2	8 (6/2)	24.1	3.01
5	11 (6/5)	95.2	8.65	11 (6/5)	70.3	6.39	11 (6/5)	28.3	2.57
6	11 (9/2)	77.5	7.04	11 (9/2)	67.8	6.2	11 (9/2)	25.9	2.35
7	9 (6/3)	46.6	5.17	9 (6/3)	34.5	3.8	9 (6/3)	12.3	1.36
8	11 (8/3)	81.2	7.38	10 (7/3)	129.3	12.9	10 (7/3)	44.2	4.42
9	10 (9/1)	82.3	8.23	10 (9/1)	45.4	4.5	10 (9/1)	26.5	2.65
10	9 (8/1)	63.6	7.06	9 (8/1)	43.6	4.8	8 (7/1)	17.8	2.22
11	8 (4/4)	70.1	8.76	8 (4/4)	64.4	8.1	8 (4/4)	25.9	3.23
12	6 (4/2)	78.4	13.06	6 (4/2)	58.1	9.7	6 (4/2)	24.8	4.13
13	7 (5/2)	74	10.57	7 (5/2)	67.9	9.7	7 (5/2)	26.9	3.84
14	6 (4/2)	68.5	11.41	6 (4/2)	52.1	8.7	6 (4/2)	21.3	3.55
15	6 (5/1)	52.6	8.76	6 (5/1)	42.6	7.1	6 (5/1)	17.4	2.9
16	4 (1/3)	42.8	10.7	4 (1/3)	29.9	7.4	4 (1/3)	11.9	2.97
17	6 (4/2)	30.3	5.05	6 (4/2)	27.5	4.6	6 (4/2)	21.3	1.83
18	5 (2/3)	24.9	4.98	5 (2/3)	34.5	6.9	5 (2/3)	6.9	1.38
19	4 (3/1)	61.1	15.27	4 (3/1)	66.8	16.7	4 (3/1)	22.4	5.6
20	5 (3/2)	67.2	13.44	5 (3/2)	71.2	14.2	5 (3/2)	21.9	4.38
21	6 (5/1)	60.7	10.11	6 (5/1)	61.2	10.2	6 (5/1)	20.4	3.4

22	5 (2/3)	45.8	9.16	5 (2/3)	77.8	15.6	5 (2/3)	27.1	5.42
23	4 (3/1)	29.2	7.3	4 (3/1)	33.9	8.5	4 (3/1)	11.4	2.85
24	5 (4/1)	49.3	9.86	5 (4/1)	66.1	13.2	5 (4/1)	20.3	4.06
25	4 (4/0)	29.8	7.45	4 (4/0)	35.6	8.9	4 (4/0)	11.2	2.8
26	3 (2/1)	11.5	3.83	3 (2/1)	34.1	11.4	3 (2/1)	4.5	1.5
27	3 (2/1)	27.8	9.26	3 (2/1)	43.5	14.5	3 (2/1)	11.5	3.83
28	4 (4/0)	3.9	0.975	4 (4/0)	23.1	5.7	4 (4/0)	2.8	0.7
29	2 (1/1)	42.3	21.15	2 (1/1)	44.4	22.2	2 (1/1)	24.5	12.25
30	3 (2/1)	63.9	21.3	3 (2/1)	22.4	7.5	3 (2/1)	32.3	10.76
31	17 (13/4)	72.3	4.25	16 (12/4)	121.2	7.5	15 (11/4)	41.9	2.79
32	3 (2/1)	59.1	19.7	3 (2/1)	78.5	26.2	3 (2/1)	32.5	10.83
33	2 (2/0)	1.4	0.7	2 (2/0)	3.6	1.8	2 (2/0)	1.1	0.55
34	3 (3/0)	41.8	13.93	3 (3/0)	66.1	22.1	3 (3/0)	24.5	8.16
35	3 (3/0)	2.4	0.8	3 (3/0)	11.5	3.8	3 (3/0)	3.8	1.26
36	3 (2/1)	29.1	9.7	2 (1/1)	48.7	24.3	3 (2/1)	20.3	6.76
37	3 (3/0)	6.5	2.16	3 (3/0)	12.7	4.2	3 (3/0)	4.5	1.5
38	11 (5/6)	12.9	1.17	11 (5/6)	24.1	2.2	10 (4/6)	5.9	0.59
39	5 (5/0)	4.5	0.9	5 (5/0)	5.2	1.1	5 (5/0)	2.1	0.42
40	4 (4/0)	5.6	1.4	4 (4/0)	5.9	1.4	4 (4/0)	2.4	0.6
**Total**	**266**	**2047.4**	**8.31**	**263**	**2248.3**	**9.53**	**261**	**861.3**	**3.6**
